# Complex and Multidimensional Lipid Raft Alterations in a Murine Model of Alzheimer's Disease

**DOI:** 10.4061/2010/604792

**Published:** 2010-12-02

**Authors:** Wayne Chadwick, Randall Brenneman, Bronwen Martin, Stuart Maudsley

**Affiliations:** ^1^Receptor Pharmacology Unit, National Institute on Aging, National Institutes of Health, 251 Bayview Boulevard, Suite 100, Baltimore, MD 21224, USA; ^2^Miller School of Medicine, University of Miami, Miami, FL 33124, USA; ^3^Metabolism Unit, National Institute on Aging, National Institutes of Health, 251 Bayview Boulevard, Suite 100, Baltimore, MD 21224, USA

## Abstract

Various animal models of Alzheimer's disease (AD) have been created to assist our appreciation of AD pathophysiology, as well as aid development of novel therapeutic strategies. Despite the discovery of mutated proteins that predict the development of AD, there are likely to be many other proteins also involved in this disorder. Complex physiological processes are mediated by coherent interactions of clusters of functionally related proteins. Synaptic dysfunction is one of the hallmarks of AD. Synaptic proteins are organized into multiprotein complexes in high-density membrane structures, known as lipid rafts. These microdomains enable coherent clustering of synergistic signaling proteins. We have used mass analytical techniques and multiple bioinformatic approaches to better appreciate the intricate interactions of these multifunctional proteins in the 3xTgAD murine model of AD. Our results show that there are significant alterations in numerous receptor/cell signaling proteins in cortical lipid rafts isolated from 3xTgAD mice.

## 1. Introduction

Alzheimer's disease (AD) is one of the most prevalent neurodegenerative disorders amongst adults of advanced age, and it is the most common form of dementia and cognitive impairment [1, 2]. The behavioral abnormalities in AD result from dysfunction and death of neurons in brain regions involved in cognition and mood, such as the hippocampus, amygdala, and cortical regions. Progressive short-term and eventual long-term memory loss and reduced cognitive capacity are associated with two primary neurodegenerative lesions, that is, extra- and intracellular *β*-amyloid plaques, as well as neurofibrillary tangles (NFTs) composed of the microtubule protein tau [3–5]. In addition to the effects of amyloid plaques and NFTs, the lipid trafficking molecule, apolipoprotein E4 (apoE4), has also been demonstrated to be a genetic risk factor for AD [6, 7]. The AD characteristic extracellular plaques, found in both the hippocampus and cortex of AD patients, consist of 39–42 amino acid long amyloid-*β* (A*β*) peptides. These extracellular peptides are generated by digestion of a transmembrane amyloid precursor protein (APP). Proteolysis of the transmembrane APP by a set of intramembrane enzymes, *β*- (also known as BACE-1) and *γ*-secretases, is thought to be responsible for toxic A*β* creation [5]. The discovery of familial mutations in the APP gene that were strongly correlated with the presentation of AD reinforced the importance of A*β* processing in this disorder. A growing body of evidence indicates that changes in lipid and cholesterol homeostasis can influence AD progression and specifically A*β* production. One of the prime sub cellular regions of amyloidogenic APP processing is thought to be cholesterol-enriched membrane microdomains, termed lipid rafts [8]. Cellular organization of protein signaling complexes, to enhance the magnitude and fidelity of transmembrane signaling receptors, is facilitated by variations in the lipid constituents of the plasma membrane. Lipid rafts represent discontinuous regions of the plasma membrane that form functional microdomains, which constrain the association of proteins in a coherent and advantageous manner with respect to neurotransmissive signaling [9]. Disruption of the correct stoichiometry of signaling complexes within lipid rafts may underpin the etiology of many different neurodegenerative disorders [10–12]. The hypothesis that changes in the lipid composition of rafts contribute to AD pathology has gained considerable support. For example, ApoE4 has been strongly correlated with the generation of AD symptomatology. Both of the amyloidogenic processing enzymes (*β*- and *γ*-secretase), as well as APP, are all enriched in lipid raft membranes [13, 14]. Reinforcing the connection between lipid density levels and A*β* production, increasing cholesterol levels elevate the activity of both *β*-secretase (BACE-1) and *γ*-secretase [14, 15]. In addition, ganglioside lipids, which are also enriched in lipid rafts, can control the assembly of amyloid-*β* proteins [16, 17]. Changes in ganglioside composition, similar to those noted in human AD patients, are also observed in different transgenic mouse models of AD [18]. In addition to a role of the lipid components of lipid rafts in controlling amyloidogenesis, these raft environments may also affect NFTs as well. It has been demonstrated that A*β* can induce activation of the tyrosine kinase Fyn in neuronal cells, that is then recruited to lipid rafts which catalyzes phosphorylation of tyrosine residue 18 on tau [19, 20]. Association of A*β* plaques to lipid rafts can mediate recruitment of excess Fyn to the rafts, as well as further recruitment and phosphorylation of tau. These activities are thought to induce neurotoxicity via the effects of tau-induced changes in the actin cytoskeleton and receptor/cellular signaling pathways [21]. Therefore, the potential changes in the lipid composition of lipid rafts, caused by exposure to cytotoxic activities characteristic to AD, can induce profound changes in cellular signal transduction and thereby induce intracellular changes that lead to the development of AD. The complexity of protein complexes within the lipid raft environments raises considerable challenges to understanding the molecular mechanisms of AD pathophysiology in both the hippocampus and cortex of animals. Therefore, we have employed a shotgun proteomics approach, allied to advanced bioinformatic functional profiling, to gain a broad and detailed appreciation of the alterations in signaling proteins in lipid rafts in the triple-transgenic (3xTgAD) model of AD [22]. Our study demonstrates that cortical lipid rafts are profoundly affected in the 3xTgAD mice and that many of the neurophysiological deficits characteristic of AD (impaired synaptic strength, impaired learning and memory, and increased oxidative stress) can be strongly linked to changes in receptor and cell signaling events in the lipid rafts in these animals. Therefore, the lipid raft environments can be seen as one of the most important pathophysiological loci of this disorder.

## 2. Methods

### 2.1. Animals and Morris Water Maze Testing

Animal care and experimental procedures followed NIH guidelines and were approved by the National Institute on Aging Animal Care, and Use Committee. Experiments were performed using male 3xTgAD [22–24] and control male C57-BL6 mice that were maintained under a 12-hour light/12-hour dark cycle with continuous access to food and water. Water maze testing took place using a modified version of the methodology described previously [25]. Briefly, animals (*n* = 10 per group, control male C57-BL6 or male 3xTgAD, on a C57-BL6 background) received 8 days of acquisition training using a nonvisible target platform, consisting of four trials per day, with an intertrial interval of approximately 10 minutes. Each trial lasted until the animal found the platform, or for a maximum of 60 seconds; animals that failed to find the platform within 60 seconds were guided there by the experimenter. On each trial, mice were placed into the pool, facing the wall, with start locations varied pseudorandomly. Distance swam to escape the water, escape time, and swim speed were measured for either control or 3xTgAD mice using a HVS2020 automated tracking system (HVS Image, UK).

### 2.2. Isolation of Lipid Raft Detergent-Resistant Membranes

The mice were anesthetized with isoflurane, decapitated, and the brain was microdissected on ice. After removal of the cortex, the tissue was split into left and right hemisphere, half for mass spectrometry raft analysis and half to prepare lipid raft tissues for Western blot analysis. The hemicortices were washed twice in ice-cold phosphate buffered saline (PBS) and then transferred into a Tris-saline buffer supplemented with a cocktail of protease and phosphatase inhibitors (50 mM Tris-HCl, 150 mM NaCl, 5 mM EDTA, and Roche Complete-Mini (Roche Diagnostics Inc.) protease and phosphatase inhibitor cocktail, pH 7.4). Crude tissue disruption was then rapidly achieved (at 4°C) using a sonic dismembrator (Fisher Scientific Model 100) followed by a brief centrifugation (4°C, 1000 ×g, 10 minutes) to pellet cell nuclei and unbroken cells. The resultant supernatant was removed and Triton X-100 (Sigma Aldrich, USA) was added to the Tris-saline buffer to a final concentration of 1%. The supernatant membranes were then incubated at 4°C for 60 minutes in the Triton X-100 Tris-saline solution. After incubation, the supernatant solution was then added to a discontinuous gradient of 30% and 60% OptiPrep (Iodixanol, Sigma Aldrich, and U.S.A.) before centrifugation at 200 000 ×g for 16 hours at 4°C. After centrifugation, a detergent-resistant lipid band was evident in the vertical solution column. Multiple fractions of 300 *μ*l volumes were then removed from the vertical centrifugation column. Proteins were then extracted from these fractions using a proprietary ProteoExtract (EMD Biosciences) kit, according to the manufacturer's instructions. Isolated protein pellets were then prepared for mass spectrometric analysis.

### 2.3. Mass Spectrometric Protein Analysis

Protein pellets were dissolved into an ammonium bicarbonate buffer (100 mM, pH 8.5) and then reduced with dithiothreitol (500 mM: Pierce Biotechnology), alkylated with iodoacetamide (800 mM: Sigma Aldrich) and then digested with modified trypsin (5–10 *μ*g) (Promega) at 37°C for 17 hours. Proteolysis was terminated by the addition of glacial acetic acid. Tryptic peptides were then loaded onto a desalting column (360 × 200 *μ*m fused silica packed with 15 cm of C18 beads (YMC ODS-AQ, Waters)), washed with 0.1% acetic acid and eluted into sample tubes with 80% acetonitrile in 0.1% acetic acid. Sample volume was reduced to usable volumes under vacuum on a Savant SpeedVac. Samples were then transferred onto a PicoFrit (75 × 100 mm) column packed with ProteoPep II C_18_, 300 Å, 5 *μ*m particles (New Objective) connected to a nanoliquid chromatography system (Dionex, Sunnyvale, CA) online with an LTQ ion trap mass spectrometer (Thermo Finnigan, San Jose, CA). The peptides were eluted using a linear gradient of 0–65% acetonitrile over 90 minutes at a flow rate of 250 nl/min directly into the mass spectrometer, which was operated to generate collision-induced dissociation spectra (data-dependent MS/MS mode). The resultant tandem mass spectrometry data were processed using the BioWorks suite, and multiple collected spectra were used to interrogate the NCBI nonredundant mouse and Swiss-Prot protein sequence databases, using the computer algorithm SEQUEST to generate accurate protein identities. Protein genpept accession identities were then converted to Official Gene Symbol terms using NIAID-DAVID v. 6.7 (http://david.abcc.ncifcrf.gov/). The statistical analysis and validation of the search results were performed using MASCOT (Matrix Science). For protein identification, a maximum of three missed tryptic cleavages was used, including fixed modification of carbamidomethylation and variable modifications of oxidized methionine and N-terminal glutamine conversion to pyroglutamic acid in the search. Only proteins with at least two validated peptides and a total score 25 and a confidence of identification of at least 95% were considered valid for reporting. Where required, additional spectral counting was performed to determine simplistic relative quantitation in conjunction with the reported number of identified unambiguous peptides per protein. Three lipid raft fraction samples (fractions 2, 3, and 4) from each three control (nontransgenic gender/age matched C57-BL6) or Alzheimer's disease (3xTgAD) were pooled and then run in an individual random order. Proteins identified based on two unambiguous peptides that were present in at least two out of the three individual animals were employed for further expression pattern analysis.

### 2.4. Lipid Raft Band Quantification

Digitized images of centrifugal vertical fluid columns were obtained using a Canon Digital camera and were converted from Joint Photographic Experts Group (JPG) files to a TIFF (Tagged Image File Format) form using L-Process v. 2.2 (image handling software: Fuji-Film). Image densitometry was then performed using Fuji-Film Image Gauge v. 4.2. Lipid raft band intensity was represented as a relative absorbance unit (AU) value with background (B) subtraction per square pixel (px^2^) (AU-B/px^2^).

### 2.5. Western Blotting Procedures

For the examination of specific proteins in cortical cell samples (both lipid raft and nonlipid raft), aliquots were removed from centrifugal fractions from Section 2.2 and their protein concentration was determined with a standard BCA protocol. Aliquot samples for western blotting analysis were then mixed with an equal volume of Laemmli sample buffer [26]. Samples were resolved using one-dimensional gel electrophoresis (SDS-PAGE), followed by electrotransfer to polyvinylenedifluoride (PVDF: PerkinElmer, Waltham, MA). PVDF membranes were blocked for one hour at room temperature in 4% nonfat milk (Santa Cruz; Santa Cruz, CA) before application of specific primary antisera in the same nonfat milk. The presence of primary antibody reactivity with the PVDF membrane was detected by the application of a 1 : 5000 dilution of a species-specific alkaline phosphatase-conjugated secondary antibody (Sigma, St. Louis, MO). PVDF-bound immune complexes of secondary and primary antibodies were subsequently detected using enzyme-linked chemifluorescence (ECF: GE Healthcare; Pittsburgh, PA). Chemifluorescent signals from the membranes were captured and quantified using a Typhoon 9410 phosphorimager (GE Healthcare, Pittsburgh, PA). Specific primary antisera used were obtained from the following sources: flotillin-1, proline-rich tyrosine kinase 2 (Pyk2), focal adhesion kinase (FAK), G protein-coupled receptor kinase interactor-1 (GIT-1), and paxilin antibodies were obtained from BD Bioscience, San Jose, CA; Janus kinase 2 (Jak2), v-Crk avian sarcoma virus CT10 oncogene homolog (Crk), and insulin receptor substrate-1 (IRS1) antibodies were obtained from Santa Cruz Biotechnology Corporation, CA; caspase-7, FKBP12-rapamycin complex-associated protein 1/mammalian target of rapamycin (FRAP1/mTOR), and Fyn and IGF-1 receptor beta antibodies were obtained from Cell Signaling Technology, Danvers, MA); G protein-regulated inducer of neurite outgrowth 2 (Grin2) antibody was obtained from Sigma Aldrich. For the identification of nonspecific total proteins in each sample the highly sensitive protein dye, SYPRO Ruby (Invitrogen Corporation) was employed. Fixed SDS-PAGE gels were immersed in SYPRO-Ruby for 1 hour and then washed in deionized water before scanning using a Typhoon 9410 phosphorimager (GE Healthcare, Pittsburgh, PA).

### 2.6. Bioinformatic Analyses

Protein identities were converted to standard gene symbol nomenclature for simplicity of usage with the batch conversion tool of NIH Bioinformatics Resources DAVID v. 6.7 (http://david.abcc.ncifcrf.gov/). Primary protein sets (containing consistently identified lipid raft extract proteins) were organized into functional signaling pathway groups and then analyzed for their differential significance of population of these canonical signaling pathways. To compare the relative degree of association of specific signaling pathways with the control or 3xTgAD protein sets, the difference between the signaling pathways “hybrid scores” was calculated (control subtracted from 3xTgAD). The magnitude of the “hybrid score” is indicative of strength and significance of association of the input protein set with the specific signaling pathway. Signaling pathway hybrid scores were generated using a process that takes into account the significant population and potential activation of that pathway by multiplying the pathway enrichment *ratio* (percentage of proteins in a designated pathway that were also found in the experimental dataset) and the probability (*P*) that the respective pathway is significantly associated with the experimental dataset. However, to create a simple numerical value, the hybrid pathway score is calculated by multiplication of the *ratio *with the negative log − 10 of the *P* value. Each signaling pathway considered was required to contain at least two unique proteins from either control or 3xTgAD datasets and possess a *P* value of ≤.05. Unbiased network analysis was also performed on subsets of the primary protein sets that were specifically limited to transmembrane receptor proteins. The networks generated create predictions of the most likely functional interactions between proteins in a complex dataset [27]. Networks are created to indicate the most significant series of molecular interactions. The networks with the highest predictive “*scores*” possess the highest number of statistically significant “*focus molecules*”: “*focus molecules*” are proteins that are present in the most statistically-likely predicted functional network and are present in the input experimental dataset. The network “*score*” is a numerical value used to rank networks according to their degree of relevance to the input dataset. The “*score*” accounts for the number of experimental focus molecules (proteins) in the network and its size, as well as the total number of proteins in the Ingenuity Knowledge Base that could potentially be included in the specific networks. The network “*score*” is based on the hypergeometric distribution and is calculated with the right-tailed Fisher's Exact Test. Specific scientific textual associations between filtered protein sets (transmembrane receptor proteins IPA analysis) and Alzheimer's disease processes were created using latent semantic indexing (LSI) algorithms using GeneIndexer (Computable Genomix, Incorporated: https://www.computablegenomix.com/geneindexer). GeneIndexer correlates the strength of association between specific factors (proteins) in a dataset with a user-defined interrogation term. GeneIndexer employs a 2010 murine or human database of over 1 × 10^6^ scientific abstracts to perform text-protein correlation analysis. LSI facilitates the specific textual interrogation of an input dataset with a specific term, that is, Alzheimer's disease, to ascertain which of the input dataset proteins are explicitly associated with the interrogation term. Using LSI algorithms, not only is the direct interrogation term used to analyze the input dataset but also closely correlated additional terms, implicitly associated with the user-defined interrogation term, are also employed in the search patterns. A latent semantic indexing correlation score indicates the strength of association of the interrogation term and the specific proteins in the dataset. A highly relevant protein-term correlation yields a large number of explicitly/implicitly associated proteins with high LSI correlation scores. Therefore, a strong correlation between the proteins in a dataset and a specific user-defined interrogation term yields a large number of correlated proteins with high LSI correlation scores.

## 3. Statistical Analysis

Statistical analysis on multiple samples was performed using a standard nonparametric two-tailed Student's *t*-test using 95% confidence limits. Analyses were computed using built-in software in GraphPad Prism v. 3.0a (GraphPad Software Inc., La Jolla, CA). Results are expressed as means ± SE. *P* ≤ .05 was considered statistically significant. For statistical analysis using Ingenuity Pathway Analysis v. 8.5 of signaling pathways and interaction network analysis, Fisher's Exact test was employed with a *P* ≤ .05 cutoff. Network interaction scores were generated using a right-tailed Fisher's Exact Test.

## 4. Results

### 4.1. 3xTgAD Mice Demonstrate Impaired Learning and Memory Ability in the Morris Water Maze

**Figure 1:**
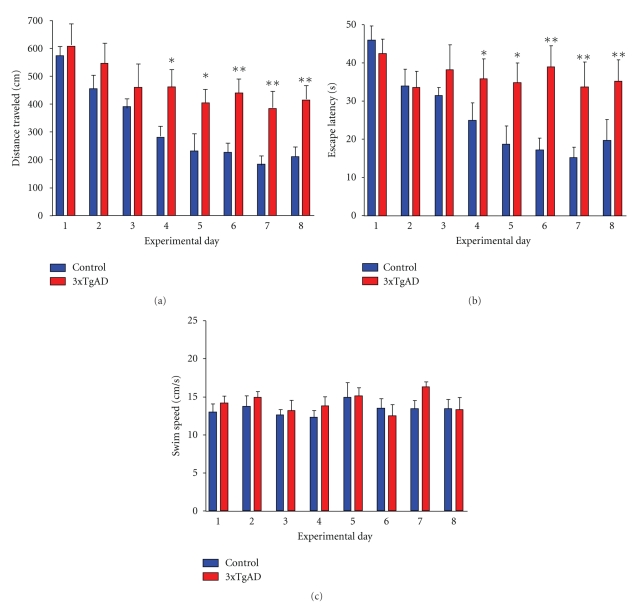
Morris Water Maze testing of control and 3xTgAD mice. (a) Distance travelled (cm) in the nonvisible probe target results for control (*n* = 10, blue bars) and 3xTgAD mice (*n* = 10, red bars) for 8 days of training. (b) Water maze escape latency (s) for days 1 to 8 of training in the nonvisible probe target. (c) Swim speed (cm/s) assessment of control and 3xTgAD animals during days 1–8 of training. *P < .05; **P < .01.

Using the nonvisible Morris water maze trial and 16-month-old male control (C57-BL6) and 3xTgAD animals (*n* = 10 for both) we noted that the 3xTgAD mice demonstrated a significant reduction in their ability to find the location of the hidden platform (Figure 1). The 3xTgAD mice demonstrated significantly longer escape latencies and distances traveled compared to the control mice, while not showing any significant difference in calculated swim speed. Retention testing (three trials one week after the initial training) of these animals (control and 3xTgAD) also demonstrated a reduced cognitive capacity of the 3xTgAD mice compared to control (data not shown).

### 4.2. 3xTgAD Mice Demonstrate a Significant Alteration in Lipid Raft Density and Protein Marker Composition

**Figure 2:**
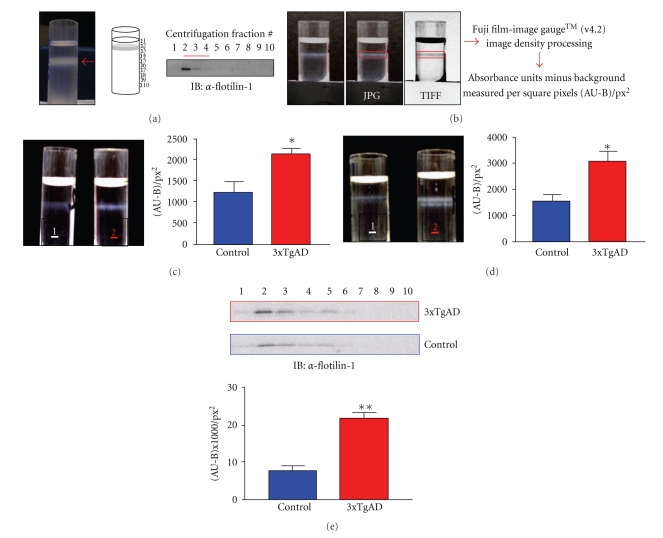
Quantification of detergent-resistant lipid rafts. (a) The pictorial panel depicts an image of iodixanol-separated detergent resistant membrane fractions (centered on red arrow), captured using a Nikon 3200 digital camera. The line diagram indicates the direction of collection of centrifugal fractions 1–10, and the associated Western blot for flotillin-1 demonstrates its enrichment in the raft fractions. (b) Captured Joint Photographic Expert Group (JPG) images were converted to a Tagged Image File Format (TIFF) version and imported to Image Gauge (v4.2) software and the specific area of interest (red box), that is, the detergent-resistant, flotillin-1-rich lipid raft band was quantified into absorbance units minus background absorbance per square pixel area ((AU-B)/px^2^) values. (c) Representative set of male 8-month-old control (1) and 3xTgAD (2) mice detergent-resistant membranes isolated from plasma membrane fractions separated using an Iodixanol gradient. The associated histogram depicts mean ± s.e. (standard error) mean detergent-resistant membrane intensity ((AU-B)/px^2^) from at least three separate control and 3xTgAD mice (*P* = .033, nonpaired, two-tailed *t*-test). (d) Representative set of male 16-month-old control (1) and 3xTgAD (2) mice detergent resistant membranes isolated from plasma membrane fractions separated using an Iodixanol gradient. The associated histogram depicts mean ± s.e. (standard error) mean detergent resistant membrane intensity ((AU-B)/px^2^) from at least three separate C57-BL6 and 3xTgAD mice (*P* = .0224, non-paired, two-tailed *t*-test). (e) Representative flotillin-1 Western blot for the lipid raft fraction series for 3xTgAD (red outline) or control (C57-BL6: blue outline) mice. The associated histogram depicts mean ± s.e. (standard error) mean fraction 2 flotillin expression intensity ((AU-B)/px^2^) from at least three separate C57-BL6 and 3xTgAD mice (*P* = .015, non-paired, two-tailed *t*-test).

Employment of the lipid raft isolation process described in the Methods section resulted in the clear visible isolation of a detergent-resistant lipid layer comprising centrifugal fractions 2–4 (Figure 1(a)). The lipid raft marker protein, flotillin-1, was demonstrated to be specifically enriched in these centrifugal fractions (2–4) (Figure 2(a)). The visual lipid density (absorbance units-background/square pixel) of the raft layers was quantified using Fuji-Film Image Gauge. Compared to control, both 8-month-old (Figure 2(c)) and 16-month-old (Figure 2(d)) 3xTgAD-derived centrifugal raft layers demonstrated a significant (8 months old *P* = .027, *n* = 3; 16 months old *P* = .031, *n* = 3) increase in buoyant detergent-insoluble density. This 3xTgAD increase in raft size, compared to control animals, demonstrated a strong association with a significant increase in expression of flotillin-1 in the raft fractions of 3xTgAD mice, especially in centrifugal fraction 2 (Figure 2(e), *P* = .017, *n* = 3). Equal levels of total protein (measured using BCA and also SYPRO gel staining) were employed for each Western blot of the raft extracts. Quantification of fraction 2 was chosen, as this reliably indicated the greatest enrichment of this lipid raft marker. Similar quantitative alterations in expression of flotillin-1 between control and 3xTgAD mice were also seen in the additional lipid raft centrifugal fractions, that is, 3 and 4. Qualitatively similar results, with respect to 3xTgAD mouse lipid raft density and flotillin-1 expression were noted in parallel experiments carried out with age-matched female mice. In addition we also noted a similar qualitative lipid raft expression of flotillin-1 trend in male human cortex tissue (data not shown). These latter data and their significance to our current data will be further addressed in subsequent manuscripts.

### 4.3. Differential Protein Expression in Lipid Rafts Isolated from 3xTgAD Mice Compared to Control Mice

**Figure 3:**
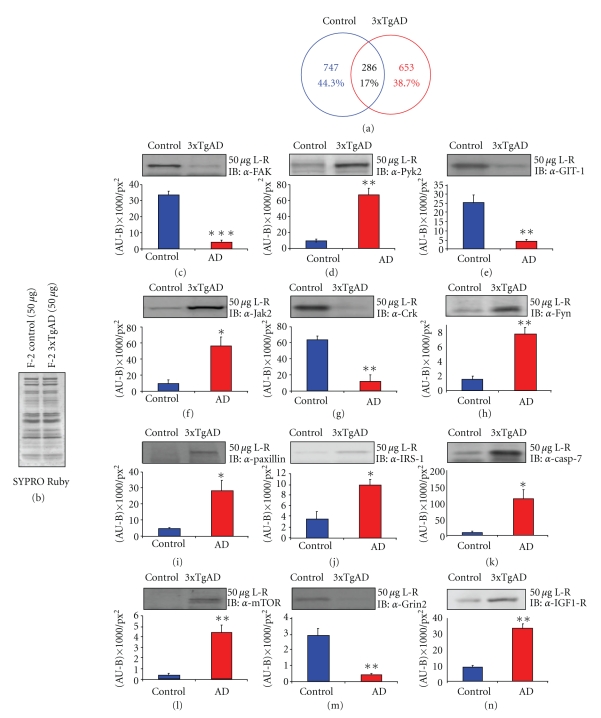
Differential protein expression in control versus 3xTgAD lipid raft extracts. (a) Proportionately drawn Venn diagram analysis of reliably identified proteins from control lipid rafts (blue line) and 3xTgAD rafts (red line). (b) Total protein loading control for centrifugal fraction 2. A total of 50 mg of fraction 2 protein was loaded and stained with SYPRO Ruby and scanned using a phosphorimager. (c)–(n). Representative western blots from multiple expression analysis experiments for differential presentation of proteins in fraction 2 extracts from control (blue) or 3xTgAD mice (red). Associated with each panel (c)–(n) the associated histograms represent the mean ± s.e. mean of protein expression intensity (measured in ((AU-B)/px_2_)) from at least three separate experiments. *P < .05; **P < .01; ***P < .001. Protein abbreviations are as follows. FAK: focal adhesion kinase; Pyk2: proline-rich tyrosine kinase 2; GIT-1: GRK interactor-1; Jak2: Janus kinase 2; Crk: v-Crk avian sarcoma virus CT10 oncogene homolog; Fyn: Fyn tyrosine kinase; IRS-1: insulin receptor substrate-1; casp 7: caspase 7; mTOR: mammalian target of rapamycin; Grin2: G protein-regulated inducer of neurite outgrowth 2; IGF-1R: insulin-like growth factor-1 receptor.

Using an un-biased proteomic analysis of replicate lipid raft extracts, we were able to identify (from at least two individual nonambiguous peptides) multiple proteins in both control and 3xTgAD cortical extracts (control, Appendix A; 3xTgAD, Appendix B). When comparing the relative differences in lipid raft protein expression, only a small minority (17%: Figure 3(a)) of identified proteins were substantively identified in both control and 3xTgAD raft samples; however many of these common proteins identified were differentially detected (see Supplementary Table 1 in Supplementary Material available online at doi:10.4061/2010/604792). To verify the relative differential expression of multiple proteins in the control versus 3xTgAD lipid raft extracts, we also performed multiple Western blot analyses of raft centrifugal fraction-2 (F-2) samples. With loading of total equal protein quantities (50 *μ*g: assessed in an unbiased manner with SYPRO-Ruby: Figure 3(b)) of either control or 3xTgAD F-2 samples, we assessed the relative differential expression of multiple proteins (Figures 3(c)–3(n)). From the Western blot analysis it was consistently demonstrated that differential qualitative protein detection in control versus 3xTgAD raft samples strongly correlated with differential semiquantitative protein expression. Hence, the absence of consistent MS-based detection of Pyk2 (Figure 3(d)), Jak2 (Figure 3(f)), Fyn (Figure 3(h)), paxilin (Figure 3(i)), IRS-1 (Figure 3(j)), caspase 7 (Figure 3(k)), mTOR/FRAP1 (Figure 3(l)) and IGF-1R (Figure 3(n)) in control raft sample correlated to their significantly lower expression in control raft F-2 samples, compared to that in 3xTgAD samples. Conversely, the absence of consistent MS-based detection of FAK (Figure 3(c)), GIT-1 (Figure 3(e)), Crk (Figure 3(g)), and Grin2 (Figure 3(m)), correlated to their significantly lower expression in 3xTgAD raft F-2 samples, compared to that in control samples.

### 4.4. Functional Signaling Cluster Analysis of Control versus 3xTgAD Lipid Raft Proteomes

**Figure 4:**
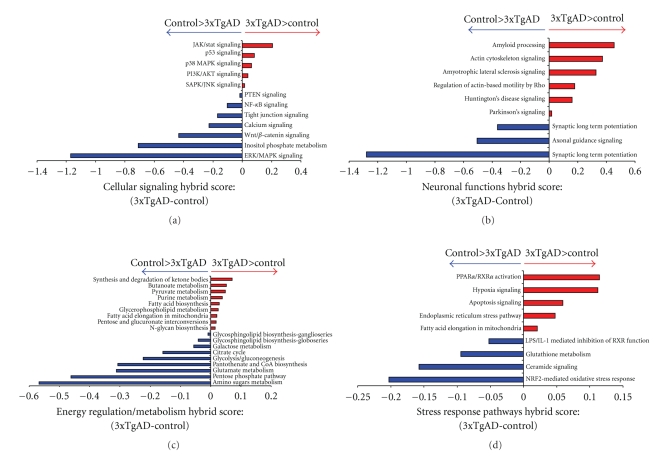
Functional pathway informatic clustering of control and 3xTgAD lipid raft proteins. (a) Subtractive representation of hybrid score generation after clustering of lipid raft proteins from control and 3xTgAD animal rafts into cellular signaling pathways. The hybrid scores were generated by multiplication of the protein enrichment ratio of the specific pathway with the negative log_10_ (−log_10_) of the probability of that enrichment (see Section 2). The data is presented as a numerical value of the control pathway hybrid score subtracted from the 3xTgAD pathway hybrid score. Pathways in which the score in 3xTgAD was greater than the control are denoted in red; pathways in which the control hybrid score is greater than the 3xTgAD hybrid score are denoted in blue. A similar depiction format is employed for differential analysis of control versus 3xTgAD Neuronal Function pathways (b), Energy Regulation/Metabolism pathways (c), and Stress Response pathways (d).

As our MS-based multidimensional protein identification process identified several hundred proteins from each control or 3xTgAD lipid raft sample, we employed a bioinformatic analysis process to assess the relative functionalities of both the control versus 3xTgAD raft protein lists. As the majority of cellular signaling processes are mediated and regulated by multiple groups of proteins interacting with each other, we clustered, in a statistically significant manner, proteins in control or 3xTgAD animal raft samples into functional signaling groups. To assess the relative changes in regulation of classical signaling pathways, we applied a subtractive approach for the pathway “*hybrid*” scores (indicative of the “activity” of the specific signaling pathway: calculated by significant expression enrichment ratio of proteins in that pathway multiplied by the negative log_10_ (−log_10_) of the probability of the pathway enrichment). For each specific common signaling pathway, our mathematical approach subtracted control pathway “hybrid” scores from the pathway “*hybrid*” scores generated from the 3xTgAD protein set. Hence, a positive result of this subtraction indicates a greater activity of this functional pathway in 3xTgAD animals, and a negative score indicates a greater activity of this functional pathway in the control animals. Analysis of pathways involved in cellular signaling (Figure 4(a): proteins and scores in associated Appendix C) demonstrated that pathways commonly associated with cell stress responses were highly activated in 3xTgAD rafts, for example “*p53 signaling*”, “*p38 mitogen-activated protein kinase (MAPK) signaling*”, and “*stress-activated protein kinase (SAPK)/JNK pathways”*. In a stark contrast, prosurvival synaptic connectivity and neurotransmissive pathways were more profoundly activated in the control mice, for example, “*tight junction signaling”*, “*calcium signaling*”, “*PTEN signaling”,* and “*Wnt/*β*-catenin signaling”*. To investigate the specific neuronal functional effects of these disparate signaling activities, we next studied the significant clustering of raft proteins into neuron-functional pathways (Figure 4(b): Appendix D). As one would expect, the 3xTgAD mice raft protein clustering revealed a considerably greater (relative to control) activation of multiple neurodegenerative neuronal processes including: “*amyloid processing”*, “*Amyotrophic lateral sclerosis”*, “*Huntington's disease signaling*” and “*Parkinson's signaling”*. In addition to the greater activation of these degenerative processes, the 3xTgAD mice also demonstrated profound changes in the significant clustering of proteins into cytoskeletal remodeling groups (“*actin cytoskeleton signaling*”, *regulation of actin-based motility by Rho'*) compared to the control mice. In accordance with our demonstration of the significant diminution of the learning and memory ability of the 3xTgAD mice, it was striking to notice the profoundly greater activation of neuron-functional pathways that control synaptic learning-dependent processes (*i.e.,* “*synaptic long-term depression”*, “*axonal guidance signaling”*,and “*synaptic long-term potentiation*”) in the control mice compared to the 3xTgAD mice. Considerable evidence from multiple experimental studies has recently underlined the importance of the regulation of energy metabolism in controlling the aging process and neurodegenerative disorders [28–30]. Upon inspection of the relative differences in the activation of energy-regulatory pathways created by clustering of control raft proteins versus 3xTgAD raft proteins, a profound functional distinction was noted (Figure 4(c): Appendix E). In control animals versus 3xTgAD, there was a considerably stronger activation of energy-generating pathways connected to the use of the primary metabolic substrate, that is, sugars (“*amino sugars metabolism”*, “*glycolysis/gluconeogenesis”*, and “*pentose phosphate pathway”*). In contrast, the energy regulatory pathways that were more strongly associated with the 3xTgAD animals involved energy derivation from alternative energy sources, for example, “*synthesis and degradation of ketone bodies*”, “*butanoate metabolism”,* and “*fatty acid biosynthesis”*. Many of the alterations in energy regulation in aging and degenerative disorders are thought to be associated with adaptive responses to the induction of cellular stresses, potentially through toxic effects of A*β*, NFTs and accumulated oxidative damage [30]. When the raft proteins from control and 3xTgAD mice were clustered into functional stress response pathways, again a stark contrast in the control- or 3xTgAD-associated pathways was demonstrated (Figure 4(d): Appendix F). In the 3xTgAD mice raft clustering it was noted that the association of energy-associated stressful and neuronal damage-related pathways (*“PPAR*α*/RXR*α* activation”*, “*hypoxia signaling”*, “*apoptosis signaling”*, and “*endoplasmic reticulum stress pathway”*) was considerably stronger than in the control mice. Indicating a correlated connection between stress response capacity and AD pathology, there was a considerably greater association of the “*Nrf2-mediated oxidative stress response pathway*” in control mice compared to the 3xTgAD. Therefore, the 3xTgAD mice may demonstrate excessive neuronal stress and damage due to the attenuated activation of such stress response pathways in lipid rafts of AD synapses. 

### 4.5. Functional Receptor Signaling Cluster Analysis of Control versus 3xTgAD Lipid Raft Proteomes

**Figure 5:**
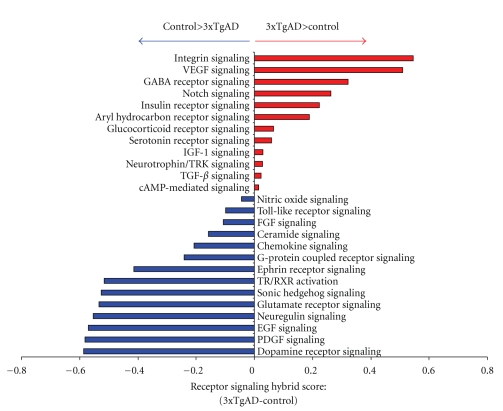
Functional receptor signaling pathway informatic clustering of control and 3xTgAD lipid raft proteins. Subtractive representation of hybrid score generation after clustering of raft proteins from control and 3xTgAD animal rafts into receptor signaling pathways. The data is presented as a numerical value of the control pathway hybrid score subtracted from the 3xTgAD pathway hybrid score. Pathways in which the score in 3xTgAD was greater than the control are denoted in red; pathways in which the control hybrid score was greater than the 3xTgAD hybrid score are denoted in blue.

**Figure 6:**
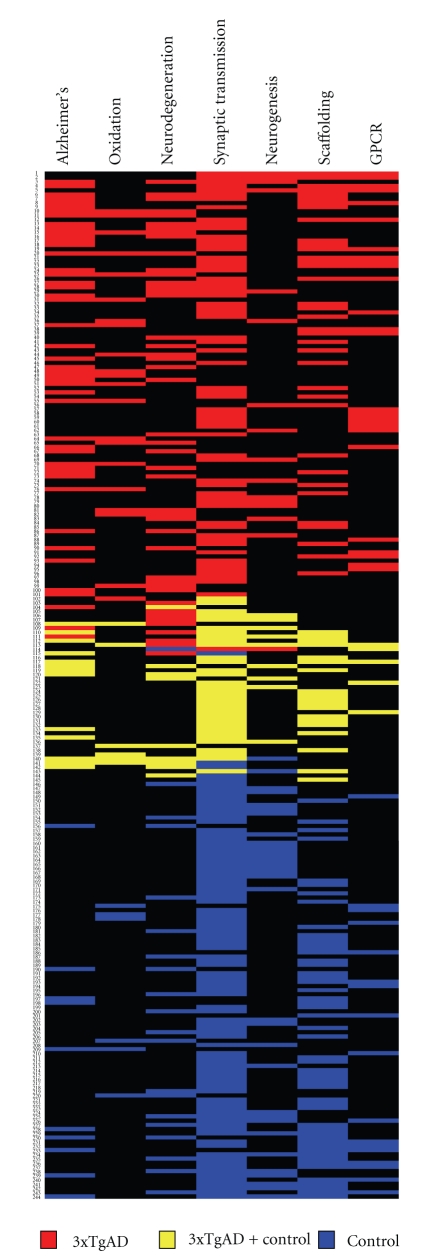
Multidimensional protein latent semantic indexing (LSI) analysis of proteins extracted from control and 3xTgAD lipid rafts. Proteins from the control or 3xTgAD extracted datasets that possessed an explicit latent semantic indexing (LSI, GeneIndexer, Computable Genomix) score in at least two of the multiple GeneIndexer interrogation terms (Alzheimer's, oxidation, neurodegeneration, synaptic transmission, neurogenesis, scaffolding, and GPCR) are represented in a heatmap format. Proteins are identified on the left side of the heatmap as an individual number (see Appendix H for key). The presence of a colored panel (3xTgAD, red: control, blue: 3xTgAD and control: yellow) on the same lateral as the numbered protein denotes explicit textual correlation of that protein with the specific vertical interrogation term.

As one of the most important functions of synaptic lipid rafts is to congregate transmembrane or juxtamembrane receptor systems [31], we next performed an in-depth investigation of the significant differential functional clustering of receptor signaling pathways between control and 3xTgAD rafts. Upon functional clustering of the raft proteins into receptor signaling pathways, strong differences in pathways association between control and 3xTgAD mice were noted (Figure 5: Appendix G). Some of the strongest differences were noted by the considerably poorer activation of growth factor-related signaling (“*PDGF signaling”*, “*EGF signaling”, and “FGF signaling”*), structural trans-synaptic receptor signaling (“*Neuregulin signaling”* and “*Ephrin receptor signaling”*), excitatory signaling (*glutamate receptor signaling*) and neurodevelopmental signaling (*Sonic hedgehog signaling*) pathways in the 3xTgAD mice, compared to the control mice. The receptor signaling profile of the 3xTgAD mice demonstrated a more profound association compared to control mice for pathways linked to inhibitory synaptic signaling (“*GABA receptor signaling*” and “*Aryl hydrocarbon receptor signaling”: *[32]), amyloid processing (“*Notch signaling” and “Integrin signaling”: *[33]), and neuronal stress (*glucocorticoid receptor signaling*). Transmembrane receptor signaling by systems including receptor tyrosine kinases or G protein-coupled receptors (GPCRs) represents one of the most important signaling mechanisms of neuronal synaptic regulation [34]. However, the activation of transmembrane receptor systems and the stimulation of their intracellular signaling cascades, especially for GPCRs, is now considered to be far more complex and intricate than initially proposed by two-state receptor models [34, 35]. Much of this additional signaling diversity is thought to arise from the additional complexity of receptor-accessory scaffolding protein modification of receptor signaling [35]. To appreciate the multiple connections between receptor (and GPCR in particular) activity and the presence of neurophysiological deficits in AD, we performed a multidimensional analysis of the proteins present in control or 3xTgAD mice. Using the novel, un-biased, bioinformatic GeneIndexer latent semantic indexing (LSI) process (https://www.computablegenomix.com/geneindexer.php), explicit correlations can be made between protein/gene factors from input datasets and their linkage (in over 1 × 10^6^ curated scientific abstracts) to a specific interrogation term, for example, Alzheimer's. The semantic indexing algorithms of GeneIndexer also allow for multidimensional correlations to be measured for terms significantly related to the interrogation term, hence providing a flexible, intelligent query process. For the control and 3xTgAD datasets, we employed multiple interrogation terms targeted to demonstrate differences between control and 3xTgAD datasets. Using the following interrogation terms: Alzheimer's, oxidation, neurodegeneration, synaptic transmission, neurogenesis, scaffolding, and GPCR, we demonstrated that, at a multidimensional interactive level, there is minimal functional cross-over between control and 3xTgAD raft samples (Figure 6: Appendix H). Only proteins that demonstrated explicit correlations (latent semantic indexing score of ≥0.1) to at least two of the interrogation terms are denoted in the multidimensional heatmap (Figure 6: Appendix H). Therefore, each of the proteins identified in the heatmap are strongly correlated with many of the connected interrogation terms and therefore show potential synergistic activity. Analysis of identified proteins in this manner, that is, selected specifically for multidimensional neurological roles, allows for an un-biased focusing on proteins that may possess keystone-like functions in the molecular signaling networks involved in neurodegeneration. With respect to specific interrogation terms, a strong validation of the technique is demonstrated by the fact that using the Alzheimer's interrogation term, 47 3xTgAD-unique multidimensional proteins were indicated while only 10 such multidimensional proteins were shown in control rafts (Figure 6). Confirming a strong role of oxidative damage, 21 3xTgAD-unique multidimensional proteins were present in the oxidation results while only 6 such control-unique were demonstrated. Interestingly and perhaps suggestive of a potential future line of AD research was the observation that considerably fewer multidimensional 3xTgAD-unique proteins were associated with the process of neurogenesis (15), compared to the 32 control-unique multidimensional proteins associated with this neuroprotective mechanism. In accordance with the important role of receptor systems in AD, we additionally noted that more 3xTgAD-unique proteins were associated with GPCRs (27) compared to control-unique GPCR-related multidimensional proteins (16).

### 4.6. Functional Interaction Networks of Receptor Signaling Proteins in Control versus 3xTgAD Lipid Raft Proteomes

**Figure 7:**
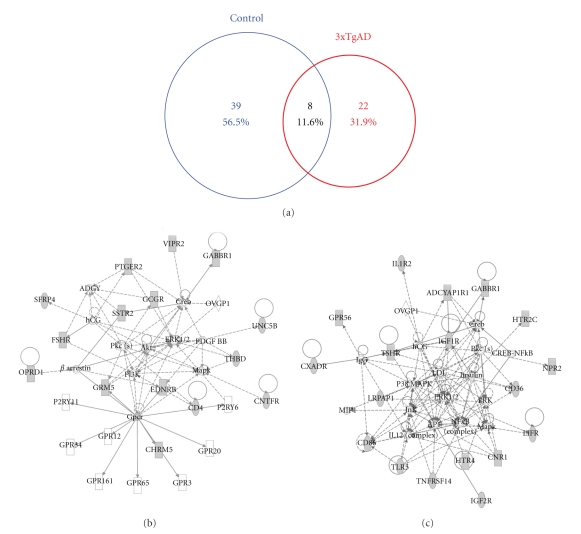
Receptor-restricted control and 3xTgAD network interaction analysis. (a) Proportionally drawn Venn diagram depicting the relative distribution between control or 3xTgAD raft samples of receptor-specific proteins filtered using IPA version 8.5 (control filtered protein list, Table 1; 3xTgAD filtered protein list, Table 2). (b) The highest scoring protein interaction network generated from IPA Network analysis (network scores and focus molecules are listed in Appendix I) of the receptor-specific control dataset. (c) The highest scoring protein interaction network generated from IPA Network analysis (network scores and focus molecules are listed in Appendix I) of the receptor-specific 3xTgAD dataset (Appendix J). A full description of the nature of interactions based on the connecting lines can be found at the following webpage linked to the IPA analysis module (https://analysis.ingenuity.com/pa/info/help/help.htm#ipa_help.htm). Dashed lines represent indirect gene interactions while solid lines represent empirically measured direct interactions. The two highest significantly scoring networks (B—control, C—3xTgAD) are based on the highest percentage of the network occupation by specific proteins (focus molecules) from the input receptor-specific datasets.

**Figure 8:**
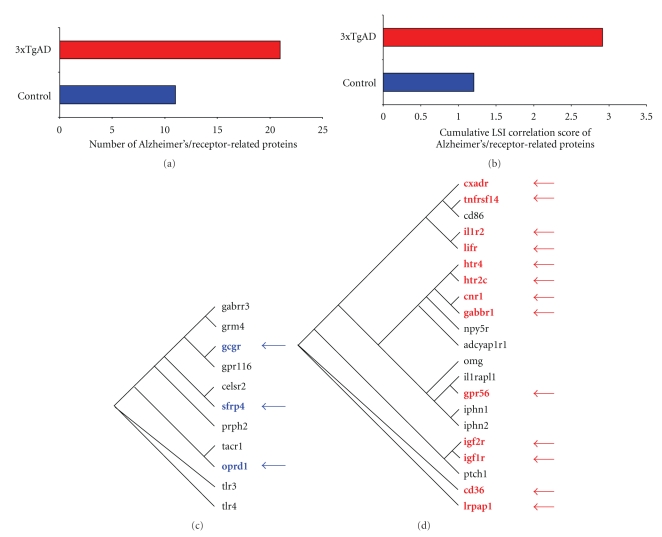
Alzheimer's disease correlation of control or 3xTgAD receptor-specific lipid raft proteins. (a) The histogram represents the number of receptor-specific raft proteins (Table 1: control, Table 2: 3xTgAD) that demonstrate an explicit LSI correlation to the term “Alzheimer's” for control (blue) or 3xTgAD (red) extracts. (b) The histogram depicts the cumulated LSI correlation scores for the Alzheimer's-related receptor-specific proteins identified in (a). panels (c) and (d) represent the phylogenetic dendrograms for the receptor-specific raft proteins linked to the interrogation term “Alzheimer's” from control (c) or 3xTgAD (d) datasets. The proteins highlighted in red or blue and indicated by an arrow were specifically clustered into the highest scoring protein interaction networks for control (Figure 7(b)) or 3xTgAD (Figure 7(c)) samples.

From our investigation of the multidimensional nature of raft proteins with respect to neurodegenerative processes, it is clear that there are many factors that are highly likely to work together in complex and intricate functional networks. To investigate the nature of the most statistically likely functional network interactions, we employed IPA network analysis of a receptor-filtered (using IPA-knowledge base data filtering, IPA v. 8,5) subset of the control or 3xTgAD raft protein datasets (Tables 1 and 2, resp.). Using these datasets, un-biased network analysis is able to predict the most likely series of functional interactions (based on empirically derived experimental evidence) that take place between the receptor-associated raft proteins. The control and 3xTgAD receptor-specific filtered datasets demonstrated a relatively minimal overlap, that is, 11.6% commonality, indicating that substantial alterations of these proteins may occur in the rafts of 3xTgAD animals (Figure 7(a)). The most statistically likely interaction network that was predicted to occur in control mice centered on neuroprotective and neurotransmissive factors such as phosphoinositide-3-kinse (PI3K), Akt-1, muscarinic GPCRs (Chrm5), and glutamate receptors (Grm5) (Figure 7(a): Appendix I). In contrast, the highest statistically scoring network from 3xTgAD receptor-specific proteins was centered on lipid-regulating factors and stress-related factors including: p38 MAPK; Jnk (c-jun N-terminal kinase), Lrpap1 (low-density lipoprotein receptor-related protein associated protein 1); LDL (low density lipoprotein), and NF-*κ*B (Figure 7(b): Appendix J). Therefore, at the level of functional interaction of receptor-related proteins in the lipid rafts, it is clear that these membrane microdomains are a strong functional locus of this degenerative disease. Reinforcing this AD-relevant microcosm effect in the lipid rafts we analyzed, using latent semantic indexing (LSI) interrogation of these receptor-specific datasets (Table 2—control; Appendix I-3xTgAD), the Alzheimer's disease correlation of these receptor-specific proteins. In Figure 8, we demonstrate that almost twice as many proteins in the 3xTgAD dataset (21) explicitly correlated with the interrogation term Alzheimer's disease compared to the proteins in the control dataset (11). The degree of correlation of the interrogation term (Alzheimer's) to each specific protein is indicated by the LSI score. In Figure 8(b) the cumulated LSI score for the 3xTgAD Alzheimer's-related proteins was 2.92 while for the control proteins only 1.21 (Figure 8(b)), demonstrating that a much stronger correlation of receptor-associated proteins existed for the proteins in 3xTgAD rafts, compared to control. Upon comparison of the phylogenetic relationships of the receptor-specific proteins that form the most likely functional networks (from Figure 7(b)), it is clear that for the control-set proteins only three of these in this network are highly correlated to reports of Alzheimer's disease (27.2%: Figure 8(c)) while the most coherent interaction network of 3xTgAD raft proteins possessed a much higher percentage of Alzheimer's disease-related proteins (62%: Figure 8(d)). Therefore, this suggests that one of the important pathological loci of AD could be the disruption of interactivity of receptor-associated proteins in the lipid raft microdomains and that such a finding can be uncovered in a completely un-biased informatic format from extremely large and difficult to interpret datasets.

## 5. Discussion

Multiple informatic techniques were employed to investigate and elucidate the nature of functional protein interactions that occur differentially in the lipid raft microdomains of control versus 3xTgAD Alzheimer's disease mice. Cognitively impaired male 3xTgAD mice showed profound differences in the protein constituents of lipid raft extracts compared to age-matched control mice, that is, only 17% of raft proteins were similar between the two groups of mice. Our microdomain proteomic approach was able to specifically assist in the identification of altered proteins that are highly characteristic of AD-related raft pathophysiology, for example, the Src-family tyrosine kinase Fyn (Figure 3(h)) [19, 20].

Currently, biological scientists are often faced with complex biological issues concerning the interpretation of their results due to the development of facile mass data acquisition technologies, that is, extracting relevant and illuminating information from large datasets is often extremely challenging. However, with the application of multiple, sequential un-biased informatic processes, we were able to identify specific lipid raft involvement in altered pathways that can control multiple degenerative mechanisms, for example, attenuation of the Wnt/*β*-catenin signaling pathway, profound reduction of the important Nrf2 stress response pathway (Figure 4(d)), the loss of neurogenesis association, and the important involvement of synaptic GPCR-systems (Figure 6) in the 3xTgAD mice [36–38]. 

Elucidation of the crucial signaling relationships in this degenerative disorder, and the identity of the proteins that mediate them, could lead to the more rational development of novel therapeutics for Alzheimer's disease. Using our informatic receptor-targeted approach, we were also able to reinforce the validity of our discovery process by also identifying the importance of energy-related insulin/insulin-like growth factor (IGF) signaling in AD (Figures 3(j), 3(n), 5, and 8(d)) that has recently become more widely appreciated by other researchers [39–42]. In addition to IGF receptor activity, we noted a potential implication of the presynaptic latrophilins (lphn) in AD pathophysiology (Figure 8(d)). These unique receptors form the high affinity target of *α*-latrotoxin and may be able to integrate presynaptic calcium regulation with an ability to potentially physically interact with the postsynaptic neuron [43]. Another receptor signaling system that our analysis revealed to possess a potential role in AD is the Sonic Hedgehog (Shh) systems (Figures 5 and 8(d)). With a specific correlation to AD pathophysiology, it has been demonstrated that Shh can act in a synergistic manner with nerve growth factor to act on central nervous system cholinergic neurons [44], and cholinergic insufficiency has been strongly associated with cognitive decline and Alzheimer's-related pathophysiology [45].

## 6. Conclusions

The combined use of broad range proteomic analysis, with sequential multidimensional informatic analysis enables the determination of important correlations between pathophysiology and functional protein differences. Using discrete proteomes, that is, lipid raft extracts, versus whole-cell/tissue proteomes, facilitates an improved ability to understand the complex interactivity between transmembrane receptor protein systems in a disease setting. The appreciation and clustering of protein datasets into coherent groups greatly increases our capacity to focus upon potentially important therapeutic target networks. The knowledge gained of protein activity networks may greatly assist in the future development of network-targeting therapeutics that possess a multidimensional efficacy at several interacting proteins.

## Supplementary Material

Supplementary Table 1: the table illustrates the top 50 most upregulated and top 50 most downregulated (*italics*) proteins (commonly identified in 3xTgAD and control WT) in 3xTgAD cortical raft extracts compared to WT.
Ranking was based on relative expression ratios generated by spectral counting for 3xTgAD:WT proteins that displayed a >95% identity confidence.
Proteins ranked 1-50 for 3xTgAD>WT (increased in 3xTgAD compared to WT) represent those with expression ratios (3xTgAD:WT) greater than unity while proteins ranked 1-50 for WT>3xTgAD (decreased in 3xTgAD compared to WT: *italics*) represents those with expression ratios (3xTgAD:WT) less than unity. In each case the most up or downregulated proteins are ranked 1.

## Figures and Tables

**Table 1:** *Receptor-specific protein list from lipid raft extracts from control animals*. Primary protein lists of extracted lipid raft proteins were filtered for a specific receptor profile using IPA v. 8.5.

Symbol	Protein definition	Location	Protein type
Cd4	CD4 molecule	Plasma membrane	Transmembrane receptor
Celsr2	Cadherin, EGF LAG seven-pass G-type receptor 2	Plasma membrane	G-protein coupled receptor
Celsr3	Cadherin, EGF LAG seven-pass G-type receptor 3	Plasma membrane	G-protein coupled receptor
Chrm5	Cholinergic receptor, muscarinic 5	Plasma membrane	G-protein coupled receptor
Chrnb3	Cholinergic receptor, nicotinic, beta 3	Plasma membrane	Transmembrane receptor
Chrnb4	Cholinergic receptor, nicotinic, beta 4	Plasma membrane	Transmembrane receptor
Chrng	Cholinergic receptor, nicotinic, gamma	Plasma membrane	Transmembrane receptor
Cldn4	Claudin 4	Plasma membrane	Transmembrane receptor
Clecsf6	C-type lectin domain family 4, member A	Plasma membrane	Transmembrane receptor
Cnr1	Cannabinoid receptor 1 (brain)	Plasma membrane	G-protein coupled receptor
Cntfr	Ciliary neurotrophic factor receptor	Plasma membrane	Transmembrane receptor
Cxadr	Coxsackie virus and adenovirus receptor	Plasma membrane	Transmembrane receptor
Dag1	Dystroglycan 1 (dystrophin-associated glycoprotein 1)	Plasma membrane	Transmembrane receptor
Ednrb	Endothelin receptor type B	Plasma membrane	G-protein coupled receptor
Fshr	Follicle stimulating hormone receptor	Plasma membrane	G-protein coupled receptor
Fzd9	Frizzled homolog 9 (Drosophila)	Plasma membrane	G-protein coupled receptor
Gabbr1	Gamma-aminobutyric acid (GABA) B receptor, 1	Plasma membrane	G-protein coupled receptor
Gabrr3	Gamma-aminobutyric acid (GABA) receptor, rho 3	Plasma membrane	Transmembrane receptor
Gcgr	Glucagon receptor	Plasma membrane	G-protein coupled receptor
Gfra1	GDNF family receptor alpha 1	Plasma membrane	Transmembrane receptor
Gfra3	GDNF family receptor alpha 3	Plasma membrane	Transmembrane receptor
Gpr116	G protein-coupled receptor 116	Plasma membrane	G-protein coupled receptor
Gpr141	G protein-coupled receptor 141	Plasma membrane	G-protein coupled receptor
Gprc6a	G protein-coupled receptor, family C, group 6, member A	Plasma membrane	G-protein coupled receptor
Grm4	Glutamate receptor, metabotropic 4	Plasma membrane	G-protein coupled receptor
Grm5	Glutamate receptor, metabotropic 5	Plasma membrane	G-protein coupled receptor
Cbp	Opsin 1 (cone pigments), long-wave-sensitive	Plasma membrane	G-protein coupled receptor
Oprd1	Opioid receptor, delta 1	Plasma membrane	G-protein coupled receptor
Osmr	Oncostatin M receptor	Plasma membrane	Transmembrane receptor
Prom2	Prominin 2	Plasma membrane	Transmembrane receptor
Prph2	Peripherin 2 (retinal degeneration, slow)	Plasma membrane	Transmembrane receptor
Ptger2	Prostaglandin E receptor 2 (subtype EP2), 53kda	Plasma membrane	G-protein coupled receptor
Pthr2	Parathyroid hormone 2 receptor	Plasma membrane	G-protein coupled receptor
Sfrp4	Secreted frizzled-related protein 4	Plasma membrane	Transmembrane receptor
Smo	Smoothened homolog (Drosophila)	Plasma membrane	G-protein coupled receptor
Sstr2	Somatostatin receptor 2	Plasma membrane	G-protein coupled receptor
Tacr1	Tachykinin receptor 1	Plasma membrane	G-protein coupled receptor
Tas2r41	Taste receptor, type 2, member 41	Plasma membrane	G-protein coupled receptor
Thbd	Thrombomodulin	Plasma membrane	Transmembrane receptor
Tlr3	Toll-like receptor 3	Plasma membrane	Transmembrane receptor
Tlr4	Toll-like receptor 4	Plasma membrane	Transmembrane receptor
Tlr5	Toll-like receptor 5	Plasma membrane	Transmembrane receptor
Tlr6	Toll-like receptor 6	Plasma membrane	Transmembrane receptor
Tlr9	Toll-like receptor 9	Plasma membrane	Transmembrane receptor
Tshr	Thyroid stimulating hormone receptor	Plasma membrane	G-protein coupled receptor
Unc5b	Unc-5 homolog B (C. Elegans)	Plasma membrane	Transmembrane receptor
Vipr2	Vasoactive intestinal peptide receptor 2	Plasma membrane	G-protein coupled receptor

**Table 2:** *Receptor-specific protein list from lipid raft extracts from 3xTgAD animals*. Primary protein lists of extracted lipid raft proteins were filtered for a specific receptor profile using IPA v. 8.5.

Symbol	Protein definition	Location	Protein type
Adcyap1r1	Adenylate cyclase activating polypeptide 1 (pituitary) receptor type I	Plasma membrane	G-protein coupled receptor
Cd36	CD36 molecule (thrombospondin receptor)	Plasma membrane	Transmembrane receptor
Cd86	CD86 molecule	Plasma membrane	Transmembrane receptor
Cnr1	Cannabinoid receptor 1 (brain)	Plasma membrane	G-protein coupled receptor
Cxadr	Coxsackie virus and adenovirus receptor	Plasma membrane	Transmembrane receptor
Gabbr1	Gamma-aminobutyric acid (GABA) B receptor, 1	Plasma membrane	G-protein coupled receptor
Gpr1	G protein-coupled receptor 1	Plasma membrane	G-protein coupled receptor
Gpr141	G protein-coupled receptor 141	Plasma membrane	G-protein coupled receptor
Gpr56	G protein-coupled receptor 56	Plasma membrane	G-protein coupled receptor
Htr2c	5-hydroxytryptamine (serotonin) receptor 2C	Plasma membrane	G-protein coupled receptor
Htr4	5-hydroxytryptamine (serotonin) receptor 4	Plasma membrane	G-protein coupled receptor
Igf1r	Insulin-like growth factor 1 receptor	Plasma membrane	Transmembrane receptor
Igf2r	Insulin-like growth factor 2 receptor	Plasma membrane	Transmembrane receptor
Il1r2	Interleukin 1 receptor, type II	Plasma membrane	Transmembrane receptor
Il1rapl1	Interleukin 1 receptor accessory protein-like 1	Plasma membrane	Transmembrane receptor
Lifr	Leukemia inhibitory factor receptor alpha	Plasma membrane	Transmembrane receptor
Lphn1	Latrophilin 1	Plasma membrane	G-protein coupled receptor
Lphn2	Latrophilin 2	Plasma membrane	G-protein coupled receptor
Lrpap1	Low density lipoprotein receptor-related protein associated protein 1	Plasma membrane	Transmembrane receptor
Npr2	Natriuretic peptide receptor B/guanylate cyclase B	Plasma membrane	G-protein coupled receptor
Npy5r	Neuropeptide Y receptor Y5	Plasma membrane	G-protein coupled receptor
Omg	Oligodendrocyte myelin glycoprotein	Plasma membrane	G-protein coupled receptor
Cbp	Opsin 1 (cone pigments), long-wave-sensitive	Plasma membrane	G-protein coupled receptor
Ptch1	Patched homolog 1 (Drosophila)	Plasma membrane	Transmembrane receptor
Pthr2	Parathyroid hormone 2 receptor	Plasma membrane	G-protein coupled receptor
Robo1	Roundabout, axon guidance receptor, homolog 1 (Drosophila)	Plasma membrane	Transmembrane receptor
Lgr7	Relaxin/insulin-like family peptide receptor 1	Plasma membrane	G-protein coupled receptor
Tlr5	Toll-like receptor 5	Plasma membrane	Transmembrane receptor
Tnfrsf14	Tumor necrosis factor receptor superfamily, member 14	Plasma membrane	Transmembrane receptor
Tshr	Thyroid stimulating hormone receptor	Plasma membrane	G-protein coupled receptor

## Appendices

### A.

Lipid raft-derived extracted proteins from control mouse 16-month-old cortical tissue. Specific proteins isolated from control mouse cortex were identified with multidimensional protein identification technology (MudPIT) LC MS/MS. Each protein was identified in at least two out of three experimental replicate animals and from at least two isolated peptides per protein. The following are the protein symbol and its corresponding definitions.

Atm: ataxia telangiectasia mutated
Bcat1: branched chain aminotransferase 1, cytosolic
Bcl11a: B-cell CLL/lymphoma 11A (zinc finger protein)
Bnip1: BCL2/adenovirus E1B 19 kDa interacting protein 1
Brd2: bromodomain containing 2
Bteb1: Kruppel-like factor 9
Bzrap1: benzodiazepine receptor (peripheral) associated protein 1
C3: complement component 3
C5: complement component 5
Cacna1a: calcium channel, voltage-dependent, P/Q type, alpha 1A subunit
Cacna1c: calcium channel, voltage-dependent, L type, alpha 1C subunit
Cacna2d2: calcium channel, voltage-dependent, alpha 2/delta subunit 2
Cadps: Ca++-dependent secretion activator
Calb1: calbindin 1, 28 kDa
Calb2: calbindin 2
Calm2: calmodulin 2 (phosphorylase kinase, delta)
Camkk1: calcium/calmodulin-dependent protein kinase kinase 1, alpha
Camkk2: calcium/calmodulin-dependent protein kinase kinase 2, beta
Cap1: CAP, adenylate cyclase-associated protein 1 (yeast)
Cast: calpastatin
Cat: catalase
Catsper2: cation channel, sperm associated 2
Cav1: caveolin 1, caveolae protein, 22 kDa
Cblb: Cas-Br-M (murine) ecotropic retroviral transforming sequence b
Cbp: opsin 1 (cone pigments), long-wave-sensitive
Cbr1: carbonyl reductase 1
Cbwd1: COBW domain containing 1
Cbx5: chromobox homolog 5 (HP1 alpha homolog, Drosophila)
Ccbl1: cysteine conjugate-beta lyase, cytoplasmic
Ccin: calicin
Ccl28: chemokine (C-C motif) ligand 28
Ccl4: chemokine (C-C motif) ligand 4
ccnb1: cyclin B1
Ccnc: cyclin C
Cct2: chaperonin containing TCP1, subunit 2 (beta)
Cct3: chaperonin containing TCP1, subunit 3 (gamma)
Cct4: chaperonin containing TCP1, subunit 4 (delta)
Cct5: chaperonin containing TCP1, subunit 5 (epsilon)
Cct6a: chaperonin containing TCP1, subunit 6A (zeta 1)
Cd1: ribosomal protein L5
Cd151: CD151 molecule (Raph blood group)
Cd200: CD200 molecule
Cd276: CD276 molecule
Cd2ap: CD2-associated protein
Cd4: CD4 molecule
Cdc10: septin 7
Cdc25b: cell division cycle 25 homolog B (S. pombe)
Cdc42: cell division cycle 42 (GTP binding protein, 25 kDa)
Cdc42bpa: CDC42 binding protein kinase alpha (DMPK-like)
Cdc5l: CDC5 cell division cycle 5-like (S. pombe)
Cdh1: cadherin 1, type 1, E-cadherin (epithelial)
Cdh10: cadherin 10, type 2 (T2-cadherin)
Cdh2: cadherin 2, type 1, N-cadherin (neuronal)
Cdh4: cadherin 4, type 1, R-cadherin (retinal)
Cdk2: cyclin-dependent kinase 2
Cdk5rap2: CDK5 regulatory subunit associated protein 2
Cdk5rap3: CDK5 regulatory subunit associated protein 3
Cdk6: cyclin-dependent kinase 6
Cdk7: cyclin-dependent kinase 7
Cdkl2: cyclin-dependent kinase-like 2 (CDC2-related kinase)
Cdkl3: cyclin-dependent kinase-like 3
Cdkn1b: cyclin-dependent kinase inhibitor 1B (p27, Kip1)
Cds1: CDP-diacylglycerol synthase (phosphatidate cytidylyltransferase) 1
Cdv1: intraflagellar transport 81 homolog (Chlamydomonas)
Cebpe: CCAAT/enhancer binding protein (C/EBP), epsilon
Cebpz: CCAAT/enhancer binding protein (C/EBP), zeta
Celsr2: cadherin, EGF LAG seven-pass G-type receptor 2 (flamingo homolog, Drosophila)
Celsr3: cadherin, EGF LAG seven-pass G-type receptor 3 (flamingo homolog, Drosophila)
Cend1: cell cycle exit and neuronal differentiation 1
Cenpf: centromere protein F, 350/400ka (mitosin)
Centa1: ArfGAP with dual PH domains 1
Centg1: ArfGAP with GTPase domain, ankyrin repeat and PH domain 2
Cep350: centrosomal protein 350 kDa
Ces1: carboxylesterase 1 (monocyte/macrophage serine esterase 1)
Ces3: carboxylesterase 3
Cetn2: centrin, EF-hand protein, 2
Cetn3: centrin, EF-hand protein, 3 (CDC31 homolog, yeast)
Cfh: complement factor H
Cfl1: cofilin 1 (nonmuscle)
Chd3: chromodomain helicase DNA binding protein 3
Chd4: chromodomain helicase DNA binding protein 4
Chga: chromogranin A (parathyroid secretory protein 1)
Chl1: cell adhesion molecule with homology to L1CAM (close homolog of L1)
Chmp1a: chromatin modifying protein 1A
Chn2: chimerin (chimaerin) 2
Chrm5: cholinergic receptor, muscarinic 5
Chrnb3: cholinergic receptor, nicotinic, beta 3
Chrnb4: cholinergic receptor, nicotinic, beta 4
Chrng: cholinergic receptor, nicotinic, gamma
Chst7: carbohydrate (N-acetylglucosamine 6-O) sulfotransferase 7
Chx10: visual system homeobox 2
Cirbp: cold inducible RNA binding protein
Cit: citron (rho-interacting, serine/threonine kinase 21)
Cktsf1b1: gremlin 1, cysteine knot superfamily, homolog (Xenopus laevis)
Clasp2: cytoplasmic linker associated protein 2
Clcc1: chloride channel CLIC-like 1
Clcn7: chloride channel 7
Cldn1: claudin 1
Cldn19: claudin 19
Cldn4: claudin 4
CLEC10A: C-type lectin domain family 10, member A
Clecsf6: C-type lectin domain family 4, member A
Clic4: chloride intracellular channel 4
CLIP3: CAP-GLY domain containing linker protein 3
Clpb: ClpB caseinolytic peptidase B homolog (E. coli)
Clstn1: calsyntenin 1
Cltb: clathrin, light chain (Lcb)
Cndp2: CNDP dipeptidase 2 (metallopeptidase M20 family)
Cnga1: cyclic nucleotide gated channel alpha 1
Cnga4: cyclic nucleotide gated channel alpha 4
Cnksr2: connector enhancer of kinase suppressor of Ras 2
Cnot6: CCR4-NOT transcription complex, subunit 6
Cnp: 2^'^,3^'^-cyclic nucleotide 3^'^ phosphodiesterase
Cnr1: cannabinoid receptor 1 (brain)
Cntfr: ciliary neurotrophic factor receptor
Cntn1: contactin 1
Cog3: component of oligomeric golgi complex 3
Colm: gliomedin
Copa: coatomer protein complex, subunit alpha
Copb1: coatomer protein complex, subunit beta 1
COPB2: coatomer protein complex, subunit beta 2 (beta prime)
Cops4: COP9 constitutive photomorphogenic homolog subunit 4 (Arabidopsis)
Cops8: COP9 constitutive photomorphogenic homolog subunit 8 (Arabidopsis)
Coro1a: coronin, actin binding protein, 1A
Coro7: coronin 7
Cp: ceruloplasmin (ferroxidase)
Cpe: carboxypeptidase E
Cplx1: complexin 1
Cpt1a: carnitine palmitoyltransferase 1A (liver)
Cpt2: carnitine palmitoyltransferase 2
Crabp2: cellular retinoic acid binding protein 2
Crhbp: corticotropin releasing hormone binding protein
Crip2: cysteine-rich protein 2
Crk: v-crk sarcoma virus CT10 oncogene homolog (avian)
Crkl: v-crk sarcoma virus CT10 oncogene homolog (avian)-like
Crmp1: collapsin response mediator protein 1
Crnkl1: crooked neck pre-mRNA splicing factor-like 1 (Drosophila)
Crp: C-reactive protein, pentraxin-related
Cry1: cryptochrome 1 (photolyase-like)
Crygc: crystallin, gamma C
Crygs: crystallin, gamma S
Crym: crystallin, mu
Cs: citrate synthase
Csf1: colony stimulating factor 1 (macrophage)
Csnk1e: casein kinase 1, epsilon
Csnk1g2: casein kinase 1, gamma 2
Csnk2a1: casein kinase 2, alpha 1 polypeptide
Csnk2b: casein kinase 2, beta polypeptide
Cspg2: versican
Cspg3: neurocan
Cspg5: chondroitin sulfate proteoglycan 5 (neuroglycan C)
Cspg6: structural maintenance of chromosomes 3
Cst6: cystatin E/M
Cstb: cystatin B (stefin B)
Ctf1: cardiotrophin 1
Cthrc1: collagen triple helix repeat containing 1
Ctla4: cytotoxic T-lymphocyte-associated protein 4
Ctnnb1: catenin (cadherin-associated protein), beta 1, 88 kDa
Ctnnd2: catenin (cadherin-associated protein), delta 2 (neural plakophilin-related arm-repeat protein)
Ctrb: chymotrypsinogen B1
Ctrl: chymotrypsin-like
Ctsd: cathepsin D
Cttn: cortactin
Cttnbp2: cortactin binding protein 2
Cul1: cullin 1
Cul5: cullin 5
Cuta: cutA divalent cation tolerance homolog (E. coli)
Cutl1: cut-like homeobox 1
CX3CL1: chemokine (C-X3-C motif) ligand 1
Cxadr: coxsackie virus and adenovirus receptor
Cxcl2: chemokine (C-X-C motif) ligand 2
Cyb5: cytochrome b5 type A (microsomal)
Cyb5b: cytochrome b5 type B (outer mitochondrial membrane)
Cyb5r1: cytochrome b5 reductase 1
Cybb: cytochrome b-245, beta polypeptide
Cyln2: CAP-GLY domain containing linker protein 2
Cyp2s1: cytochrome P450, family 2, subfamily S, polypeptide 1
Cyr61: cysteine-rich, angiogenic inducer, 61
Dab2: disabled homolog 2, mitogen-responsive phosphoprotein (Drosophila)
Dab2ip: DAB2 interacting protein
Dad1: defender against cell death 1
Dag1: dystroglycan 1 (dystrophin-associated glycoprotein 1)
Dap: death-associated protein
Dapk3: death-associated protein kinase 3
Dbc1: deleted in bladder cancer 1
DBH: dopamine beta-hydroxylase (dopamine beta-monooxygenase)
Dbn1: drebrin 1
Dbndd2: dysbindin (dystrobrevin binding protein 1) domain containing 2
Dbnl: drebrin-like
Dcd: dermcidin
Dci: dodecenoyl-Coenzyme A delta isomerase (3,2 trans-enoyl-Coenzyme A isomerase)
Dclk2: doublecortin-like kinase 2
Dclre1c: DNA cross-link repair 1C (PSO2 homolog, S. cerevisiae)
Dctn2: dynactin 2 (p50)
Ddah2: dimethylarginine dimethylaminohydrolase 2
Ddb1: damage-specific DNA binding protein 1, 127 kDa
Ddn: dendrin
Ddost: dolichyl-diphosphooligosaccharide-protein glycosyltransferase
Ddr2: discoidin domain receptor tyrosine kinase 2
Ddt: D-dopachrome tautomerase
Ddx1: DEAD (Asp-Glu-Ala-Asp) box polypeptide 1
Ddx17: DEAD (Asp-Glu-Ala-Asp) box polypeptide 17
Ddx47: DEAD (Asp-Glu-Ala-Asp) box polypeptide 47
Ddx5: DEAD (Asp-Glu-Ala-Asp) box polypeptide 5
Decr1: 2,4-dienoyl CoA reductase 1, mitochondrial
Des: desmin
Dgcr14: DiGeorge syndrome critical region gene 14
Dgkb: diacylglycerol kinase, beta 90 kDa
Dgkg: diacylglycerol kinase, gamma 90 kDa
Dhcr7: 7-dehydrocholesterol reductase
Dhodh: dihydroorotate dehydrogenase
Dhrs8: hydroxysteroid (17-beta) dehydrogenase 11
Dhx16: DEAH (Asp-Glu-Ala-His) box polypeptide 16
Dhx30: DEAH (Asp-Glu-Ala-His) box polypeptide 30
Dia1: cytochrome b5 reductase 3
Dio1: deiodinase, iodothyronine, type I
Disc1: disrupted in schizophrenia 1
Dkc1: dyskeratosis congenita 1, dyskerin
Dlat: dihydrolipoamide S-acetyltransferase
Dlc1: deleted in liver cancer 1
Dld: dihydrolipoamide dehydrogenase
Dlgap1: discs, large (Drosophila) homolog-associated protein 1
Dlgap2: discs, large (Drosophila) homolog-associated protein 2
Dlgap4: discs, large (Drosophila) homolog-associated protein 4
Dll1: delta-like 1 (Drosophila)
Dll3: delta-like 3 (Drosophila)
Dlx5: distal-less homeobox 5
Dmd: dystrophin
Dmn: synemin, intermediate filament protein
Dmrt1: doublesex and mab-3 related transcription factor 1
Dnah6: dynein, axonemal, heavy chain 6
Dnaja2: DnaJ (Hsp40) homolog, subfamily A, member 2
Dnch1: dynein, cytoplasmic 1, heavy chain 1
Dncl2a: dynein, light chain, roadblock-type 1
Dntt: deoxynucleotidyltransferase, terminal
Dock9: dedicator of cytokinesis 9
Dpp3: dipeptidyl-peptidase 3
Dpyd: dihydropyrimidine dehydrogenase
Dpysl3: dihydropyrimidinase-like 3
Dpysl4: dihydropyrimidinase-like 4
Drd1ip: calcyon neuron-specific vesicular protein
Drg1: developmentally regulated GTP binding protein 1
Drpla: atrophin 1
Dscam: Down syndrome cell adhesion molecule
Dscr1l1: regulator of calcineurin 2
Dtnb: dystrobrevin, beta
Duox1: dual oxidase 1
Dusp12: dual specificity phosphatase 12
Dusp2: dual specificity phosphatase 2
Dvl1: dishevelled, dsh homolog 1 (Drosophila)
Dync1h1: dynein, cytoplasmic 1, heavy chain 1
Dync1li2: dynein, cytoplasmic 1, light intermediate chain 2
Dyx1c1: dyslexia susceptibility 1 candidate 1
Eaf2: ELL associated factor 2
Ecel1: endothelin converting enzyme-like 1
Echdc1: enoyl Coenzyme A hydratase domain containing 1
Ecm1: extracellular matrix protein 1
Ecm2: extracellular matrix protein 2, female organ and adipocyte specific
Edf1: endothelial differentiation-related factor 1
Ednrb: endothelin receptor type B
Eef2: eukaryotic translation elongation factor 2
Efcbp1: N-terminal EF-hand calcium binding protein 1
Efemp2: EGF-containing fibulin-like extracellular matrix protein 2
Efnb1: ephrin-B1
Egf: epidermal growth factor (beta-urogastrone)
Egfl3: multiple EGF-like-domains 6
Egfr: epidermal growth factor receptor (erythroblastic leukemia viral (v-erb-b) oncogene homolog, avian)
Ehd2: EH-domain containing 2
Ehd4: EH-domain containing 4
Eif2c2: eukaryotic translation initiation factor 2C, 2
Eif5b: eukaryotic translation initiation factor 5B
Elac2: elaC homolog 2 (E. coli)
Elavl3: ELAV (embryonic lethal, abnormal vision, Drosophila)-like 3 (Hu antigen C)
ELK1: ELK1, member of ETS oncogene family
Elmo1: engulfment and cell motility 1
Emb: embigin homolog (mouse)
Emd: emerin
Eml5: echinoderm microtubule associated protein like 5
Enah: enabled homolog (Drosophila)
Enc1: ectodermal-neural cortex (with BTB-like domain)
Eno2: enolase 2 (gamma, neuronal)
Eno3: enolase 3 (beta, muscle)
Enpp2: ectonucleotide pyrophosphatase/phosphodiesterase 2
Enpp3: ectonucleotide pyrophosphatase/phosphodiesterase 3
Enpp5: ectonucleotide pyrophosphatase/phosphodiesterase 5 (putative function)
Ensa: endosulfine alpha
Entpd2: ectonucleoside triphosphate diphosphohydrolase 2
Entpd8: ectonucleoside triphosphate diphosphohydrolase 8
Epha3: EPH receptor A3
Epha5: EPH receptor A5
Epha7: EPH receptor A7
Ephb3: EPH receptor B3
Ephx1: epoxide hydrolase 1, microsomal (xenobiotic)
Epn1: epsin 1
Eprs: glutamyl-prolyl-tRNA synthetase
Eps8l1: EPS8-like 1
Erap1: endoplasmic reticulum aminopeptidase 1
Erbb3: v-erb-b2 erythroblastic leukemia viral oncogene homolog 3 (avian)
Erbb4: v-erb-a erythroblastic leukemia viral oncogene homolog 4 (avian)
Ercc5: excision repair cross-complementing rodent repair deficiency, complementation group 5
Erp29: endoplasmic reticulum protein 29
Esd: esterase D/formylglutathione hydrolase
Esr2: estrogen receptor 2 (ER beta)
Etfb: electron-transfer-flavoprotein, beta polypeptide
Ets1: v-ets erythroblastosis virus E26 oncogene homolog 1 (avian)
Evl: Enah/Vasp-like
Exoc5: exocyst complex component 5
Exoc7: exocyst complex component 7
Ezr: ezrin
Fabp1: fatty acid binding protein 1, liver
Fabp2: fatty acid binding protein 2, intestinal
Fabp3: fatty acid binding protein 3, muscle and heart (mammary-derived growth inhibitor)
Fabp4: fatty acid binding protein 4, adipocyte
Fabp7: fatty acid binding protein 7, brain
Fads1: fatty acid desaturase 1
Fads2: fatty acid desaturase 2
Faim: Fas apoptotic inhibitory molecule
Fancd2: Fanconi anemia, complementation group D2
Faslg: Fas ligand (TNF superfamily, member 6)
Fasn: fatty acid synthase
Fat: FAT tumor suppressor homolog 1 (Drosophila)
Fat3: FAT tumor suppressor homolog 3 (Drosophila)
Fbn1: fibrillin 1
Fbn2: fibrillin 2
Fbxw11: F-box and WD repeat domain containing 11
Fetub: fetuin B
FGA: fibrinogen alpha chain
Fgb: fibrinogen beta chain
Fgf18: fibroblast growth factor 18
Fgf23: fibroblast growth factor 23
Fgfr1: fibroblast growth factor receptor 1
Fgfr1op2: FGFR1 oncogene partner 2
Fgfr2: fibroblast growth factor receptor 2
Fgfr3: fibroblast growth factor receptor 3
Fhl1: four and a half LIM domains 1
Fignl1: fidgetin-like 1
Fip1l1: FIP1 like 1 (S. cerevisiae)
Fkbp14: FK506 binding protein 14, 22 kDa
Fkbp1b: FK506 binding protein 1B, 12.6 kDa
Fkbp5: FK506 binding protein 5
Flg: filaggrin
Flii: flightless I homolog (Drosophila)
Fmo3: flavin containing monooxygenase 3
Fnta: farnesyltransferase, CAAX box, alpha
Fos: FBJ murine osteosarcoma viral oncogene homolog
Fpgt: fucose-1-phosphate guanylyltransferase
Freq: frequenin homolog (Drosophila)
Fshr: follicle stimulating hormone receptor
Fthfd: aldehyde dehydrogenase 1 family, member L1
Fuca1: fucosidase, alpha-L-1, tissue
fut10: fucosyltransferase 10 (alpha (1,3) fucosyltransferase)
Fxc1: fracture callus 1 homolog (rat)
Fxyd6: FXYD domain containing ion transport regulator 6
Fzd9: frizzled homolog 9 (Drosophila)
Gaa: glucosidase, alpha; acid
Gabarap: GABA(A) receptor-associated protein
Gabarapl2: GABA(A) receptor-associated protein-like 2
Gabbr1: gamma-aminobutyric acid (GABA) B receptor, 1
Gabra3: gamma-aminobutyric acid (GABA) A receptor, alpha 3
Gabrg2: gamma-aminobutyric acid (GABA) A receptor, gamma 2
Gabrr3: gamma-aminobutyric acid (GABA) receptor, rho 3
Gadd45a: growth arrest and DNA-damage-inducible, alpha
Galc: galactosylceramidase
Galk1: galactokinase 1
Galt: galactose-1-phosphate uridylyltransferase
Gapdh: glyceraldehyde-3-phosphate dehydrogenase
Gars: glycyl-tRNA synthetase
Gas6: growth arrest-specific 6
Gas7: growth arrest-specific 7
Gata2: GATA binding protein 2
Gata3: GATA binding protein 3
GC: group-specific component (vitamin D binding protein)
Gc: group-specific component (vitamin D binding protein)
Gcgr: glucagon receptor
Gckr: glucokinase (hexokinase 4) regulator
Gclc: glutamate-cysteine ligase, catalytic subunit
Gcnt3: glucosaminyl (N-acetyl) transferase 3, mucin type
Gdap2: ganglioside induced differentiation associated protein 2
Gdi1: GDP dissociation inhibitor 1
Gdi2: GDP dissociation inhibitor 2
Gfap: glial fibrillary acidic protein
Gfpt2: glutamine-fructose-6-phosphate transaminase 2
Gfra1: GDNF family receptor alpha 1
Gfra3: GDNF family receptor alpha 3
Ggt1: gamma-glutamyltransferase 1
Ggtl3: gamma-glutamyltransferase 7
Ggtla1: gamma-glutamyltransferase 5
Ghitm: growth hormone inducible transmembrane protein
Gif: gastric intrinsic factor (vitamin B synthesis)
Gipc1: GIPC PDZ domain containing family, member 1
Git1: G protein-coupled receptor kinase interacting ArfGAP 1
Gja1: gap junction protein, alpha 1, 43 kDa
Gja10: gap junction protein, alpha 10, 62 kDa
Gja4: gap junction protein, alpha 4, 37 kDa
Gjb6: gap junction protein, beta 6, 30 kDa
Gla: galactosidase, alpha
Gldc: glycine dehydrogenase (decarboxylating)
Glg1: golgi apparatus protein 1
Gli: GLI family zinc finger 1
Glo1: glyoxalase I
Glra2: glycine receptor, alpha 2
Glrx2: glutaredoxin 2
Glud1: glutamate dehydrogenase 1
Glul: glutamate-ammonia ligase (glutamine synthetase)
Gm2a: GM2 ganglioside activator
Gmcl1: germ cell-less homolog 1 (Drosophila)
Gmfb: glia maturation factor, beta
Gmfg: glia maturation factor, gamma
Gmpr2: guanosine monophosphate reductase 2
Gng7: guanine nucleotide binding protein (G protein), gamma 7
Gnpat: glyceronephosphate O-acyltransferase
Gns: glucosamine (N-acetyl)-6-sulfatase
Golga2: golgi autoantigen, golgin subfamily a, 2
Golgb1: golgin B1, golgi integral membrane protein
Golph3: golgi phosphoprotein 3 (coat-protein)
Golph4: golgi integral membrane protein 4
Gosr2: golgi SNAP receptor complex member 2
Got1: glutamic-oxaloacetic transaminase 1, soluble (aspartate aminotransferase 1)
Got2: glutamic-oxaloacetic transaminase 2, mitochondrial (aspartate aminotransferase 2)
Gpam: glycerol-3-phosphate acyltransferase, mitochondrial
Gpc2: glypican 2
Gpi: glucose phosphate isomerase
Gpm6a: glycoprotein M6A
Gpr116: G protein-coupled receptor 116
Gpr141: G protein-coupled receptor 141
Gprc6a: G protein-coupled receptor, family C, group 6, member A
Gpsm1: G-protein signaling modulator 1 (AGS3-like, C. elegans)
Gpt: glutamic-pyruvate transaminase (alanine aminotransferase)
Gramd1a: GRAM domain containing 1A
Grasp: GRP1 (general receptor for phosphoinositides 1)-associated scaffold protein
Grem1: gremlin 1, cysteine knot superfamily, homolog (Xenopus laevis)
Gria1: glutamate receptor, ionotropic, AMPA 1
Grid2: glutamate receptor, ionotropic, delta 2
Grik1: glutamate receptor, ionotropic, kainate 1
Grik4: glutamate receptor, ionotropic, kainate 4
Grin2a: glutamate receptor, ionotropic, N-methyl D-aspartate 2A
Grin2b: glutamate receptor, ionotropic, N-methyl D-aspartate 2B
Grip1: glutamate receptor interacting protein 1
Gripap1: GRIP1 associated protein 1
Grk4: G protein-coupled receptor kinase 4
Grk5: G protein-coupled receptor kinase 5
Grk6: G protein-coupled receptor kinase 6
Grm4: glutamate receptor, metabotropic 4
Grm5: glutamate receptor, metabotropic 5
Grpel1: GrpE-like 1, mitochondrial (E. coli)
Gstk1: glutathione S-transferase kappa 1
Gstm2: glutathione S-transferase mu 2 (muscle)
Gsto1: glutathione S-transferase omega 1
Gtf2ird1: GTF2I repeat domain containing 1
Gtpbp4: GTP binding protein 4
Gucy1a2: guanylate cyclase 1, soluble, alpha 2
Gucy1a3: guanylate cyclase 1, soluble, alpha 3
Gucy1b2: guanylate cyclase 1, soluble, beta 2
Gucy2d: guanylate cyclase 2D, membrane (retina-specific)
Gulp1: GULP, engulfment adaptor PTB domain containing 1
Haao: 3-hydroxyanthranilate 3,4-dioxygenase
Itpka: inositol 1,4,5-trisphosphate 3-kinase A
Khdrbs2: KH domain containing, RNA binding, signal transduction associated 2
Kpnb1: karyopherin (importin) beta 1
Lamp2: lysosomal-associated membrane protein 2
Ncstn: nicastrin
Nedd4l: neural precursor cell expressed, developmentally down-regulated 4-like
Nip7: nuclear import 7 homolog (S. cerevisiae)
Nnat: neuronatin
Nos1: nitric oxide synthase 1 (neuronal)
Nox1: NADPH oxidase 1
Npepps: aminopeptidase puromycin sensitive
Nr1h3: nuclear receptor subfamily 1, group H, member 3
Nr1i2: nuclear receptor subfamily 1, group I, member 2
Nr6a1: nuclear receptor subfamily 6, group A, member 1
Nrcam: neuronal cell adhesion molecule
Nrd1: nardilysin (N-arginine dibasic convertase)
Ntn1: netrin 1
Nucb2: nucleobindin 2
Nudc: nuclear distribution gene C homolog (A. nidulans)
Nup35: nucleoporin 35 kDa
Nup54: nucleoporin 54 kDa
Nup98: nucleoporin 98 kDa
Oaz1: ornithine decarboxylase antizyme 1
Ocrl: oculocerebrorenal syndrome of Lowe
Oit3: oncoprotein induced transcript 3
Olfm2: olfactomedin 2
Opa1: optic atrophy 1 (autosomal dominant)
Oplah: 5-oxoprolinase (ATP-hydrolysing)
Oprd1: opioid receptor, delta 1
Orc4l: origin recognition complex, subunit 4-like (yeast)
Osmr: oncostatin M receptor
Oxr1: oxidation resistance 1
P2rx7: purinergic receptor P2X, ligand-gated ion channel, 7
P4hb: prolyl 4-hydroxylase, beta polypeptide
Pa2g4: proliferation-associated 2G4, 38 kDa
Pabpc4: poly(A) binding protein, cytoplasmic 4 (inducible form)
Pace4: proprotein convertase subtilisin/kexin type 6
Pacs1: phosphofurin acidic cluster sorting protein 1
Pafah1b2: platelet-activating factor acetylhydrolase, isoform Ib, subunit 2 (30 kDa)
Pafah1b3: platelet-activating factor acetylhydrolase, isoform Ib, subunit 3 (29 kDa)
Pak1: p21 protein (Cdc42/Rac)-activated kinase 1
Pak2: p21 protein (Cdc42/Rac)-activated kinase 2
Pak3: p21 protein (Cdc42/Rac)-activated kinase 3
Palm: paralemmin
Pamci: Ras association (RalGDS/AF-6) domain family (N-terminal) member 9
Panx1: pannexin 1
Panx2: pannexin 2
Pard3: par-3 partitioning defective 3 homolog (C. elegans)
Parg: poly (ADP-ribose) glycohydrolase
Park7: Parkinson disease (autosomal recessive, early onset) 7
Pawr: PRKC, apoptosis, WT1, regulator
Pax3: paired box 3
Pbp: phosphatidylethanolamine binding protein 1
Pc: pyruvate carboxylase
Pcca: propionyl Coenzyme A carboxylase, alpha polypeptide
Pcdh21: protocadherin 21
Pcdh7: protocadherin 7
Pcdha3: protocadherin alpha 3
Pcdhb1: protocadherin beta 1
Pck1: phosphoenolpyruvate carboxykinase 1 (soluble)
Pclo: piccolo (presynaptic cytomatrix protein)
Pcm1: pericentriolar material 1
Pcmt1: protein-L-isoaspartate (D-aspartate) O-methyltransferase
PCNT: pericentrin
Pcnx: pecanex homolog (Drosophila)
Pcnxl3: pecanex-like 3 (Drosophila)
Pcsk1: proprotein convertase subtilisin/kexin type 1
Pcsk1n: proprotein convertase subtilisin/kexin type 1 inhibitor
Pcsk5: proprotein convertase subtilisin/kexin type 5
Pcyox1: prenylcysteine oxidase 1
Pcyt1b: phosphate cytidylyltransferase 1, choline, beta
Pdap1: PDGFA associated protein 1
Pdc: phosducin
Pdcd2: programmed cell death 2
Pdcl: phosducin-like
Pde10a: phosphodiesterase 10A
Pde1a: phosphodiesterase 1A, calmodulin-dependent
Pde1c: phosphodiesterase 1C, calmodulin-dependent 70 kDa
Pde4a: phosphodiesterase 4A, cAMP-specific (phosphodiesterase E2 dunce homolog, Drosophila)
Pde4b: phosphodiesterase 4B, cAMP-specific (phosphodiesterase E4 dunce homolog, Drosophila)
Pde4d: phosphodiesterase 4D, cAMP-specific (phosphodiesterase E3 dunce homolog, Drosophila)
Pde5a: phosphodiesterase 5A, cGMP-specific
Pde7a: phosphodiesterase 7A
Pde7b: phosphodiesterase 7B
Pdgfra: platelet-derived growth factor receptor, alpha polypeptide
Pdhb: pyruvate dehydrogenase (lipoamide) beta
Pdia3: protein disulfide isomerase family A, member 3
Pdia4: protein disulfide isomerase family A, member 4
Pdk4: pyruvate dehydrogenase kinase, isozyme 4
Pdlim7: PDZ and LIM domain 7 (enigma)
Pds5b: PDS5, regulator of cohesion maintenance, homolog B (S. cerevisiae)
Pdzd3: PDZ domain containing 3
Pepd: peptidase D
Per3: period homolog 3 (Drosophila)
Pex14: peroxisomal biogenesis factor 14
Pfkl: phosphofructokinase, liver
Pfkm: phosphofructokinase, muscle
Pfkp: phosphofructokinase, platelet
Pgam1: phosphoglycerate mutase 1 (brain)
PGAP1: post-GPI attachment to proteins 1
Pgd: phosphogluconate dehydrogenase
Pgf: placental growth factor
Pgk1: phosphoglycerate kinase 1
Pgm1: phosphoglucomutase 1
Pgr: progesterone receptor
Pgrmc1: progesterone receptor membrane component 1
Phactr1: phosphatase and actin regulator 1
Phactr3: phosphatase and actin regulator 3
Phb: prohibitin
Phex: phosphate regulating endopeptidase homolog, X-linked
Phgdh: phosphoglycerate dehydrogenase
Phip: pleckstrin homology domain interacting protein
Phtf1: putative homeodomain transcription factor 1
Phyhipl: phytanoyl-CoA 2-hydroxylase interacting protein-like
Pi4ka: phosphatidylinositol 4-kinase, catalytic, alpha
Picalm: phosphatidylinositol binding clathrin assembly protein
Pigq: phosphatidylinositol glycan anchor biosynthesis, class Q
Pigs: phosphatidylinositol glycan anchor biosynthesis, class S
Pik3cg: phosphoinositide-3-kinase, catalytic, gamma polypeptide
Pip5k1a: phosphatidylinositol-4-phosphate 5-kinase, type I, alpha
Pip5k1c: phosphatidylinositol-4-phosphate 5-kinase, type I, gamma
Pip5k2a: phosphatidylinositol-5-phosphate 4-kinase, type II, alpha
Pip5k2c: phosphatidylinositol-5-phosphate 4-kinase, type II, gamma
Pipox: pipecolic acid oxidase
Pitpn: phosphatidylinositol transfer protein, alpha
Pkd1: polycystic kidney disease 1 (autosomal dominant)
Pkia: protein kinase (cAMP-dependent, catalytic) inhibitor alpha
Pkm2: pyruvate kinase, muscle
Pla2g4c: phospholipase A2, group IVC (cytosolic, calcium-independent)
Plaa: phospholipase A2-activating protein
Plau: plasminogen activator, urokinase
Plcb1: phospholipase C, beta 1 (phosphoinositide-specific)
Plcl1: phospholipase C-like 1
Pld3: phospholipase D family, member 3
Plekha4: pleckstrin homology domain containing, family A (phosphoinositide binding specific) member 4
Plekhe1: PH domain and leucine rich repeat protein phosphatase 1
Plekhm1: pleckstrin homology domain containing, family M (with RUN domain) member 1
Plg: plasminogen
Plk1: polo-like kinase 1 (Drosophila)
Plrg1: pleiotropic regulator 1 (PRL1 homolog, Arabidopsis)
Plunc: palate, lung and nasal epithelium associated
Plvap: plasmalemma vesicle associated protein
Plxnb2: plexin B2
Pmpca: peptidase (mitochondrial processing) alpha
Pmvk: phosphomevalonate kinase
Pnliprp2: pancreatic lipase-related protein 2
Pnma1: paraneoplastic antigen MA1
Pno1: partner of NOB1 homolog (S. cerevisiae)
Pnpla2: patatin-like phospholipase domain containing 2
Pofut1: protein O-fucosyltransferase 1
Pon2: paraoxonase 2
Pon3: paraoxonase 3
Pop7: processing of precursor 7, ribonuclease P/MRP subunit (S. cerevisiae)
Por: P450 (cytochrome) oxidoreductase
Pou2f2: POU class 2 homeobox 2
Ppfia3: protein tyrosine phosphatase, receptor type, f polypeptide (PTPRF), interacting protein, alpha 3
Ppgb: cathepsin A
Ppib: peptidylprolyl isomerase B (cyclophilin B)
Ppid: peptidylprolyl isomerase D
Ppil3: peptidylprolyl isomerase (cyclophilin)-like 3
Ppm2c: pyruvate dehydrogenase phosphatase catalytic subunit 1
Ppp1r10: protein phosphatase 1, regulatory (inhibitor) subunit 10
Ppp1r14b: protein phosphatase 1, regulatory (inhibitor) subunit 14B
Ppp1r14d: protein phosphatase 1, regulatory (inhibitor) subunit 14D
Ppp1r1a: protein phosphatase 1, regulatory (inhibitor) subunit 1A
Ppp1r7: protein phosphatase 1, regulatory (inhibitor) subunit 7
Ppp1r8: protein phosphatase 1, regulatory (inhibitor) subunit 8
Ppp1r9a: protein phosphatase 1, regulatory (inhibitor) subunit 9A
Ppp1r9b: protein phosphatase 1, regulatory (inhibitor) subunit 9B
Ppp2cb: protein phosphatase 2 (formerly 2A), catalytic subunit, beta isoform
Ppp2r1a: protein phosphatase 2 (formerly 2A), regulatory subunit A, alpha isoform
Ppp2r1b: protein phosphatase 2 (formerly 2A), regulatory subunit A, beta isoform
Ppp2r2a: protein phosphatase 2 (formerly 2A), regulatory subunit B, alpha isoform
PPP2R2B: protein phosphatase 2 (formerly 2A), regulatory subunit B, beta isoform
Ppp2r5e: protein phosphatase 2, regulatory subunit B', epsilon isoform
Ppp3cc: protein phosphatase 3 (formerly 2B), catalytic subunit, gamma isoform
Ppp5c: protein phosphatase 5, catalytic subunit
Prdm4: PR domain containing 4
Prdx1: peroxiredoxin 1
Prep: prolyl endopeptidase
Prf1: perforin 1 (pore forming protein)
Prkaa2: protein kinase, AMP-activated, alpha 2 catalytic subunit
Prkaca: protein kinase, cAMP-dependent, catalytic, alpha
prkar1a: protein kinase, cAMP-dependent, regulatory, type I, alpha (tissue specific extinguisher 1)
Prkar1a: protein kinase, cAMP-dependent, regulatory, type I, alpha (tissue specific extinguisher 1)
Prkar2a: protein kinase, cAMP-dependent, regulatory, type II, alpha
Prkar2b: protein kinase, cAMP-dependent, regulatory, type II, beta
Prkca: protein kinase C, alpha
Prkcb1: protein kinase C, beta
Prkci: protein kinase C, iota
Prkcm: protein kinase D1
PRKCQ: protein kinase C, theta
Prkcz: protein kinase C, zeta
Prkwnk1: WNK lysine deficient protein kinase 1
Prmt3: protein arginine methyltransferase 3
Prnp: prion protein
Prom2: prominin 2
Prph2: peripherin 2 (retinal degeneration, slow)
Prpsap1: phosphoribosyl pyrophosphate synthetase-associated protein 1
Prpsap2: phosphoribosyl pyrophosphate synthetase-associated protein 2
Prr3: proline rich 3
Prrx2: paired related homeobox 2
Prrxl1: dorsal root ganglia homeobox
Prss1: protease, serine, 1 (trypsin 1)
Prss12: protease, serine, 12 (neurotrypsin, motopsin)
Prss15: lon peptidase 1, mitochondrial
Prtg: protogenin homolog (Gallus gallus)
Prx: periaxin
Psat1: phosphoserine aminotransferase 1
Psd: pleckstrin and Sec7 domain containing
Psd4: pleckstrin and Sec7 domain containing 4
Psmc1: proteasome (prosome, macropain) 26S subunit, ATPase, 1
Psmc3: proteasome (prosome, macropain) 26S subunit, ATPase, 3
Psmc5: proteasome (prosome, macropain) 26S subunit, ATPase, 5
Psmd1: proteasome (prosome, macropain) 26S subunit, non-ATPase, 1
Psme1: proteasome (prosome, macropain) activator subunit 1 (PA28 alpha)
Psme2: proteasome (prosome, macropain) activator subunit 2 (PA28 beta)
Psme3: proteasome (prosome, macropain) activator subunit 3 (PA28 gamma; Ki)
Psph: phosphoserine phosphatase
Ptbp1: polypyrimidine tract binding protein 1
Ptbp2: polypyrimidine tract binding protein 2
Ptger2: prostaglandin E receptor 2 (subtype EP2), 53 kDa
Ptgis: prostaglandin I2 (prostacyclin) synthase
Ptgs1: prostaglandin-endoperoxide synthase 1 (prostaglandin G/H synthase and cyclooxygenase)
Pth: parathyroid hormone
Pthr2: parathyroid hormone 2 receptor
Ptk2: PTK2 protein tyrosine kinase 2
Ptn: pleiotrophin
Ptov1: prostate tumor overexpressed 1
Ptp4a1: protein tyrosine phosphatase type IVA, member 1
Ptpn11: protein tyrosine phosphatase, non-receptor type 11
Rnmt: RNA (guanine-7-) methyltransferase
Rraga: Ras-related GTP binding A
Rtn4: reticulon 4
Ryr3: ryanodine receptor 3
Sca1: ataxin 1
Sca10: ataxin 10
Scfd1: sec1 family domain containing 1
Scgb3a2: secretoglobin, family 3A, member 2
Scn1a: sodium channel, voltage-gated, type I, alpha subunit
Scn2a1: sodium channel, voltage-gated, type II, alpha subunit
Scp2: sterol carrier protein 2
Sdad1: SDA1 domain containing 1
Sdc2: syndecan 2
Sdcbp: syndecan binding protein (syntenin)
Sdfr1: neuroplastin
Sdha: succinate dehydrogenase complex, subunit A, flavoprotein (Fp)
Sec11l3: SEC11 homolog C (S. cerevisiae)
Sec31l1: SEC31 homolog A (S. cerevisiae)
Sectm1: secreted and transmembrane 1
Sema6c: sema domain, transmembrane domain (TM), and cytoplasmic domain, (semaphorin) 6C
Sephs1: selenophosphate synthetase 1
Serinc3: serine incorporator 3
Serpinb3: serpin peptidase inhibitor, clade B (ovalbumin), member 3
Serpine2: serpin peptidase inhibitor, clade E (nexin, plasminogen activator inhibitor type 1), member 2
Sez6: seizure related 6 homolog (mouse)
Sf3b1: splicing factor 3b, subunit 1, 155 kDa
Sfpq: splicing factor proline/glutamine-rich (polypyrimidine tract binding protein associated)
Sfrp4: secreted frizzled-related protein 4
Sfrs10: transformer 2 beta homolog (Drosophila)
Sfrs8: splicing factor, arginine/serine-rich 8 (suppressor-of-white-apricot homolog, Drosophila)
Sfrs9: splicing factor, arginine/serine-rich 9
Sftpb: surfactant protein B
Sfxn3: sideroflexin 3
Sfxn5: sideroflexin 5
Sgca: sarcoglycan, alpha (50 kDa dystrophin-associated glycoprotein)
Sgtb: small glutamine-rich tetratricopeptide repeat (TPR)-containing, beta
Sh2d4a: SH2 domain containing 4A
Sh3bp4: SH3-domain binding protein 4
Sh3bp5: SH3-domain binding protein 5 (BTK-associated)
Sh3gl3: SH3-domain GRB2-like 3
Sh3glb2: SH3-domain GRB2-like endophilin B2
Shank1: SH3 and multiple ankyrin repeat domains 1
Shank2: SH3 and multiple ankyrin repeat domains 2
Shc1: SHC (Src homology 2 domain containing) transforming protein 1
Shh: sonic hedgehog homolog (Drosophila)
Shoc2: soc-2 suppressor of clear homolog (C. elegans)
Siat7A: ST6 (alpha-N-acetyl-neuraminyl-2,3-beta-galactosyl-1,3)-N-acetylgalactosaminide alpha-2,6-sialyltransferase 1
Sipa1: signal-induced proliferation-associated 1
Sipa1l2: signal-induced proliferation-associated 1 like 2
Sipa1l3: signal-induced proliferation-associated 1 like 3
Sirpa: signal-regulatory protein alpha
Sirt2: sirtuin (silent mating type information regulation 2 homolog) 2 (S. cerevisiae)
Sirt5: sirtuin (silent mating type information regulation 2 homolog) 5 (S. cerevisiae)
Slc11a2: solute carrier family 11 (proton-coupled divalent metal ion transporters), member 2
Slc12a1: solute carrier family 12 (sodium/potassium/chloride transporters), member 1
Slc12a5: solute carrier family 12 (potassium-chloride transporter), member 5
Slco3a1: solute carrier organic anion transporter family, member 3A1
Slit1: slit homolog 1 (Drosophila)
Slit2: slit homolog 2 (Drosophila)
Slit3: slit homolog 3 (Drosophila)
Slk: STE20-like kinase (yeast)
Slu7: SLU7 splicing factor homolog (S. cerevisiae)
Smad1: SMAD family member 1
Smarca2: SWI/SNF related, matrix associated, actin dependent regulator of chromatin, subfamily a, member 2
Smc1l1: structural maintenance of chromosomes 1A
Smo: smoothened homolog (Drosophila)
Smoc1: SPARC related modular calcium binding 1
Smu1: smu-1 suppressor of mec-8 and unc-52 homolog (C. elegans)
Sn: sialic acid binding Ig-like lectin 1, sialoadhesin
Snai1: snail homolog 1 (Drosophila)
Snai2: snail homolog 2 (Drosophila)
Snap23: synaptosomal-associated protein, 23 kDa
Snap25: synaptosomal-associated protein, 25 kDa
Snap91: synaptosomal-associated protein, 91 kDa homolog (mouse)
Snca: synuclein, alpha (non A4 component of amyloid precursor)
Sncb: synuclein, beta
Snd1: staphylococcal nuclease and tudor domain containing 1
Snph: syntaphilin
Snrk: SNF related kinase
Snx17: sorting nexin 17
Snx25: sorting nexin 25
Snx3: sorting nexin 3
Soat1: sterol O-acyltransferase 1
Socs1: suppressor of cytokine signaling 1
SOD1: superoxide dismutase 1, soluble
Sod2: superoxide dismutase 2, mitochondrial
Sord: sorbitol dehydrogenase
Sox10: SRY (sex determining region Y)-box 10
Sp2: Sp2 transcription factor
Sparc: secreted protein, acidic, cysteine-rich (osteonectin)
Spata20: spermatogenesis associated 20
Spint1: serine peptidase inhibitor, Kunitz type 1
Spp1: secreted phosphoprotein 1
Spsb4: splA/ryanodine receptor domain and SOCS box containing 4
Sqle: squalene epoxidase
Src: v-src sarcoma (Schmidt-Ruppin A-2) viral oncogene homolog (avian)
Srd5a2: steroid-5-alpha-reductase, alpha polypeptide 2
Srebf1: sterol regulatory element binding transcription factor 1
Srprb: signal recognition particle receptor, B subunit
Ssb: Sjogren syndrome antigen B (autoantigen La)
Ssr4: signal sequence receptor, delta (translocon-associated protein delta)
Ssrp1: structure specific recognition protein 1
Sstr2: somatostatin receptor 2
Ssx2ip: synovial sarcoma, X breakpoint 2 interacting protein
St14: suppression of tumorigenicity 14 (colon carcinoma)
St18: suppression of tumorigenicity 18 (breast carcinoma) (zinc finger protein)
St3gal1: ST3 beta-galactoside alpha-2,3-sialyltransferase 1
St6gal2: ST6 beta-galactosamide alpha-2,6-sialyltransferase 2
St7: suppression of tumorigenicity 7
St7l: suppression of tumorigenicity 7 like
Star: steroidogenic acute regulatory protein
Stard3nl: STARD3 N-terminal like
Stat1: signal transducer and activator of transcription 1, 91 kDa
Stat3: signal transducer and activator of transcription 3 (acute-phase response factor)
Stau1: staufen, RNA binding protein, homolog 1 (Drosophila)
Stch: heat shock protein 70 kDa family, member 13
Ste: sulfotransferase family 1E, estrogen-preferring, member 1
Stim2: stromal interaction molecule 2
Stip1: stress-induced-phosphoprotein 1
Stk10: serine/threonine kinase 10
Stk11: serine/threonine kinase 11
Stmn1: stathmin 1
Stmn2: stathmin-like 2
Stmn3: stathmin-like 3
Stmn4: stathmin-like 4
Stnl: eukaryotic translation elongation factor 1 alpha 2
Stom: stomatin
Strbp: spermatid perinuclear RNA binding protein
Strn: striatin, calmodulin binding protein
Stx12: syntaxin 12
Stx1a: syntaxin 1A (brain)
Stx4a: syntaxin 4
Stx5a: syntaxin 5
Stx6: syntaxin 6
Stx8: syntaxin 8
Stxbp3: syntaxin binding protein 3
Stxbp5: syntaxin binding protein 5 (tomosyn)
Suclg1: succinate-CoA ligase, alpha subunit
Sult1a1: sulfotransferase family, cytosolic, 1A, phenol-preferring, member 1
Sult1c1: sulfotransferase family, cytosolic, 1C, member 2
Sult1c2: sulfotransferase family, cytosolic, 1C, member 2
Sult1e1: sulfotransferase family 1E, estrogen-preferring, member 1
Sult4a1: sulfotransferase family 4A, member 1
Supv3l1: suppressor of var1, 3-like 1 (S. cerevisiae)
Surf6: surfeit 6
Suv420h2: suppressor of variegation 4-20 homolog 2 (Drosophila)
Sv2a: synaptic vesicle glycoprotein 2A
Sv2b: synaptic vesicle glycoprotein 2B
Sycp1: synaptonemal complex protein 1
Sycp2: synaptonemal complex protein 2
Syn1: synapsin I
Syn2: synapsin II
Syn3: synapsin III
Syngap1: synaptic Ras GTPase activating protein 1 homolog (rat)
Synj1: synaptojanin 1
Synj2: synaptojanin 2
Synj2bp: synaptojanin 2 binding protein
Synpo: synaptopodin
Sypl: synaptophysin-like 1
Syt11: synaptotagmin XI
Syt12: synaptotagmin XII
Syt5: synaptotagmin V
Syt6: synaptotagmin VI
Syt8: synaptotagmin VIII
Tacc1: transforming, acidic coiled-coil containing protein 1
Tacc2: transforming, acidic coiled-coil containing protein 2
Tacr1: tachykinin receptor 1
Taf1c: TATA box binding protein (TBP)-associated factor, RNA polymerase I, C, 110 kDa
Taf9l: TAF9B RNA polymerase II, TATA box binding protein (TBP)-associated factor, 31 kDa
Tagln: transgelin
Tagln3: transgelin 3
Taldo1: transaldolase 1
Taok1: TAO kinase 1
Tapbp: TAP binding protein (tapasin)
Tars: threonyl-tRNA synthetase
Tas2r41: taste receptor, type 2, member 41
Tat: tyrosine aminotransferase
Tax1bp1: Tax1 (human T-cell leukemia virus type I) binding protein 1
Tbc1d10b: TBC1 domain family, member 10B
Tbca: tubulin folding cofactor A
Tbx2: T-box 2
Tcirg1: T-cell, immune regulator 1, ATPase, H+ transporting, lysosomal V0 subunit A3
Tcn2: transcobalamin II; macrocytic anemia
Tcp1: t-complex 1
Tcra: T cell receptor alpha locus
Tdg: thymine-DNA glycosylase
Tdrd7: tudor domain containing 7
Tead1: TEA domain family member 1 (SV40 transcriptional enhancer factor)
Tegt: transmembrane BAX inhibitor motif containing 6
Tg: thyroglobulin
Tgfb1: transforming growth factor, beta 1
Tgfbr3: transforming growth factor, beta receptor III
Tgm1: transglutaminase 1 (K polypeptide epidermal type I, protein-glutamine-gamma-glutamyltransferase)
Tgm2: transglutaminase 2 (C polypeptide, protein-glutamine-gamma-glutamyltransferase)
Thbd: thrombomodulin
Thbs4: thrombospondin 4
Thpo: thrombopoietin
Thra: thyroid hormone receptor, alpha (erythroblastic leukemia viral (v-erb-a) oncogene homolog, avian)
Thrap3: thyroid hormone receptor associated protein 3
Thrb: thyroid hormone receptor, beta (erythroblastic leukemia viral (v-erb-a) oncogene homolog 2, avian)
Thy1: Thy-1 cell surface antigen
Tigd3: tigger transposable element derived 3
Timm44: translocase of inner mitochondrial membrane 44 homolog (yeast)
Timm8a: translocase of inner mitochondrial membrane 8 homolog A (yeast)
Tinag: tubulointerstitial nephritis antigen
Tjp1: tight junction protein 1 (zona occludens 1)
Tjp2: tight junction protein 2 (zona occludens 2)
Tkt: transketolase
Tle3: transducin-like enhancer of split 3 (E(sp1) homolog, Drosophila)
Tlr3: toll-like receptor 3
Tlr4: toll-like receptor 4
Tlr5: toll-like receptor 5
Tlr6: toll-like receptor 6
Tlr9: toll-like receptor 9
Tm4sf8: tetraspanin 3
Tm9sf2: transmembrane 9 superfamily member 2
Tm9sf3: transmembrane 9 superfamily member 3
Tmco1: transmembrane and coiled-coil domains 1
Tmeff1: transmembrane protein with EGF-like and two follistatin-like domains 1
Tmem27: transmembrane protein 27
Tmf1: TATA element modulatory factor 1
Tmpo: thymopoietin
Tmsb10: thymosin beta 10
Tnfaip1: tumor necrosis factor, alpha-induced protein 1 (endothelial)
Tnni1: troponin I type 1 (skeletal, slow)
Tnni2: troponin I type 2 (skeletal, fast)
Tnnt3: troponin T type 3 (skeletal, fast)
Tob1: transducer of ERBB2, 1
Tob2: transducer of ERBB2, 2
Tom1: target of myb1 (chicken)
Tor3a: torsin family 3, member A
Tpbg: trophoblast glycoprotein
Tpd52l2: tumor protein D52-like 2
Tph2: tryptophan hydroxylase 2
Tpm3: tropomyosin 3
Tpo: thyroid peroxidase
Tpst2: tyrosylprotein sulfotransferase 2
Tpt1: tumor protein, translationally-controlled 1
Tr4: nuclear receptor subfamily 2, group C, member 2
Traf3ip1: TNF receptor-associated factor 3 interacting protein 1
Trak2: trafficking protein, kinesin binding 2
Trap1: TNF receptor-associated protein 1
Trib3: tribbles homolog 3 (Drosophila)
Trim13: tripartite motif-containing 13
Trim54: tripartite motif-containing 54
Trip10: thyroid hormone receptor interactor 10
Trpc2: transient receptor potential cation channel, subfamily C, member 2 (pseudogene)
Trpc4: transient receptor potential cation channel, subfamily C, member 4
Trpc5: transient receptor potential cation channel, subfamily C, member 5
Trpc7: transient receptor potential cation channel, subfamily C, member 7
Trpv1: transient receptor potential cation channel, subfamily V, member 1
Trpv4: transient receptor potential cation channel, subfamily V, member 4
Tsc1: tuberous sclerosis 1
Tsc2: tuberous sclerosis 2
Tsg101: tumor susceptibility gene 101
Tsga10: testis specific, 10
Tsga10ip: testis specific, 10 interacting protein
Tshr: thyroid stimulating hormone receptor
Tsn: translin
Tsnax: translin-associated factor X
Tst: thiosulfate sulfurtransferase (rhodanese)
Ttc1: tetratricopeptide repeat domain 1
Ttn: titin
Tub: tubby homolog (mouse)
Tuba1a: tubulin, alpha 1a
Tubb2b: tubulin, beta 2B
Tubb2c: tubulin, beta 2C
Tubb3: tubulin, beta 3
Tusc3: tumor suppressor candidate 3
Txn: thioredoxin
Txn2: thioredoxin 2
Txndc4: endoplasmic reticulum protein 44
Txnip: thioredoxin interacting protein
Txnl2: glutaredoxin 3
Txnrd1: thioredoxin reductase 1
Txnrd2: thioredoxin reductase 2
Ubc: ubiquitin C
Ube2e2: ubiquitin-conjugating enzyme E2E 2 (UBC4/5 homolog, yeast)
Ubl3: ubiquitin-like 3
Ubqln1: ubiquilin 1
Ubxd3: UBX domain protein 10
Uchl1: ubiquitin carboxyl-terminal esterase L1 (ubiquitin thiolesterase)
Ucp1: uncoupling protein 1 (mitochondrial, proton carrier)
Ucp2: uncoupling protein 2 (mitochondrial, proton carrier)
Ugt1a1: UDP glucuronosyltransferase 1 family, polypeptide A1
Umod: uromodulin
Unc13a: unc-13 homolog A (C. elegans)
Unc13b: unc-13 homolog B (C. elegans)
Unc13c: unc-13 homolog C (C. elegans)
Unc5b: unc-5 homolog B (C. elegans)
Uqcrc2: ubiquinol-cytochrome c reductase core protein II
Uqcrfs1: ubiquinol-cytochrome c reductase, Rieske iron-sulfur polypeptide 1
Uso1: USO1 homolog, vesicle docking protein (yeast)
Usp11: ubiquitin specific peptidase 11
Vamp1: vesicle-associated membrane protein 1 (synaptobrevin 1)
Vamp2: vesicle-associated membrane protein 2 (synaptobrevin 2)
Vamp3: vesicle-associated membrane protein 3 (cellubrevin)
Vapa: VAMP (vesicle-associated membrane protein)-associated protein A, 33 kDa
Vapb: VAMP (vesicle-associated membrane protein)-associated protein B and C
VCAN: versican
Vcan: versican
Vcp: valosin-containing protein
Vcpip1: valosin containing protein (p97)/p47 complex interacting protein 1
Vdac1: voltage-dependent anion channel 1
Vdac2: voltage-dependent anion channel 2
Vegfa: vascular endothelial growth factor A
Vgcnl1: sodium leak channel, non-selective
Vgf: VGF nerve growth factor inducible
Vipr2: vasoactive intestinal peptide receptor 2
Vpreb1: pre-B lymphocyte 1
Vps33a: vacuolar protein sorting 33 homolog A (S. cerevisiae)
Vps33b: vacuolar protein sorting 33 homolog B (yeast)
Vps4a: vacuolar protein sorting 4 homolog A (S. cerevisiae)
Vsnl1: visinin-like 1
Vtn: vitronectin
Wars: tryptophanyl-tRNA synthetase
Wbp2: WW domain binding protein 2
Wdr10: intraflagellar transport 122 homolog (Chlamydomonas)
Wdr44: WD repeat domain 44
Wdr7: WD repeat domain 7
Wfs1: Wolfram syndrome 1 (wolframin)
Wif1: WNT inhibitory factor 1
Wipf1: WAS/WASL interacting protein family, member 1
Wnk4: WNK lysine deficient protein kinase 4
Wnt5a: wingless-type MMTV integration site family, member 5A
Wnt7a: wingless-type MMTV integration site family, member 7A
Xkr6: XK, Kell blood group complex subunit-related family, member 6
Xpnpep2: X-prolyl aminopeptidase (aminopeptidase P) 2, membrane-bound
Xpo1: exportin 1 (CRM1 homolog, yeast)
Xpo6: exportin 6
Xrcc1: X-ray repair complementing defective repair in Chinese hamster cells 1
Xrcc5: X-ray repair complementing defective repair in Chinese hamster cells 5
Xylt1: xylosyltransferase I
Yars2: tyrosyl-tRNA synthetase 2, mitochondrial
Yes1: v-yes-1 Yamaguchi sarcoma viral oncogene homolog 1
Ykt6: YKT6 v-SNARE homolog (S. cerevisiae)
Ywhab: tyrosine 3-monooxygenase/tryptophan 5-monooxygenase activation protein, beta polypeptide
Yy1: YY1 transcription factor
Znf382: zinc finger protein 382
Zranb1: zinc finger, RAN-binding domain containing 1
Zwint: ZW10 interactor.

### B.

Lipid raft-derived extracted proteins from Alzheimer's disease (3xTgAD) mouse 16-month-old cortical tissue. Specific proteins isolated from 3xTgAD mouse cortex were identified with multidimensional protein identification technology (MudPIT) LC MS/MS. Each protein was identified in at least two out of three experimental replicate animals and from at least two isolated peptides per protein. The following are the protein symbol and its corresponding definition.

Abca1: ATP-binding cassette, sub-family A (ABC1), member 1
ABCA7: ATP-binding cassette, sub-family A (ABC1), member 7
Abcb4: ATP-binding cassette, sub-family B (MDR/TAP), member 4
Abcc1: ATP-binding cassette, sub-family C (CFTR/MRP), member 1
Abcc2: ATP-binding cassette, sub-family C (CFTR/MRP), member 2
Abcc4: ATP-binding cassette, sub-family C (CFTR/MRP), member 4
Abi1: abl-interactor 1
Acadl: acyl-Coenzyme A dehydrogenase, long chain
Acat1: acetyl-Coenzyme A acetyltransferase 1
Acin1: apoptotic chromatin condensation inducer 1
Acmsd: aminocarboxymuconate semialdehyde decarboxylase
Acp2: acid phosphatase 2, lysosomal
Acta1: actin, alpha 1, skeletal muscle
Actb: actin, beta
Actn4: actinin, alpha 4
Acvr1: activin A receptor, type I
Adam2: ADAM metallopeptidase domain 2
Adam6: ADAM metallopeptidase domain 6 (pseudogene)
Adcy1: adenylate cyclase 1 (brain)
Adcy5: adenylate cyclase 5
Adcy8: adenylate cyclase 8 (brain)
Adcyap1: adenylate cyclase activating polypeptide 1 (pituitary)
Adcyap1r1: adenylate cyclase activating polypeptide 1 (pituitary) receptor type I
Add1: adducin 1 (alpha)
Add2: adducin 2 (beta)
Adrbk1: adrenergic, beta, receptor kinase 1
Aes: amino-terminal enhancer of split
Agpat1: 1-acylglycerol-3-phosphate O-acyltransferase 1 (lysophosphatidic acid acyltransferase, alpha)
Agpat4: 1-acylglycerol-3-phosphate O-acyltransferase 4 (lysophosphatidic acid acyltransferase, delta)
Agrn: agrin
Ahi1: Abelson helper integration site 1
Ahr: aryl hydrocarbon receptor
Ahrr: aryl-hydrocarbon receptor repressor
Ak3: adenylate kinase 3
Akap12: A kinase (PRKA) anchor protein 12
Akap3: A kinase (PRKA) anchor protein 3
Akap4: A kinase (PRKA) anchor protein 4
Akap9: A kinase (PRKA) anchor protein (yotiao) 9
Alad: aminolevulinate, delta-, dehydratase
Aldh1a3: aldehyde dehydrogenase 1 family, member A3
Alox5: arachidonate 5-lipoxygenase
Ampd1: adenosine monophosphate deaminase 1 (isoform M)
Anxa3: annexin A3
Anxa6: annexin A6
Apex2: APEX nuclease (apurinic/apyrimidinic endonuclease) 2
Apob: apolipoprotein B (including Ag(x) antigen)
Apod: apolipoprotein D
App: amyloid beta (A4) precursor protein
Aps: kallikrein-related peptidase 3
Aqp3: aquaporin 3 (Gill blood group)
Aqp4: aquaporin 4
Aqp8: aquaporin 8
Arf1: ADP-ribosylation factor 1
Arf4: ADP-ribosylation factor 4
Arf6: ADP-ribosylation factor 6
Arfip1: ADP-ribosylation factor interacting protein 1
Arhgap20: Rho GTPase activating protein 20
Arhgap8: Rho GTPase activating protein 8
Arhgdia: Rho GDP dissociation inhibitor (GDI) alpha
Arhgef11: Rho guanine nucleotide exchange factor (GEF) 11
Arhgef12: Rho guanine nucleotide exchange factor (GEF) 12
Arhgef6: Rac/Cdc42 guanine nucleotide exchange factor (GEF) 6
Arnt: aryl hydrocarbon receptor nuclear translocator
Arntl: aryl hydrocarbon receptor nuclear translocator-like
Arpp19: cAMP-regulated phosphoprotein, 19 kDa
Ascc1: activating signal cointegrator 1 complex subunit 1
Asz1: ankyrin repeat, SAM and basic leucine zipper domain containing 1
Atcay: ataxia, cerebellar, Cayman type
Atf3: activating transcription factor 3
Atm: ataxia telangiectasia mutated
Atp1a1: ATPase, Na+/K+ transporting, alpha 1 polypeptide
Atp1a4: ATPase, Na+/K+ transporting, alpha 4 polypeptide
Atp1b1: ATPase, Na+/K+ transporting, beta 1 polypeptide
ATP2B1: ATPase, Ca++ transporting, plasma membrane 1
Atp2b1: ATPase, Ca++ transporting, plasma membrane 1
Atp2b2: ATPase, Ca++ transporting, plasma membrane 2
Atp5a1: ATP synthase, H+ transporting, mitochondrial F1 complex, alpha subunit 1, cardiac muscle
Atp5b: ATP synthase, H+ transporting, mitochondrial F1 complex, beta polypeptide
Atp5f1: ATP synthase, H+ transporting, mitochondrial F0 complex, subunit B1
Atp5h: ATP synthase, H+ transporting, mitochondrial F0 complex, subunit d
Atp5i: ATP synthase, H+ transporting, mitochondrial F0 complex, subunit E
Atp5j: ATP synthase, H+ transporting, mitochondrial F0 complex, subunit F6
Atp6b2: ATPase, H+ transporting, lysosomal 56/58 kDa, V1 subunit B2
Atp6l: ATPase, H+ transporting, lysosomal 16 kDa, V0 subunit c
Atp6v0a1: ATPase, H+ transporting, lysosomal V0 subunit a1
ATPBD1C: GPN-loop GTPase 3
Atxn3: ataxin 3
Avil: advillin
B3gat1: beta-1,3-glucuronyltransferase 1 (glucuronosyltransferase P)
Bace1: beta-site APP-cleaving enzyme 1
Basp1: brain abundant, membrane attached signal protein 1
Bat2: HLA-B associated transcript 2
Bat5: HLA-B associated transcript 5
Bax: BCL2-associated X protein
Bcl10: B-cell CLL/lymphoma 10
Begain: brain-enriched guanylate kinase-associated homolog (rat)
Bmp6: bone morphogenetic protein 6
Bnip1: BCL2/adenovirus E1B 19 kDa interacting protein 1
Bnip3: BCL2/adenovirus E1B 19 kDa interacting protein 3
Bpnt1: 3^'^(2^'^), 5^'^-bisphosphate nucleotidase 1
Brd2: bromodomain containing 2
Brd8: bromodomain containing 8
Bst1: bone marrow stromal cell antigen 1
Btg2: BTG family, member 2
Bzrap1: benzodiazepine receptor (peripheral) associated protein 1
c3orf6: coiled-coil domain containing 50
C4a: complement component 4A (Rodgers blood group)
c8b: complement component 8, beta polypeptide
Cabc1: chaperone, ABC1 activity of bc1 complex homolog (S. pombe)
Cabin1: calcineurin binding protein 1
Cacna1g: calcium channel, voltage-dependent, T type, alpha 1G subunit
Cacna2d1: calcium channel, voltage-dependent, alpha 2/delta subunit 1
Cacna2d2: calcium channel, voltage-dependent, alpha 2/delta subunit 2
Cacna2d3: calcium channel, voltage-dependent, alpha 2/delta subunit 3
Cacnb2: calcium channel, voltage-dependent, beta 2 subunit
Calb1: calbindin 1, 28 kDa
Calb2: calbindin 2
Cald1: caldesmon 1
calm2: calmodulin 2 (phosphorylase kinase, delta)
Calr: calreticulin
Camk2g: calcium/calmodulin-dependent protein kinase II gamma
Camkk1: calcium/calmodulin-dependent protein kinase kinase 1, alpha
Camkv: CaM kinase-like vesicle-associated
Canx: calnexin
Cap1: CAP, adenylate cyclase-associated protein 1 (yeast)
Capn1: calpain 1, (mu/l) large subunit
Capn5: calpain 5
Capn6: calpain 6
Capzb: capping protein (actin filament) muscle Z-line, beta
Card9: caspase recruitment domain family, member 9
Carhsp1: calcium regulated heat stable protein 1, 24 kDa
Caskin1: CASK interacting protein 1
Casp7: caspase 7, apoptosis-related cysteine peptidase
Cast: calpastatin
Cbp: opsin 1 (cone pigments), long-wave-sensitive
Cbx3: chromobox homolog 3 (HP1 gamma homolog, Drosophila)
Ccnd2: cyclin D2
Ccs: copper chaperone for superoxide dismutase
Cct2: chaperonin containing TCP1, subunit 2 (beta)
Cd2ap: CD2-associated protein
Cd36: CD36 molecule (thrombospondin receptor)
Cd86: CD86 molecule
Cdc25b: cell division cycle 25 homolog B (S. pombe)
Cdc42: cell division cycle 42 (GTP binding protein, 25 kDa)
Cdc5l: CDC5 cell division cycle 5-like (S. pombe)
Cdh1: cadherin 1, type 1, E-cadherin (epithelial)
Cdh10: cadherin 10, type 2 (T2-cadherin)
Cdh13: cadherin 13, H-cadherin (heart)
Cdh2: cadherin 2, type 1, N-cadherin (neuronal)
Cdk5rap2: CDK5 regulatory subunit associated protein 2
Cdkn1b: cyclin-dependent kinase inhibitor 1B (p27, Kip1)
Cds1: CDP-diacylglycerol synthase (phosphatidate cytidylyltransferase) 1
Cdv1: intraflagellar transport 81 homolog (Chlamydomonas)
Cebp: CCAAT/enhancer binding protein (C/EBP), alpha
Cend1: cell cycle exit and neuronal differentiation 1
Cenpc1: centromere protein C 1
Cenpi: centromere protein I
Centa1: ArfGAP with dual PH domains 1
Ces1: carboxylesterase 1 (monocyte/macrophage serine esterase 1)
Cetn2: centrin, EF-hand protein, 2
Cfd: complement factor D (adipsin)
Cfl1: cofilin 1 (nonmuscle)
Cftr: cystic fibrosis transmembrane conductance regulator (ATP-binding cassette sub-family C, member 7)
Chgb: chromogranin B (secretogranin 1)
Chka: choline kinase alpha
Chm: choroideremia (Rab escort protein 1)
Chst10: carbohydrate sulfotransferase 10
Chx10: visual system homeobox 2
Cktsf1b1: gremlin 1, cysteine knot superfamily, homolog (Xenopus laevis)
Clcf1: cardiotrophin-like cytokine factor 1
Cldn18: claudin 18
Clic4: chloride intracellular channel 4
Clip3: CAP-GLY domain containing linker protein 3
Clta: clathrin, light chain (Lca)
Cltb: clathrin, light chain (Lcb)
Cltc: clathrin, heavy chain (Hc)
Clu: clusterin
Cnga1: cyclic nucleotide gated channel alpha 1
Cngb1: cyclic nucleotide gated channel beta 1
Cnot4: CCR4-NOT transcription complex, subunit 4
Cnr1: cannabinoid receptor 1 (brain)
Col5a3: collagen, type V, alpha 3
Copb1: coatomer protein complex, subunit beta 1
Cops4: COP9 constitutive photomorphogenic homolog subunit 4 (Arabidopsis)
Coro1a: coronin, actin binding protein, 1A
Cp: ceruloplasmin (ferroxidase)
Cpa2: carboxypeptidase A2 (pancreatic)
Cpt2: carnitine palmitoyltransferase 2
Creb1: cAMP responsive element binding protein 1
Crhbp: corticotropin releasing hormone binding protein
Crip2: cysteine-rich protein 2
Cript: cysteine-rich PDZ-binding protein
Crmp1: collapsin response mediator protein 1
Cry2: cryptochrome 2 (photolyase-like)
Csf1: colony stimulating factor 1 (macrophage)
Csh1: chorionic somatomammotropin hormone 1 (placental lactogen)
Csnk1e: casein kinase 1, epsilon
Csnk1g1: casein kinase 1, gamma 1
Csnk1g3: casein kinase 1, gamma 3
Cspg6: structural maintenance of chromosomes 3
Cst6: cystatin E/M
Cstb: cystatin B (stefin B)
Ctnnb1: catenin (cadherin-associated protein), beta 1, 88 kDa
Cttn: cortactin
Cugbp2: CUG triplet repeat, RNA binding protein 2
Cul5: cullin 5
Cutl1: cut-like homeobox 1
Cxadr: coxsackie virus and adenovirus receptor
Cxcl10: chemokine (C-X-C motif) ligand 10
Cyb5r4: cytochrome b5 reductase 4
Cyln2: CAP-GLY domain containing linker protein 2
Cyp19a1: cytochrome P450, family 19, subfamily A, polypeptide 1
Cyp1a1: cytochrome P450, family 1, subfamily A, polypeptide 1
Cyp4x1: cytochrome P450, family 4, subfamily X, polypeptide 1
Dab2ip: DAB2 interacting protein
Dbn1: drebrin 1
Dctn1: dynactin 1 (p150, glued homolog, Drosophila)
Dctn2: dynactin 2 (p50)
Dctn4: dynactin 4 (p62)
Dcxr: dicarbonyl/L-xylulose reductase
Ddah2: dimethylarginine dimethylaminohydrolase 2
Ddb1: damage-specific DNA binding protein 1, 127 kDa
Ddt: D-dopachrome tautomerase
Ddx1: DEAD (Asp-Glu-Ala-Asp) box polypeptide 1
Ddx19: DEAD (Asp-Glu-Ala-As) box polypeptide 19B
Ddx27: DEAD (Asp-Glu-Ala-Asp) box polypeptide 27
Ddx5: DEAD (Asp-Glu-Ala-Asp) box polypeptide 5
Dedd: death effector domain containing
Degs1: degenerative spermatocyte homolog 1, lipid desaturase (Drosophila)
Des: desmin
Dgkg: diacylglycerol kinase, gamma 90 kDa
Dhcr7: 7-dehydrocholesterol reductase
Dhodh: dihydroorotate dehydrogenase
Dhx40: DEAH (Asp-Glu-Ala-His) box polypeptide 40
Dia1: cytochrome b5 reductase 3
Disc1: disrupted in schizophrenia 1
Dkc1: dyskeratosis congenita 1, dyskerin
Dlat: dihydrolipoamide S-acetyltransferase
Dlc1: deleted in liver cancer 1
Dlgap2: discs, large (Drosophila) homolog-associated protein 2
Dlgap4: discs, large (Drosophila) homolog-associated protein 4
Dll1: delta-like 1 (Drosophila)
Dnah7: dynein, axonemal, heavy chain 7
Dnah9: dynein, axonemal, heavy chain 9
Dnajc5: DnaJ (Hsp40) homolog, subfamily C, member 5
Dnch1: dynein, cytoplasmic 1, heavy chain 1
Dnch2: dynein, cytoplasmic 2, heavy chain 1
Dnm1: dynamin 1
Dnmt1: DNA (cytosine-5-)-methyltransferase 1
Dnmt3a: DNA (cytosine-5-)-methyltransferase 3 alpha
Dntt: deoxynucleotidyltransferase, terminal
Dpp3: dipeptidyl-peptidase 3
Dpp6: dipeptidyl-peptidase 6
Dpyd: dihydropyrimidine dehydrogenase
Drd1ip: calcyon neuron-specific vesicular protein
Drg1: developmentally regulated GTP binding protein 1
Dtnb: dystrobrevin, beta
Dtnbp1: dystrobrevin binding protein 1
Duox1: dual oxidase 1
Dvl1: dishevelled, dsh homolog 1 (Drosophila)
Dyx1c1: dyslexia susceptibility 1 candidate 1
Eaf2: ELL associated factor 2
Echs1: enoyl Coenzyme A hydratase, short chain, 1, mitochondrial
Eef1g: eukaryotic translation elongation factor 1 gamma
Eef2: eukaryotic translation elongation factor 2
Eef2k: eukaryotic elongation factor-2 kinase
Efemp2: EGF-containing fibulin-like extracellular matrix protein 2
Egfr: epidermal growth factor receptor (erythroblastic leukemia viral (v-erb-b) oncogene homolog, avian)
Eif2ak3: eukaryotic translation initiation factor 2-alpha kinase 3
Eif2b1: eukaryotic translation initiation factor 2B, subunit 1 alpha, 26 kDa
Eif2c2: eukaryotic translation initiation factor 2C, 2
Eif2s2: eukaryotic translation initiation factor 2, subunit 2 beta, 38 kDa
eif4a1: eukaryotic translation initiation factor 4A, isoform 1
Elavl3: ELAV (embryonic lethal, abnormal vision, Drosophila)-like 3 (Hu antigen C)
ELK1: ELK1, member of ETS oncogene family
Emd: emerin
Eno2: enolase 2 (gamma, neuronal)
Enpp2: ectonucleotide pyrophosphatase/phosphodiesterase 2
Entpd8: ectonucleoside triphosphate diphosphohydrolase 8
Ephx2: epoxide hydrolase 2, cytoplasmic
Epn1: epsin 1
Erp29: endoplasmic reticulum protein 29
Esd: esterase D/formylglutathione hydrolase
Espn: espin
Esrrb: estrogen-related receptor beta
Etfa: electron-transfer-flavoprotein, alpha polypeptide
Etfb: electron-transfer-flavoprotein, beta polypeptide
Exoc7: exocyst complex component 7
Ezr: ezrin
F5: coagulation factor V (proaccelerin, labile factor)
Fabp1: fatty acid binding protein 1, liver
Fancd2: Fanconi anemia, complementation group D2
Fat: FAT tumor suppressor homolog 1 (Drosophila)
Fat2: FAT tumor suppressor homolog 2 (Drosophila)
Fat3: FAT tumor suppressor homolog 3 (Drosophila)
Fbn1: fibrillin 1
Fdps: farnesyl diphosphate synthase
Fgfr1op2: FGFR1 oncogene partner 2
Filip1: filamin A interacting protein 1
Fkbp1b: FK506 binding protein 1B, 12.6 kDa
Flg: filaggrin
Flot1: flotillin 1
Flot2: flotillin 2
Fosl1: FOS-like antigen 1
Fosl2: FOS-like antigen 2
Fpgt: fucose-1-phosphate guanylyltransferase
Frap1: mechanistic target of rapamycin (serine/threonine kinase)
Freq: frequenin homolog (Drosophila)
Fyn: FYN oncogene related to SRC, FGR, YES
Gaa: glucosidase, alpha; acid
Gabbr1: gamma-aminobutyric acid (GABA) B receptor, 1
Gabra3: gamma-aminobutyric acid (GABA) A receptor, alpha 3
Gabre: gamma-aminobutyric acid (GABA) A receptor, epsilon
Gadd45a: growth arrest and DNA-damage-inducible, alpha
Galc: galactosylceramidase
Galk1: galactokinase 1
Galnt10: UDP-N-acetyl-alpha-D-galactosamine: polypeptide N-acetylgalactosaminyltransferase 10
Gars: glycyl-tRNA synthetase
Gas7: growth arrest-specific 7
Gc: group-specific component (vitamin D binding protein)
Gdi1: GDP dissociation inhibitor 1
Gdi2: GDP dissociation inhibitor 2
Gfap: glial fibrillary acidic protein
Gif: gastric intrinsic factor (vitamin B synthesis)
Gjb2: gap junction protein, beta 2, 26 kDa
Gla: galactosidase, alpha
Gldc: glycine dehydrogenase (decarboxylating)
Glra2: glycine receptor, alpha 2
Glut3: solute carrier family 2 (facilitated glucose transporter), member 3
Gmcl1: germ cell-less homolog 1 (Drosophila)
Gmfb: glia maturation factor, beta
Gna11: guanine nucleotide binding protein (G protein), alpha 11 (Gq class)
Gnai1: guanine nucleotide binding protein (G protein), alpha inhibiting activity polypeptide 1
Gnai2: guanine nucleotide binding protein (G protein), alpha inhibiting activity polypeptide 2
Gnao1: guanine nucleotide binding protein (G protein), alpha activating activity polypeptide O
Gnaq: guanine nucleotide binding protein (G protein), q polypeptide
Gnaz: guanine nucleotide binding protein (G protein), alpha z polypeptide
Gnb2l1: guanine nucleotide binding protein (G protein), beta polypeptide 2-like 1
Gnl3: guanine nucleotide binding protein-like 3 (nucleolar)
Gnpat: glyceronephosphate O-acyltransferase
Gpi: glucose phosphate isomerase
Gpm6a: glycoprotein M6A
Gpr1: G protein-coupled receptor 1
Gpr141: G protein-coupled receptor 141
Gpr56: G protein-coupled receptor 56
Gpsm1: G-protein signaling modulator 1 (AGS3-like, C. elegans)
Gpt2: glutamic pyruvate transaminase (alanine aminotransferase) 2
Grin1: glutamate receptor, ionotropic, N-methyl D-aspartate 1
Grpel1: GrpE-like 1, mitochondrial (E. coli)
Gstm2: glutathione S-transferase mu 2 (muscle)
Gstm3: glutathione S-transferase mu 3 (brain)
Gucy2d: guanylate cyclase 2D, membrane (retina-specific)
Gucy2f: guanylate cyclase 2F, retinal
H1f0: H1 histone family, member 0
H1f4: histone cluster 1, H1e
H2afy: H2A histone family, member Y
Haao: 3-hydroxyanthranilate 3,4-dioxygenase
Hap1: huntingtin-associated protein 1
Hapln3: hyaluronan and proteoglycan link protein 3
Hbld2: iron-sulfur cluster assembly 1 homolog (S. cerevisiae)
Hck: hemopoietic cell kinase
Hcn1: hyperpolarization activated cyclic nucleotide-gated potassium channel 1
Hdac6: histone deacetylase 6
Hdgf: hepatoma-derived growth factor (high-mobility group protein 1-like)
Hdlbp: high density lipoprotein binding protein
Hey1: hairy/enhancer-of-split related with YRPW motif 1
Hgf: hepatocyte growth factor (hepapoietin A; scatter factor)
Hip1r: huntingtin interacting protein 1 related
Hivep1: human immunodeficiency virus type I enhancer binding protein 1
Hk1: hexokinase 1
Hmga2: high mobility group AT-hook 2
Hmgcr: 3-hydroxy-3-methylglutaryl-Coenzyme A reductase
Hmgcs1: 3-hydroxy-3-methylglutaryl-Coenzyme A synthase 1 (soluble)
Hmgn3: high mobility group nucleosomal binding domain 3
Hn1: hematological and neurological expressed 1
Hnf4a: hepatocyte nuclear factor 4, alpha
Hnmt: histamine N-methyltransferase
Hnrnph1: heterogeneous nuclear ribonucleoprotein H1 (H)
Hnrpa1: heterogeneous nuclear ribonucleoprotein A1
Hnrpk: heterogeneous nuclear ribonucleoprotein K
Hnrpu: heterogeneous nuclear ribonucleoprotein U (scaffold attachment factor A)
hook3: hook homolog 3 (Drosophila)
Hps5: Hermansky-Pudlak syndrome 5
Hpse: heparanase
Hras1: v-Ha-ras Harvey rat sarcoma viral oncogene homolog
Hsd11b2: hydroxysteroid (11-beta) dehydrogenase 2
Hsd17B1: hydroxysteroid (17-beta) dehydrogenase 1
Hsf1: heat shock transcription factor 1
Hsp90ab1: heat shock protein 90 kDa alpha (cytosolic), class B member 1
Hspa14: heat shock 70 kDa protein 14
Hspe1: heat shock 10 kDa protein 1 (chaperonin 10)
Htr2c: 5-hydroxytryptamine (serotonin) receptor 2C
Htr3a: 5-hydroxytryptamine (serotonin) receptor 3A
Htr4: 5-hydroxytryptamine (serotonin) receptor 4
Hyou1: hypoxia up-regulated 1
Id2: inhibitor of DNA binding 2, dominant negative helix-loop-helix protein
Idh1: isocitrate dehydrogenase 1 (NADP+), soluble
Igf1r: insulin-like growth factor 1 receptor
Igf2r: insulin-like growth factor 2 receptor
Ikbkap: inhibitor of kappa light polypeptide gene enhancer in B-cells, kinase complex-associated protein
IL1b: interleukin 1, beta
Il1r2: interleukin 1 receptor, type II
Il1rapl1: interleukin 1 receptor accessory protein-like 1
Il6: interleukin 6 (interferon, beta 2)
Ilf3: interleukin enhancer binding factor 3, 90 kDa
Inhbc: inhibin, beta C
Inpp4a: inositol polyphosphate-4-phosphatase, type I, 107 kDa
Inpp4b: inositol polyphosphate-4-phosphatase, type II, 105 kDa
Inpp5d: inositol polyphosphate-5-phosphatase, 145 kDa
Irs1: insulin receptor substrate 1
Irs2: insulin receptor substrate 2
Isg20: interferon stimulated exonuclease gene 20 kDa
Itga6: integrin, alpha 6
Itm2c: integral membrane protein 2C
Itpr2: inositol 1,4,5-triphosphate receptor, type 2
Itpr3: inositol 1,4,5-triphosphate receptor, type 3
Ivl: involucrin
Jag1: jagged 1 (Alagille syndrome)
Jak1: Janus kinase 1
Jak2: Janus kinase 2
Junb: jun B proto-oncogene
Kalrn: kalirin, RhoGEF kinase
Katna1: katanin p60 (ATPase-containing) subunit A 1
Kcnh1: potassium voltage-gated channel, subfamily H (eag-related), member 1
Kcnh2: potassium voltage-gated channel, subfamily H (eag-related), member 2
Kcnk2: potassium channel, subfamily K, member 2
Kcns1: potassium voltage-gated channel, delayed-rectifier, subfamily S, member 1
Kcns3: potassium voltage-gated channel, delayed-rectifier, subfamily S, member 3
Kcnt1: potassium channel, subfamily T, member 1
Kif5a: kinesin family member 5A
Klc1: kinesin light chain 1
Klhl12: kelch-like 12 (Drosophila)
Kpnb1: karyopherin (importin) beta 1
Kras: v-Ki-ras2 Kirsten rat sarcoma viral oncogene homolog
Lama5: laminin, alpha 5
Lancl1: LanC lantibiotic synthetase component C-like 1 (bacterial)
Lars: leucyl-tRNA synthetase
Lbp: lipopolysaccharide binding protein
Lcmt1: leucine carboxyl methyltransferase 1
Letm1: leucine zipper-EF-hand containing transmembrane protein 1
Lgi1: leucine-rich, glioma inactivated 1
Lgr7: relaxin/insulin-like family peptide receptor 1
Lhfpl4: lipoma HMGIC fusion partner-like 4
Lhx3: LIM homeobox 3
Lifr: leukemia inhibitory factor receptor alpha
Lin10: chromosome 16 open reading frame 70
LMO7: LIM domain 7
Lphn1: latrophilin 1
Lphn2: latrophilin 2
Lpin1: lipin 1
Lrpap1: low density lipoprotein receptor-related protein associated protein 1
Lsamp: limbic system-associated membrane protein
Ltbp1: latent transforming growth factor beta binding protein 1
Luzp1: leucine zipper protein 1
Ly6g5b: lymphocyte antigen 6 complex, locus G5B
Lyar: Ly1 antibody reactive homolog (mouse)
LYL1: lymphoblastic leukemia derived sequence 1
Lyst: lysosomal trafficking regulator
Lzic: leucine zipper and CTNNBIP1 domain containing
M6pr: mannose-6-phosphate receptor (cation dependent)
Magi3: membrane associated guanylate kinase, WW and PDZ domain containing 3
Maoa: monoamine oxidase A
Map1lc3a: microtubule-associated protein 1 light chain 3 alpha
Map2: microtubule-associated protein 2
Map3k7ip2: mitogen-activated protein kinase kinase kinase 7 interacting protein 2
Map4: microtubule-associated protein 4
Mapk1: mitogen-activated protein kinase 1
Mapkapk2: mitogen-activated protein kinase-activated protein kinase 2
Mark1: MAP/microtubule affinity-regulating kinase 1
Mark3: MAP/microtubule affinity-regulating kinase 3
Matr3: matrin 3
Mcm7: minichromosome maintenance complex component 7
Mdga2: MAM domain containing glycosylphosphatidylinositol anchor 2
Mdh1: malate dehydrogenase 1, NAD (soluble)
Mecp2: methyl CpG binding protein 2 (Rett syndrome)
Mfap3: microfibrillar-associated protein 3
Mgat1: mannosyl (alpha-1,3-)-glycoprotein beta-1,2-N-acetylglucosaminyltransferase
Mgat5: mannosyl (alpha-1,6-)-glycoprotein beta-1,6-N-acetyl-glucosaminyltransferase
Mid1: midline 1 (Opitz/BBB syndrome)
Mkln1: muskelin 1, intracellular mediator containing kelch motifs
Mkrn2: makorin ring finger protein 2
Mlp: MARCKS-like 1
Mmp10: matrix metallopeptidase 10 (stromelysin 2)
Mnat1: ménage à trois homolog 1, cyclin H assembly factor (Xenopus laevis)
Mpdz: multiple PDZ domain protein
Mpi: mannose phosphate isomerase
Mpo: myeloperoxidase
mpp7: membrane protein, palmitoylated 7 (MAGUK p55 subfamily member 7)
mre11: MRE11 meiotic recombination 11 homolog A (S. cerevisiae)
Mrpl38: mitochondrial ribosomal protein L38
Mrpl9: mitochondrial ribosomal protein L9
Mrps9: mitochondrial ribosomal protein S9
Msn: moesin
Mtap: methylthioadenosine phosphorylase
Mtdh: metadherin
Mtmr3: myotubularin-related protein 3
Mtr: 5-methyltetrahydrofolate-homocysteine methyltransferase
Mtus1: microtubule-associated tumor suppressor 1
Mx1: myxovirus (influenza virus) resistance 1, interferon-inducible protein p78 (mouse)
Mybpc1: myosin binding protein C, slow type
myc: v-myc myelocytomatosis viral oncogene homolog (avian)
Mycbpap: MYCBP associated protein
Myh10: myosin, heavy chain 10, nonmuscle
Myh6: myosin, heavy chain 6, cardiac muscle, alpha
Myh9: myosin, heavy chain 9, nonmuscle
Myo1a: myosin IA
Myo1e: myosin IE
Myo5a: myosin VA (heavy chain 12, myoxin)
Myo5b: myosin VB
Myo7a: myosin VIIA
Myo9a: myosin IXA
Myom1: myomesin 1, 185 kDa
Nab1: NGFI-A binding protein 1 (EGR1 binding protein 1)
Nab2: NGFI-A binding protein 2 (EGR1 binding protein 2)
Naca: nascent polypeptide-associated complex alpha subunit
Naglu: N-acetylglucosaminidase, alpha-
Nap1l3: nucleosome assembly protein 1-like 3
Napepld: N-acyl phosphatidylethanolamine phospholipase D
Nasp: nuclear autoantigenic sperm protein (histone-binding)
Ncam1: neural cell adhesion molecule 1
Ncam2: neural cell adhesion molecule 2
Ncdn: neurochondrin
Ncl: nucleolin
Ncstn: nicastrin
Ndel1: nudE nuclear distribution gene E homolog (A. nidulans)-like 1
Ndufa9: NADH dehydrogenase (ubiquinone) 1 alpha subcomplex, 9, 39 kDa
Ndufc2: NADH dehydrogenase (ubiquinone) 1, subcomplex unknown, 2, 14.5 kDa
Ndufs1: NADH dehydrogenase (ubiquinone) Fe-S protein 1, 75 kDa (NADH-coenzyme Q reductase)
Ndufs6: NADH dehydrogenase (ubiquinone) Fe-S protein 6, 13 kDa (NADH-coenzyme Q reductase)
Ndufs7: NADH dehydrogenase (ubiquinone) Fe-S protein 7, 20 kDa (NADH-coenzyme Q reductase)
Nedd4: neural precursor cell expressed, developmentally down-regulated 4
Nef3: neurofilament, medium polypeptide
Nefh: neurofilament, heavy polypeptide
Negr1: neuronal growth regulator 1
Nell1: NEL-like 1 (chicken)
Neo1: neogenin homolog 1 (chicken)
Nes: nestin
Nexn: nexilin (F actin binding protein)
Nf2: neurofibromin 2 (merlin)
Nfix: nuclear factor I/X (CCAAT-binding transcription factor)
Nfkb1: nuclear factor of kappa light polypeptide gene enhancer in B-cells 1
Ninj1: ninjurin 1
Nlgn2: neuroligin 2
Nlgn3: neuroligin 3
Nme2: non-metastatic cells 2, protein (NM23B) expressed in
Nolc1: nucleolar and coiled-body phosphoprotein 1
Nos1: nitric oxide synthase 1 (neuronal)
Nos3: nitric oxide synthase 3 (endothelial cell)
Notch1: Notch homolog 1, translocation-associated (Drosophila)
Notch2: Notch homolog 2 (Drosophila)
Notch4: Notch homolog 4 (Drosophila)
Npc2: Niemann-Pick disease, type C2
Npdc1: neural proliferation, differentiation and control, 1
Npepps: aminopeptidase puromycin sensitive
Npm1: nucleophosmin (nucleolar phosphoprotein B23, numatrin)
Npr2: natriuretic peptide receptor B/guanylate cyclase B (atrionatriuretic peptide receptor B)
Npvf: neuropeptide VF precursor
Npy5r: neuropeptide Y receptor Y5
Nr1i2: nuclear receptor subfamily 1, group I, member 2
Nr5a2: nuclear receptor subfamily 5, group A, member 2
Nr6a1: nuclear receptor subfamily 6, group A, member 1
Nras: neuroblastoma RAS viral (v-ras) oncogene homolog
Nrbf2: nuclear receptor binding factor 2
Nrp2: neuropilin 2
Nsf: N-ethylmaleimide-sensitive factor
Ntrk2: neurotrophic tyrosine kinase, receptor, type 2
Nucb1: nucleobindin 1
Nucb2: nucleobindin 2
Nudc: nuclear distribution gene C homolog (A. nidulans)
Nup88: nucleoporin 88 kDa
Oas3: 2^'^-5^'^-oligoadenylate synthetase 3, 100 kDa
Obscn: obscurin, cytoskeletal calmodulin and titin-interacting RhoGEF
Ociad1: OCIA domain containing 1
Odc1: ornithine decarboxylase 1
Odf2: outer dense fiber of sperm tails 2
Ogfr: opioid growth factor receptor
Olfm2: olfactomedin 2
Olfm3: olfactomedin 3
Omg: oligodendrocyte myelin glycoprotein
Optn: optineurin
Osbp: oxysterol binding protein
Oxr1: oxidation resistance 1
P4hb: prolyl 4-hydroxylase, beta polypeptide
Pabpc1: poly(A) binding protein, cytoplasmic 1
Pabpc4: poly(A) binding protein, cytoplasmic 4 (inducible form)
Padi2: peptidyl arginine deiminase, type II
Pak3: p21 protein (Cdc42/Rac)-activated kinase 3
Palm: paralemmin
Panx1: pannexin 1
Pard3: par-3 partitioning defective 3 homolog (C. elegans)
Parg: poly (ADP-ribose) glycohydrolase
Park7: Parkinson disease (autosomal recessive, early onset) 7
Pbp: phosphatidylethanolamine binding protein 1
Pcca: propionyl Coenzyme A carboxylase, alpha polypeptide
Pcdh21: protocadherin 21
Pcdha3: protocadherin alpha 3
Pcdhb1: protocadherin beta 1
Pcdhb10: protocadherin beta 10
Pcdhga12: protocadherin gamma subfamily A, 12
Pclo: piccolo (presynaptic cytomatrix protein)
Pcolce: procollagen C-endopeptidase enhancer
Pcsk1n: proprotein convertase subtilisin/kexin type 1 inhibitor
Pcsk5: proprotein convertase subtilisin/kexin type 5
Pcyox1: prenylcysteine oxidase 1
Pdap1: PDGFA associated protein 1
Pdcd4: programmed cell death 4 (neoplastic transformation inhibitor)
Pdcl: phosducin-like
Pde10a: phosphodiesterase 10A
Pde4b: phosphodiesterase 4B, cAMP-specific (phosphodiesterase E4 dunce homolog, Drosophila)
Pdlim7: PDZ and LIM domain 7 (enigma)
Pea15: phosphoprotein enriched in astrocytes 15
Pecr: peroxisomal trans-2-enoyl-CoA reductase
Per1: period homolog 1 (Drosophila)
Per3: period homolog 3 (Drosophila)
Pfkl: phosphofructokinase, liver
PGAP1: post-GPI attachment to proteins 1
Phactr3: phosphatase and actin regulator 3
Phb: prohibitin
Phka1: phosphorylase kinase, alpha 1 (muscle)
Phkg2: phosphorylase kinase, gamma 2 (testis)
Pi4ka: phosphatidylinositol 4-kinase, catalytic, alpha
Picalm: phosphatidylinositol binding clathrin assembly protein
Pik3c3: phosphoinositide-3-kinase, class 3
Pik3cb: phosphoinositide-3-kinase, catalytic, beta polypeptide
Pik3r2: phosphoinositide-3-kinase, regulatory subunit 2 (beta)
Pim1: pim-1 oncogene
Pip5k2a: phosphatidylinositol-5-phosphate 4-kinase, type II, alpha
Pitpnm1: phosphatidylinositol transfer protein, membrane-associated 1
Pkia: protein kinase (cAMP-dependent, catalytic) inhibitor alpha
Pkm2: pyruvate kinase, muscle
Pla2g2a: phospholipase A2, group IIA (platelets, synovial fluid)
Plb1: phospholipase B1
Plcb4: phospholipase C, beta 4
Plcd4: phospholipase C, delta 4
Plec1: plectin 1, intermediate filament binding protein 500kDa
Plk1: polo-like kinase 1 (Drosophila)
Pnkp: polynucleotide kinase 3'-phosphatase
Pnma1: paraneoplastic antigen MA1
Ppara: peroxisome proliferator-activated receptor alpha
Ppargc1b: peroxisome proliferator-activated receptor gamma, coactivator 1 beta
ppfibp2: PTPRF interacting protein, binding protein 2 (liprin beta 2)
Ppia: peptidylprolyl isomerase A (cyclophilin A)
Ppm2c: pyruvate dehydrogenase phosphatase catalytic subunit 1
Ppp1r14b: protein phosphatase 1, regulatory (inhibitor) subunit 14B
Ppp1r1a: protein phosphatase 1, regulatory (inhibitor) subunit 1A
Ppp1r9a: protein phosphatase 1, regulatory (inhibitor) subunit 9A
Ppp2r1a: protein phosphatase 2 (formerly 2A), regulatory subunit A, alpha isoform
Ppp3cc: protein phosphatase 3 (formerly 2B), catalytic subunit, gamma isoform
Pqlc1: PQ loop repeat containing 1
Prdx1: peroxiredoxin 1
Prdx6: peroxiredoxin 6
Prg3: proteoglycan 3
Prkaa2: protein kinase, AMP-activated, alpha 2 catalytic subunit
Prkaca: protein kinase, cAMP-dependent, catalytic, alpha
Prkar1a: protein kinase, cAMP-dependent, regulatory, type I, alpha (tissue specific extinguisher 1)
Prkar2a: protein kinase, cAMP-dependent, regulatory, type II, alpha
Prkcd: protein kinase C, delta
PRKCQ: protein kinase C, theta
Prkwnk1: WNK lysine deficient protein kinase 1
Prlh: prolactin releasing hormone
Prmt3: protein arginine methyltransferase 3
Prpsap2: phosphoribosyl pyrophosphate synthetase-associated protein 2
Prrx2: paired related homeobox 2
Prss12: protease, serine, 12 (neurotrypsin, motopsin)
Prss15: lon peptidase 1, mitochondrial
Prx: periaxin
Psap: prosaposin
Pscd2: cytohesin 2
Pscd3: cytohesin 3
Psg4: pregnancy specific beta-1-glycoprotein 4
Psma1: proteasome (prosome, macropain) subunit, alpha type, 1
Psma2: proteasome (prosome, macropain) subunit, alpha type, 2
Psmc4: proteasome (prosome, macropain) 26S subunit, ATPase, 4
Psme2: proteasome (prosome, macropain) activator subunit 2 (PA28 beta)
Ptbp1: polypyrimidine tract binding protein 1
Ptch1: patched homolog 1 (Drosophila)
Pth: parathyroid hormone
Pthr2: parathyroid hormone 2 receptor
Ptk2b: PTK2B protein tyrosine kinase 2 beta
Ptp4a1: protein tyrosine phosphatase type IVA, member 1
Ptpn11: protein tyrosine phosphatase, non-receptor type 11
Ptpn23: protein tyrosine phosphatase, non-receptor type 23
Ptprj: protein tyrosine phosphatase, receptor type, J
Ptprr: protein tyrosine phosphatase, receptor type, R
Ptprv: protein tyrosine phosphatase, receptor type, V (pseudogene)
Pxn: paxillin
Pygl: phosphorylase, glycogen, liver
Qscn6: quiescin Q6 sulfhydryl oxidase 1
Rab10: RAB10, member RAS oncogene family
Rab14: RAB14, member RAS oncogene family
Rab2: RAB2A, member RAS oncogene family
Rab21: RAB21, member RAS oncogene family
Rab35: RAB35, member RAS oncogene family
Rab3il1: RAB3A interacting protein (rabin3)-like 1
Rab4a: RAB4A, member RAS oncogene family
Rab5a: RAB5A, member RAS oncogene family
Rab7: RAB7A, member RAS oncogene family
Rab9: RAB9A, member RAS oncogene family
Rabac1: Rab acceptor 1 (prenylated)
Rabggta: Rab geranylgeranyltransferase, alpha subunit
Rac1: ras-related C3 botulinum toxin substrate 1 (rho family, small GTP binding protein Rac1)
Rala: v-ral simian leukemia viral oncogene homolog A (ras related)
RalB: v-ral simian leukemia viral oncogene homolog B (ras related; GTP binding protein)
Ralb: v-ral simian leukemia viral oncogene homolog B (ras related; GTP binding protein)
Raly: RNA binding protein, autoantigenic (hnRNP associated with lethal yellow homolog (mouse))
RanGap1: Ran GTPase activating protein 1
Rap2b: RAP2B, member of RAS oncogene family
Rbbp6: retinoblastoma binding protein 6
Rbl2: retinoblastoma-like 2 (p130)
Rbm3: RNA binding motif (RNP1, RRM) protein 3
Rcn1: reticulocalbin 1, EF-hand calcium binding domain
Rcvrn: recoverin
Rdh10: retinol dehydrogenase 10 (all-trans)
Rdx: radixin
Rest: RE1-silencing transcription factor
Rgs4: regulator of G-protein signaling 4
Rgs5: regulator of G-protein signaling 5
Rgs7: regulator of G-protein signaling 7
Rhoa: ras homolog gene family, member A
RhoB: ras homolog gene family, member B
Rhoj: ras homolog gene family, member J
Rims1: regulating synaptic membrane exocytosis 1
Rnasel: ribonuclease L (2^'^,5^'^-oligoisoadenylate synthetase-dependent)
Rnf36: tripartite motif-containing 69
Rnf40: ring finger protein 40
Robo1: roundabout, axon guidance receptor, homolog 1 (Drosophila)
Robo4: roundabout homolog 4, magic roundabout (Drosophila)
Rock1: Rho-associated, coiled-coil containing protein kinase 1
Rock2: Rho-associated, coiled-coil containing protein kinase 2
rora: RAR-related orphan receptor A
Rpe65: retinal pigment epithelium-specific protein 65 kDa
Rpl13: ribosomal protein L13
Rpl6: ribosomal protein L6
Rplp1: ribosomal protein, large, P1
Rpn2: ribophorin II
Rps15: ribosomal protein S15
Rps16: ribosomal protein S16
Rps6ka2: ribosomal protein S6 kinase, 90 kDa, polypeptide 2
Rtcd1: RNA terminal phosphate cyclase domain 1
Rtkn: rhotekin
Rtn1: reticulon 1
Rtn3: reticulon 3
Rtn4: reticulon 4
Rtn4r: reticulon 4 receptor
Samsn1: SAM domain, SH3 domain and nuclear localization signals 1
Sardh: sarcosine dehydrogenase
Sart1: squamous cell carcinoma antigen recognized by T cells
Sbds: Shwachman-Bodian-Diamond syndrome
Sc4mol: sterol-C4-methyl oxidase-like
Sca10: ataxin 10
Scg2: secretogranin II (chromogranin C)
Scg3: secretogranin III
Scn1a: sodium channel, voltage-gated, type I, alpha subunit
Scn2a1: sodium channel, voltage-gated, type II, alpha subunit
Scn5a: sodium channel, voltage-gated, type V, alpha subunit
Scn8a: sodium channel, voltage gated, type VIII, alpha subunit
Scoc: short coiled-coil protein
Scp2: sterol carrier protein 2
SCYB11: chemokine (C-X-C motif) ligand 11
Scye1: aminoacyl tRNA synthetase complex-interacting multifunctional protein 1
Sdfr1: neuroplastin
Sdpr: serum deprivation response
SH3gl1: SH3-domain GRB2-like 1
SH3glb1: SH3-domain GRB2-like endophilin B1
Shank1: SH3 and multiple ankyrin repeat domains 1
Sirpa: signal-regulatory protein alpha
Skiv2l2: superkiller viralicidic activity 2-like 2 (S. cerevisiae)
Slc12a2: solute carrier family 12 (sodium/potassium/chloride transporters), member 2
Slc12a9: solute carrier family 12 (potassium/chloride transporters), member 9
Slc13a3: solute carrier family 13 (sodium-dependent dicarboxylate transporter), member 3
Slc17a6: solute carrier family 17 (sodium-dependent inorganic phosphate cotransporter), member 6
Slc1a1: solute carrier family 1 (neuronal/epithelial high affinity glutamate transporter, system Xag), member 1
Slc26a4: solute carrier family 26, member 4
Slc27a5: solute carrier family 27 (fatty acid transporter), member 5
Slc30a1: solute carrier family 30 (zinc transporter), member 1
Slc44a4: solute carrier family 44, member 4
Slc5a1: solute carrier family 5 (sodium/glucose cotransporter), member 1
Slc6a8: solute carrier family 6 (neurotransmitter transporter, creatine), member 8
Slc8a1: solute carrier family 8 (sodium/calcium exchanger), member 1
Smad4: SMAD family member 4
Smc1l1: structural maintenance of chromosomes 1A
Smoc1: SPARC related modular calcium binding 1
Snai2: snail homolog 2 (Drosophila)
Snap25: synaptosomal-associated protein, 25 kDa
Snap91: synaptosomal-associated protein, 91 kDa homolog (mouse)
Snca: synuclein, alpha (non A4 component of amyloid precursor)
Sncb: synuclein, beta
Snx3: sorting nexin 3
Snx7: sorting nexin 7
sod1: superoxide dismutase 1, soluble
Sod2: superoxide dismutase 2, mitochondrial
Son: SON DNA binding protein
Sord: sorbitol dehydrogenase
Sox5: SRY (sex determining region Y)-box 5
Sp4: Sp4 transcription factor
Sp7: Sp7 transcription factor
Sqle: squalene epoxidase
Srfbp1: serum response factor binding protein 1
Sst: somatostatin
St6gal2: ST6 beta-galactosamide alpha-2,6-sialyltransferase 2
St7l: suppression of tumorigenicity 7 like
Stard3nl: STARD3 N-terminal like
Stat3: signal transducer and activator of transcription 3 (acute-phase response factor)
Stat5a: signal transducer and activator of transcription 5A
Stip1: stress-induced-phosphoprotein 1
Stk2: NIMA (never in mitosis gene a)-related kinase 4
Stk22a: testis-specific serine kinase 1A pseudogene
Stmn1: stathmin 1
Stmn2: stathmin-like 2
Stmn4: stathmin-like 4
Strbp: spermatid perinuclear RNA binding protein
Stx1a: syntaxin 1A (brain)
Stx1b2: syntaxin 1B
Stx5: syntaxin 5
stxbp1: syntaxin binding protein 1
Stxbp3: syntaxin binding protein 3
Sulf1: sulfatase 1
Svop: SV2 related protein homolog (rat)
SYAP1: synapse associated protein 1, SAP47 homolog (Drosophila)
Sycp1: synaptonemal complex protein 1
Sycp2: synaptonemal complex protein 2
Synj2: synaptojanin 2
Syt3: synaptotagmin III
Syt4: synaptotagmin IV
Tacc2: transforming, acidic coiled-coil containing protein 2
Tacc3: transforming, acidic coiled-coil containing protein 3
Tbx2: T-box 2
Tceb3: transcription elongation factor B (SIII), polypeptide 3 (110 kDa, elongin A)
Tcp1: t-complex 1
Tcp11: t-complex 11 homolog (mouse)
Tdg: thymine-DNA glycosylase
Tdrd7: tudor domain containing 7
Tesk2: testis-specific kinase 2
Tfrc: transferrin receptor (p90, CD71)
Tgfb1: transforming growth factor, beta 1
Thrap3: thyroid hormone receptor associated protein 3
Thrb: thyroid hormone receptor, beta (erythroblastic leukemia viral (v-erb-a) oncogene homolog 2, avian)
Thy1: Thy-1 cell surface antigen
Tinag: tubulointerstitial nephritis antigen
Tkt: transketolase
Tle3: transducin-like enhancer of split 3 (E(sp1) homolog, Drosophila)
Tlr5: toll-like receptor 5
Tm4sf8: tetraspanin 3
Tm9sf2: transmembrane 9 superfamily member 2
Tmeff1: transmembrane protein with EGF-like and two follistatin-like domains 1
Tmem17: transmembrane protein 17
Tmlhe: trimethyllysine hydroxylase, epsilon
Tmod2: tropomodulin 2 (neuronal)
Tmpo: thymopoietin
Tnfrsf14: tumor necrosis factor receptor superfamily, member 14 (herpesvirus entry mediator)
Top1: topoisomerase (DNA) I
Tpd52l2: tumor protein D52-like 2
Tpm4: tropomyosin 4
Tra1: heat shock protein 90kDa beta (Grp94), member 1
Traf4: TNF receptor-associated factor 4
Trib3: tribbles homolog 3 (Drosophila)
Trim10: tripartite motif-containing 10
Trim25: tripartite motif-containing 25
Trim50: tripartite motif-containing 50
Trpa1: transient receptor potential cation channel, subfamily A, member 1
TrpV6: transient receptor potential cation channel, subfamily V, member 6
Tsc1: tuberous sclerosis 1
Tsga10: testis specific, 10
Tshr: thyroid stimulating hormone receptor
Ttc1: tetratricopeptide repeat domain 1
Ttn: titin
Tub: tubby homolog (mouse)
Tubb: tubulin, beta
Tubb2: tubulin, beta 2A
Txn: thioredoxin
U2af2: U2 small nuclear RNA auxiliary factor 2
Ubc: ubiquitin C
Ube2l3: ubiquitin-conjugating enzyme E2L 3
Ubqln1: ubiquilin 1
Uchl1: ubiquitin carboxyl-terminal esterase L1 (ubiquitin thiolesterase)
Ugcgl1: UDP-glucose glycoprotein glucosyltransferase 1
Uhrf1: ubiquitin-like with PHD and ring finger domains 1
Unc13: unc-13 homolog B (C. elegans)
Unc13a: unc-13 homolog A (C. elegans)
Unc13d: unc-13 homolog D (C. elegans)
Uqcrfs1: ubiquinol-cytochrome c reductase, Rieske iron-sulfur polypeptide 1
Usp14: ubiquitin specific peptidase 14 (tRNA-guanine transglycosylase)
Usp15: ubiquitin specific peptidase 15
Vamp2: vesicle-associated membrane protein 2 (synaptobrevin 2)
Vangl2: vang-like 2 (van gogh, Drosophila)
Vapa: VAMP (vesicle-associated membrane protein)-associated protein A, 33 kDa
Vapb: VAMP (vesicle-associated membrane protein)-associated protein B and C
Vav1: vav 1 guanine nucleotide exchange factor
Vcp: valosin-containing protein
Vdac2: voltage-dependent anion channel 2
Vegfa: vascular endothelial growth factor A
Vgf: VGF nerve growth factor inducible
Viaat: solute carrier family 32 (GABA vesicular transporter), member 1
Vim: vimentin
Vldlr: very low density lipoprotein receptor
Vps4a: vacuolar protein sorting 4 homolog A (S. cerevisiae)
Vps52: vacuolar protein sorting 52 homolog (S. cerevisiae)
Vps54: vacuolar protein sorting 54 homolog (S. cerevisiae)
Vsnl1: visinin-like 1
Vti1a: vesicle transport through interaction with t-SNAREs homolog 1A (yeast)
Vtn: vitronectin
Wbp11: WW domain binding protein 11
Wbscr1: eukaryotic translation initiation factor 4H
Whsc2: Wolf-Hirschhorn syndrome candidate 2
Wif1: WNT inhibitory factor 1
Wnk4: WNK lysine deficient protein kinase 4
Wrnip1: Werner helicase interacting protein 1
Xpo7: exportin 7
Xrcc5: X-ray repair complementing defective repair in Chinese hamster cells 5
Ybx1: Y box binding protein 1
yes1: v-yes-1 Yamaguchi sarcoma viral oncogene homolog 1
Ywhah: tyrosine 3-monooxygenase/tryptophan 5-monooxygenase activation protein, eta polypeptide
Ywhaq: tyrosine 3-monooxygenase/tryptophan 5-monooxygenase activation protein, theta polypeptide
Zbtb7a: zinc finger and BTB domain containing 7A
Zeb1: zinc finger E-box binding homeobox 1
Zfhx2: zinc finger homeobox 2
Zfp57: zinc finger protein 57 homolog (mouse)
Zmynd19: zinc finger, MYND-type containing 19
Znf219: zinc finger protein 219
Znf291: S-phase cyclin A-associated protein in the ER
Znf292: zinc finger protein 292
Znf382: zinc finger protein 382.

### C.

Cellular signaling pathway clusters of extracted proteins from primary cortical tissue. Specific cellular signaling pathway clusters generated in an un-biased manner using Ingenuity Pathway Analysis (v. 8.5). The relative score generated for the degree of pathway population by proteins from the respective input sets (control or 3xTgAD) is shown in bold. The following are different types of signaling pathways.

#### C.1. ERK/MAPK Signaling (Enrichment Ratio*−log 10(*p*))

Control Protein (**1.32502**) PPP2R2A, CRK, PPP1R14B, DUSP2, PTK2, SHC1, PAK1, PPP1R10, PPP1R7, PIK3CG, STAT1, PRKCA, ETS1, SRC, PAK2, YWHAB, CRKL, PRKAR2A, STAT3, PLA2G4C, FOS, PPP2CB, PPP2R1A, PRKAR2B, PRKCI, PAK3, PPP2R2B, PRKACA, PPP2R5E, ELK1, PPP2R1B, PRKAR1A, PRKCB.

3xTgAD Protein (**0.1537**) PXN, YWHAH, PTK2B, RAC1, PRKAR2A, PLA2G2A, STAT3, MYC, YWHAQ (includes EG:10971), PPP2R1A, PAK3, PIK3C3, PRKCD, PRKACA, PIK3CB, PIK3R2, ELK1, FYN, PRKAR1A.

#### C.2. Inositol Phosphate Metabolism (Enrichment Ratio*−log 10(*p*))

Control Protein (**0.86424**) PDIA3, OCRL, PAK1, PIK3CG, PRKAA2, PLCB1, PLCL1, PI4KA, PMPCA, ATM, GRK4, PRKCQ, PAK2, CDK7, CDK6, PLK1, GRK5, ITPKA, SYNJ2, PIP5K1A, PAK3, SYNJ1, PIP5K1C, GRK6, PIP4K2A, PIP4K2C, CDK2.

3xTgAD Protein (**0.15355**) PLK1, INPP5D, SYNJ2, INPP4A, INPP4B, INPP4B, PAK3, PIM1, PRKCD, PIK3C3, PRKAA2, PIK3CB, PIK3R2, PIP4K2A, PLCD4, PI4KA, ATM.

#### C.3. Wnt/*β*-Catenin Signaling (Enrichment Ratio*−log 10(*p*))

Control Protein (**0.5609**) PPP2R2A, SOX10, CSNK1E, WIF1, WNT7A, TGFB1, SMO, CSNK2A1, CSNK2B, CTNNB1, SRC, SFRP4, GJA1, CSNK1G2, DVL1, FZD9, PPP2CB, CDH2, CDH1, PPP2R1A, PPP2R2B, TLE3, PPP2R5E, UBC, PPP2R1B, WNT5A.

3xTgAD Protein (**0.12772**) CSNK1G1, CSNK1G3, DVL1, ACVR1, APC, MYC, CSNK1E, PPP2R1A, CDH2, CDH1, WIF1, TGFB1, GNAO1, TLE3, UBC, CTNNB1, SOX5 (includes EG:6660).

#### C.4. Calcium Signaling (Enrichment Ratio*−log 10(*p*))

Control Protein (**0.33825**) TRPC2, GRIN2A, TNNI2, GRIA1, RCAN2, CALM2, CHRNB4, TRPC5, TNNT3, RYR3, GRIK1, GRIN2B, PRKAR2A, TRPC4, PPP3CC, TRPC7, CHRNG, PRKAR2B, TPM3, CAMKK1, PRKACA, TNNI1, CHRNB3, CAMKK2, PRKAR1A.

3xTgAD Protein (**0.110875**) RAP2B, MYH10, CALR, MYH6, ATP2B1, PRKAR2A, TPM4, PPP3CC, ATP2B2, HDAC6, TRPV6, HTR3A, CAMKK1, PRKACA, MYH9, SLC8A1, ACTA1, PRKAR1A.

#### C.5. Tight Junction Signaling (Enrichment Ratio*−log 10(*p*))

Control Protein (**0.4147**) TJP2, CDC42, TJP1, PPP2R2A, CLDN19, VAPA, PRKAR2A, PRKCZ, FOS, PPP2CB, PPP2R1A, PRKCI, PRKAR2B, CLDN4, CLDN1, TGFB1, PPP2R2B, PRKACA, PPP2R5E, STX4, CTNNB1, PPP2R1B, PRKAR1A.

3xTgAD Protein (**0.24514**) MYH10, MYH6, CDC42, ACTB, HSF1, CLDN18, VAPA, RAC1, PRKAR2A, MPDZ, PPP2R1A, TGFB1, RHOA, CEBPA, PRKACA, MYH9, CTNNB1, ACTA1, PRKAR1A.

#### C.6. NF-*κ*B Signaling (Enrichment Ratio*−log 10(*p*))

Control Protein (**0.12096**) PRKCQ, EGF, PRKCZ, TLR9, TLR4, TLR5, PIK3CG, TLR6, PRKACA, CSNK2A1, PDGFRA, TLR3, CSNK2B, PRKCB, EGFR.

3xTgAD Protein (**0.0190128**)
*IL1R2, TLR5, BCL10, PIK3C3, PRKACA, IL1B, PIK3CB, PIK3R2, NFKB1, EGFR*.

#### C.7. PTEN Signaling (Enrichment Ratio*−log 10(*p*))

Control Protein (**0.1368**) PTK2, SHC1, CDC42, PIK3CG, CSNK2A1, PDGFRA, CSNK2B, CDKN1B, PRKCZ, FASLG, EGFR.

3xTgAD Protein (**0.1199**) CDC42, YWHAH, RAC1, PIK3CB, PIK3R2, CDKN1B, NFKB1, INPP5D, EGFR, MAGI3.

#### C.8. SAPK/JNK Signaling (Enrichment Ratio*−log 10(*p*))

Control Protein (**0.0544984**) SHC1, CDC42, GADD45A, CRKL, PIK3CG, CRK, ELK1, GNG7.

3xTgAD Protein (**0.0717288**) CDC42, GADD45A, IRS1, PIK3C3, RAC1, PIK3CB, PIK3R2, ELK1, HNRNPK.

#### C.9. PI3K/AKT Signaling (Enrichment Ratio*−log 10(*p*))

Control Protein (**0.15481**) TSC1, PPP2R2A, YWHAB, PRKCZ, PPP2CB, SHC1, PPP2R1A, PIK3CG, TSC2, PPP2R2B, PPP2R5E, CDKN1B, CTNNB1, PPP2R1B.

3xTgAD Protein (**0.19323**) TSC1, JAK1, YWHAH, JAK2, NOS3, INPP5D, YWHAQ (includes EG:10971), MTOR, PPP2R1A, HSP90AB1, PIK3CB, PIK3R2, CDKN1B, CTNNB1.

#### C.10. p38 MAPK Signaling (Enrichment Ratio*−log 10(*p*))

Control Protein (**0**)
 

3xTgAD Protein (**0.0630702**) IL1R2, MYC, TGFB1, MAP3K7IP2, IL1B, PLA2G2A, EEF2K, MAPKAPK2, ELK1.

#### C.11. p53 Signaling (Enrichment Ratio*−log 10(*p*))

Control Protein (**0.037996**) GADD45A, FASN, PIK3CG, SNAI2, CTNNB1, CDK2, ATM, SERPINE2.

3xTgAD Protein (**0.12075**) CCND2, GADD45A, PIK3C3, SNAI2, CABC1, PIK3CB, PIK3R2, BAX, CTNNB1, ATM.

#### C.12. JAK/Stat Signaling (Enrichment Ratio*−log 10(*p*))

Control Protein (**0.051714**) SHC1, SOCS1, PTPN11, PIK3CG, STAT3, STAT1.

3xTgAD Protein (**0.25857**) STAT5A, MTOR, JAK1, PTPN11, PIK3C3, PIK3CB, STAT3, PIK3R2, JAK2.

### D.

Neuronal function pathway clusters of extracted proteins from primary cortical tissue. Specific neuronal function pathway clusters generated in an un-biased manner using Ingenuity Pathway Analysis (v. 8.5). The relative score generated for the degree of pathway population by proteins from the respective input sets (control or 3xTgAD) is shown in bold. The following are different types of signaling pathways.

#### D.1. Synaptic Long-Term Potentiation (Enrichment Ratio*−log 10(*p*))

Control Protein (**1.27872**) GRIN2B, GRIN2A, PRKCQ, PPP1R1A, GRIA1, PRKAR2A, CALM2, CACNA1C, PPP3CC, GRM4, PPP1R14B, PRKCZ, GRM5, PRKCI, PRKAR2B, PPP1R10, PPP1R7, PPP1R14D, PRKACA, PLCB1, PRKD1, PRKCA, PRKAR1A, PRKCB

3xTgAD Protein (**0**)
 

#### D.2. Axonal Guidance Signaling (Enrichment Ratio*−log 10(*p*))

Control Protein (**0.69525**) NOS1, GUCY1B2, PRKCQ, GUCY1A3, GUCY2D, PPP2R2A, GRID2, GRIA1, GRM4, PRKCZ, GRM5, PPP2CB, PPP2R1A, PRKCI, PPP2R2B, RYR3, GUCY1A2, PLCB1, PPP2R5E, PPP2R1B, PRKD1, PRKCA, PRKCB.

3xTgAD Protein (**0.1918**) NOS1, GUCY2D, GNAI1, PLA2G2A, GNAZ, NOS3, PRDX6, GNAI2, PPP2R1A, PRKCD, GNAO1, IGF1R, GUCY2F, ADCY8, NPR2.

#### D.3. Synaptic Long-Term Depression (Enrichment Ratio*−log 10(*p*))

Control Protein (**0.47428**) NOS1, GUCY1B2, PRKCQ, GUCY1A3, GUCY2D, PPP2R2A, GRID2, GRIA1, GRM4, PRKCZ, GRM5, PPP2CB, PPP2R1A, PRKCI, PPP2R2B, RYR3, GUCY1A2, PLCB1, PPP2R5E, PPP2R1B, PRKD1, PRKCA, PRKCB.

3xTgAD Protein (**0.11112**) NOS1, GUCY2D, GNAI1, PLA2G2A, GNAZ, NOS3, PRDX6, GNAI2, PPP2R1A, PRKCD, GNAO1, IGF1R, GUCY2F, ADCY8, NPR2.

#### D.4. Parkinson's Signaling (Enrichment Ratio*−log 10(*p*))

Control Protein (**0.168256**) UCHL1, PARK7, SNCA.

3xTgAD Protein (**0.18656**) UCHL1, PARK7, SNCA.

#### D.5. Huntington's Disease Signaling (Enrichment Ratio*−log 10(*p*))

Control Protein (**0.20048**) EGF, GNG7, PRKCZ, TGM2, CTSD, SHC1, PIK3CG, VAMP3, PLCB1, PRKD1, PRKCA, EGFR, SDHA, GRIN2B, PRKCQ, YKT6, SH3GL3, STX1A, SNAP25, TAF9B, GRM5, PRKCI, UBC, GOSR2, SNCA, PRKCB.

3xTgAD Protein (**0.36179**) VTI1A, HSPA14, REST, GNB2L1, HDAC6, NSF, MTOR, PIK3C3, IGF1R, PIK3R2, EGFR, CAPN5, CAPN6, CLTC, BAX, STX1A, SNAP25, DNM1, DNAJC5, ATP5B, CAPN1, PRKCD, HAP1, DCTN1, PIK3CB, UBC, SNCA, CASP7.

#### D.6. Regulation of Actin-Based Motility by Rho (Enrichment Ratio*−log 10(*p*))

Control Protein (**0.1476**) PIP5K1A, WIPF1, PAK1, PAK2, CDC42, CFL1, PAK3, PIP5K1C, PIP4K2A, PIP4K2C, PI4KA.

3xTgAD Protein (**0.3243**) CDC42, CFL1, ACTB, RAC1, RHOJ, ROCK1, PAK3, RHOB, RHOA, ARHGDIA, PIP4K2A, ACTA1, PI4KA.

#### D.7. Amyotrophic Lateral Sclerosis Signaling (Enrichment Ratio*−log 10(*p*))

Control Protein (**0.23184**) NOS1, GRIN2B, GRIN2A, SOD1, GRIA1, PGF, VEGFA, PAK1, GRIK4, PIK3CG, CAT, GLUL, SSR4.

3xTgAD Protein (**0.55955**) NOS1, CAPN5, CAPN6, SOD1, RAB5A, RAC1, NEFH, BAX, CCS, VEGFA, CAPN1, PIK3C3, NEFM, PIK3CB, PIK3R2, CASP7.

#### D.8. Actin Cytoskeleton Signaling (Enrichment Ratio*−log 10(*p*))

Control Protein (**0.0763861**) PAK2, CFL1, CDC42, CRKL, EGF, CRK, TTN, PTK2, SHC1, PAK1, PIP5K1A, PAK3, PIP5K1C, FGF18, PIK3CG, EZR, FGF23, PIP4K2A, PIP4K2C, GIT1.

3xTgAD Protein (**0.44928**) MYH10, MYH6, CDC42, ROCK2, PIK3C3, EZR, PIK3R2, LBP, ACTA1, PXN, ARHGEF12, CFL1, ACTB, RAC1, RDX, APC, TTN, ROCK1, PAK3, RHOA, ARHGEF6, MYH9, VAV1, PIK3CB, ACTN4, PIP4K2A, MSN.

#### D.9. Amyloid Processing (Enrichment Ratio*−log 10(*p*))

Control Protein (**0.20636**) CSNK1E, PRKAR2B, CSNK2A, PRKAR2A, PRKACA, NCSTN, CSNK2B, PRKAR1A.

3xTgAD Protein (**0.66144**) CSNK1E, CAPN5, CAPN6, CAPN1, MARK1, PRKAR2A, PRKACA, NCSTN, BACE1, APP, PRKAR1A.

### E.

Energy regulation/Metabolism pathway clusters of extracted proteins from primary cortical tissue. Specific energy regulation/metabolism pathway clusters generated in an un-biased manner using Ingenuity Pathway Analysis (v. 8.5). The relative score generated for the degree of pathway population by proteins from the respective input sets (control or 3xTgAD) is shown in bold. The following are different types of signaling pathways.

#### E.1. Amino Sugars Metabolism (Enrichment Ratio*−log 10(*p*))

Control Protein (**0.61425**) FMO3, PDE7A, PDE10A, GM2A, CYB5R1, PDE4A, PDE4B, PDE1A, PDE4D, GFPT2, PDE1C, PDE7B, GALK1, CYB5R3.

3xTgAD Protein (**0.0471409**) HK1, CYB5R4, PDE10A, GALK1, CYB5R3, PDE4B.

#### E.2. Pentose Phosphate Pathway (Enrichment Ratio*−log 10(*p*))

Control Protein (**0.48608**) GPI, PGD, TKT, TALDO1, PRPSAP1, PGM1, PRPSAP2, PFKP, PFKL, PDHB, PFKM.

3xTgAD Protein (**0.0245154**) GPI, TKT, PRPSAP2, PFKL.

#### E.3. Glutamate Metabolism (Enrichment Ratio*−log 10(*p*))

Control Protein (**0.31165**) FMO3, GPT, GLUL, GCLC, GLUD1, GOT1, GOT2, GFPT2, EPRS.

3xTgAD Protein (**0**)
 

#### E.4. Pantothenate and CoA Biosynthesis (Enrichment Ratio*−log 10(*p*))

Control Protein (**0.34188**) ENPP3, CRMP1, BCAT1, DPYD, DPYSL3, ENPP5, ENPP2.

3xTgAD Protein (**0.0348908**) CRMP1, DPYD, ENPP2.

#### E.5. Glycolysis/Gluconeogenesis (Enrichment Ratio*−log 10(*p*))

Control Protein (**0.28363**) PKM2, PGK1, ENO3, ENO2, GAPDH (includes EG:2597), PGM1, ALDH1L1, PFKP, PFKL, PDHB, PFKM, GPI, GALK1, DLAT, PGAM1, DLD.

3xTgAD Protein (**0.0610449**) PKM2, HK1, ADH1A, GPI, ALDH1A3, DLAT, GALK1, ENO2, PFKL, ACSL1.

#### E.6. Citrate Cycle (Enrichment Ratio*−log 10(*p*))

Control Protein (**0.1581**) SDHA, PC, CS, SUCLG1, DLD, PCK1.

3xTgAD Protein (**0**)
 

#### E.7. Galactose Metabolism

Control Protein (**0.104958**) GLA, GALT, GALK1, GAA, PGM1, PFKP, PFKL, PFKM.

3xTgAD Protein (**0.0486688**) HK1, GLA, GALK1, GAA, PFKL, AKR1B1.

#### E.8. Glycosphingolipid Biosynthesis—Globoseries (Enrichment Ratio*−log 10(*p*))

Control Protein (**0.0411384**) GLA, ST3GAL1, GM2A.

3xTgAD Protein (**0**)
 

#### E.9. Glycosphingolipid Biosynthesis—Ganglioseries (Enrichment Ratio*−log 10(*p*))

Control Protein (**0.008832**) ST3GAL1, GM2A.

3xTgAD Protein (**0**)
 

#### E.10. N-Glycan Biosynthesis (Enrichment Ratio*−log 10(*p*))

Control Protein (**0**)
 

3xTgAD Protein (**0.015272**) RPN2, ST6GAL2, MGAT5, MGAT1.

#### E.11. Pentose and Glucuronate Interconversions (Enrichment Ratio*−log 10(*p*))

Control Protein (**0**)
 

3xTgAD Protein (**0.0184464**) UCHL1, B3GAT1, HPSE, DCXR, AKR1B1.

#### E.12. Fatty Acid Elongation in Mitochondria (Enrichment Ratio*−log 10(*p*))

Control Protein (**0**)
 

3xTgAD Protein (**0.0206016**) ECHS1, PECR.

#### E.13. Glycerophospholipid Metabolism (Enrichment Ratio*−log 10(*p*))

Control Protein (**0.0232106**) GPAM, PCYT1B, PLD3, CDS1, PDIA3, GNPAT, DGKB, DGKG, PLCB1, PLCL1.

3xTgAD Protein (**0.048204**) AGPAT4, CDS1, GNPAT, CHKA, AGPAT1, DGKG, PLA2G2A, CLCF1, PHKA1, PLCD4, PRDX6.

#### E.14. Fatty Acid Biosynthesis (Enrichment Ratio*−log 10(*p*))

Control Protein (**0**)
 

3xTgAD Protein (**0.0329256**) ACACB, ACACA.

#### E.15. Purine Metabolism (Enrichment Ratio*−log 10(*p*))

Control Protein (**0.08616**) ENTPD8, PDE7A, PDE4A, DNTT, PDE1A, PDE4D, PSMC5, PDE7B, VCP, DHX16, ENPP2, PRPSAP2, PKM2, TJP2, GUCY1B2, GUCY1A3, GUCY2D, PDE10A, ENPP5, PDE4B, PDE1C, ENPP3, PSMC1, ENTPD2, PRPSAP1, GUCY1A2, PDE5A, DDX1, GMPR2, PSMC3.

3xTgAD Protein (**0.124932**) MYH6, ENTPD8, NME2, DNTT, WRNIP1, ABCC1, VCP, KATNA1, ATP5H (includes EG:10476), ENPP2, PRPSAP2, ADCY8, ATP5F1, ATP5I, ATP5J, PKM2, ATP1B1, GUCY2D, PDE10A, AMPD1, ATP5A1, AK3, PSMC4, PDE4B, DDX19B, ATP5B, GUCY2F, MYH9, DDX1, NPR2.

#### E.16. Pyruvate Metabolism (Enrichment Ratio*−log 10(*p*))

Control Protein (**0.0214935**) PKM2, PC, DLAT, DLD, PCK1, PDHB, GLO1.

3xTgAD Protein (**0.070173**) PKM2, ACACB, ALDH1A3, DLAT, ACAT1, ACACA, MDH1, AKR1B1, ACSL1.

#### E.17. Butanoate Metabolism (Enrichment Ratio*−log 10(*p*))

Control Protein (**0**)
 

3xTgAD Protein (**0.0523452**) ECHS1, ALDH1A3, AACS, ACAT1, MYO5B, DCXR, HMGCS1.

#### E.18. Synthesis and Degradation of Ketone Bodies (Enrichment Ratio*−log 10(*p*))

Control Protein (**0**)
 

3xTgAD Protein (**0.071505**) ACAT1, HMGCS1.

### F.

Cellular stress/damage pathway clusters of extracted proteins from primary cortical tissue. Specific energy cellular stress/damage pathway clusters generated in an un-biased manner using Ingenuity Pathway Analysis (v. 8.5). The relative score generated for the degree of pathway population by proteins from the respective input sets (control or 3xTgAD) is shown in bold. The following are different types of signaling pathways.

#### F.1. NRF2-mediated Oxidative Stress Response (Enrichment Ratio*−log 10(*p*))

Control Protein (**0.44064**) PRDX1, PPIB, GCLC, PRKCZ, GSTM2, SOD2, PIK3CG, VCP, DNAJA2, TXN, FKBP5, PRKD1, CBR1, PRKCA, GSTK1, SOD1, PRKCQ, TXNRD1, GSTO1, FOS, PRKCI, ERP29, STIP1, CAT, PRKCB, EPHX1.

3xTgAD Protein (**0.23751**) USP14, SOD1, PRDX1, ACTB, GSTM3 (includes EG:2947), JUNB, DNAJC5, GSTM2, SOD2, ERP29, STIP1, PIK3C3, PRKCD, ABCC1, VCP, PIK3CB, FOSL1, PIK3R2, TXN, EIF2AK3, ACTA1.

#### F.2. Ceramide Signaling (Enrichment Ratio*−log 10(*p*))

Control Protein (**0.15748**) CTSD, PPP2CB, FOS, PPP2R1A, PPP2R2A, PIK3CG, PPP2R2B, PPP2R5E, PPP2R1B.

3xTgAD Protein (**0**)
 

#### F.3. Glutathione Metabolism (Enrichment Ratio*−log 10(*p*))

Control Protein (**0.094285**) OPLAH, PGD, GSTM2, GGT5, GCLC, GGT1, GSTO1, GSTK1, GGT7.

3xTgAD Protein (**0**)
 

#### F.4. LPS/IL-1 Mediated Inhibition of RXR Function (Enrichment Ratio*−log 10(*p*))

Control Protein (**0.19071**) SULT1C2, FMO3, CPT1A, CHST7, ALDH1L1, NR1H3, GSTO1, SULT4A1, GSTM2, NR1I2, SREBF1, SULT1A1, CAT, CPT2, FABP4, XPO1, FABP1, FABP7, SULT1E1, FABP3, GSTK1, TLR4, FABP2.

3xTgAD Protein (**0.13872**) PPARA, GSTM3 (includes EG:2947), ABCA1, IL1R2, ABCC2, SLC27A5, GSTM2, ALDH1A3, NR1I2, CPT2, ACSL4, FABP1, IL1B, NR5A2, CHST10, LBP, ABCC4, HMGCS1, ACSL1, MAOA.

#### F.5. Fatty Acid Elongation in Mitochondria (Enrichment Ratio*−log 10(*p*))

Control Protein (**0**)
 

3xTgAD Protein (**0.0206016**) ECHS1, PECR

#### F.6. Endoplasmic Reticulum Stress Pathway (Enrichment Ratio*−log 10(*p*))

Control Protein (**0**)
 

3xTgAD Protein (**0.047841**) EIF2AK3, CASP7.

#### F.7. Apoptosis Signaling (Enrichment Ratio*−log 10(*p*))

Control Protein (**0**)
 

3xTgAD Protein (**0.059156**) ACIN1, ROCK1, CAPN5, CAPN6, CAPN1, BAX, CASP7, PARP1.

#### F.8. Hypoxia Signaling (Enrichment Ratio*−log 10(*p*))

Control protein (**0**)
 

3xTgAD Protein (**0.111983**) VEGFA, P4HB, HSP90AB1, UBE2L3, NOS3, UBC, ARNT, ATM.

#### F.9. PPAR*α*/RXR*α* Activation (Enrichment Ratio*−log 10(*p*))

Control Protein (**0.0315615**) PDIA3, PRKAR2A, NR2C2, SHC1, PRKAR2B, TGFB1, FASN, PRKACA, PLCB1, GOT2, PLCL1, PRKCB, PRKCA, PRKAR1A.

3xTgAD Protein (**0.14586**) PPARA, ACVR1, PRKAR2A, CD36, IL6, JAK2, ABCA1, IL1R2, ACADL, HSP90AB1, TGFB1, IRS1, PRKACA, SMAD4, IL1B, ADCY8, PLCD4, PRKAR1A.

### G.

Receptor signaling pathway clusters of extracted proteins from primary cortical tissue. Specific receptor signaling pathway clusters generated in an un-biased manner using Ingenuity Pathway Analysis (v. 8.5). The relative score generated for the degree of pathway population by proteins from the respective input sets (control or 3xTgAD) is shown in bold. The following are different types of signaling pathways.

#### G.1. Dopamine Receptor Signaling

Control Protein (**0.65824**) PPP2R2A, PRKAR2A, PPP1R14B, PPP2CB, PPP2R1A, PRKAR2B, PPP1R10, PPP1R14D, PPP1R7, PPP2R2B, PRKACA, FREQ, PPP2R5E, PPP2R1B, PRKAR1A, CALY.

3xTgAD Protein (**0.071199**) PPP2R1A, FREQ, PRKAR2A, PRKACA, ADCY8, CALY, MAOA, PRKAR1A.

#### G.2. PDGF Signaling

Control Protein (**0.73892**) SRC, CRKL, CRK, STAT3, FOS, SHC1, PIK3CG, CSNK2A1, PDGFRA, CAV1, CSNK2B, ELK1, STAT1, PRKCB, PRKCA.

3xTgAD Protein (**0.15738**) MYC, JAK1, PIK3C3, STAT3, PIK3R2, JAK2, ELK1, INPP5D.

#### G.3. EGF Signaling

Control Protein (**0.7956**) SHC1, FOS, PIK3CG, CSNK2A1, EGF, CSNK2B, STAT3, STAT1, ELK1, PRKCA, EGFR.

3xTgAD Protein (**0.22499**) JAK1, PIK3C3, PIK3CB, STAT3, PIK3R2, ELK1, EGFR.

#### G.4. Neuregulin Signaling

Control Protein (**0.68068**) SRC, PRKCQ, CRKL, EGF, CRK, ERBB3, PRKCZ, SHC1, PRKCI, PTPN11, ERBB4, CDKN1B, ELK1, PRKD1, PRKCB, EGFR, PRKCA.

3xTgAD Protein (**0.1276**) MYC, STAT5A, MTOR, PTPN11, HSP90AB1, PRKCD, PIK3R2, CDKN1B, ELK1, EGFR.

#### G.5. Glutamate Receptor Signaling

Control Protein (**0.53342**) GRM5, GRIN2B, GRIN2A, GRIK4, GRIA1, GRID2, CALM2, GLUL, GRIP1, GRM4, GNG7, GRIK1.

3xTgAD Protein (**0**)
 

#### G.6. Sonic Hedgehog Signaling

Control Protein (**0.73272**) SHH, CCNB1, PRKAR2B, PRKAR2A, PRKACA, SMO, GLI1, PRKAR1A.

3xTgAD Protein (**0.20769**) ADRBK1, PTCH1, PRKAR2A, PRKACA, PRKAR1A.

#### G.7. TR/RXR Activation

Control Protein (**0.5481**) UCP2, RCAN2, UCP1, THRA, GRIP1, PFKP, PCK1, KLF9, SREBF1, DIO1, FASN, PIK3CG, STRBP, THRB, FGA, SYT12.

3xTgAD Protein (**0.0321142**) MTOR, PIK3C3, STRBP, ACACA, PIK3CB, PIK3R2, THRB.

#### G.8. Ephrin Receptor Signaling

Control Protein (**0.62472**) GRIN2A, CDC42, EGF, CRK, GNG7, PGF, VEGFA, PTK2, SHC1, PAK1, SDC2, PIK3CG, EFNB1, EPHA7, GRIN2B, SRC, PAK2, CFL1, CRKL, EPHA3, STAT3, WIPF1, SDCBP, PTPN11, PAK3, EPHA5, EPHB3.

3xTgAD Protein (**0.21037**) FYN, PXN, CFL1, CDC42, KALRN, GNB2L1, GNAI1, RAC1, JAK2, STAT3, GNAZ, ROCK1, VEGFA, GNAI2, ROCK2, ABI1, PTPN11, PAK3, RHOA, GNAO1.

#### G.9. G-Protein Coupled Receptor Signaling

Control Protein (**0.3808**) PDE7A, PDE4A, PDE1A, PDE4D, SHC1, SYNGAP1, PDE7B, PIK3CG, PLCB1, PRKCA, SRC, GRK4, EDNRB, PDE10A, PRKAR2A, PDE4B, STAT3, GRM4, PDE1C, CHRM5, GRM5, OPRD1, PRKAR2B, PRKACA, PRKCB, PRKAR1A.

3xTgAD Protein (**0.13938**) FYN, PTK2B, HTR4, ADRBK1, PDE10A, RGS7, PRKAR2A, GNAI1, RGS4, STAT3, PDE4B, GNAI2, HTR2C, PIK3C3, GNAO1, PRKACA, PIK3CB, PIK3R2, ADCY8, PRKAR1A.

#### G.10. Chemokine Signaling

Control Protein (**0.23961**) PTK2, NOX1, SRC, FOS, CCL4, CFL1, PIK3CG, CALM2, PLCB1, PRKCA, PRKCB.

3xTgAD Protein (**0.03232**) ROCK2, GNAI2, CFL1, PTK2B, RHOA, GNAI1.

#### G.11. Ceramide Signaling

Control Protein (**0.15748**) CTSD, PPP2CB, FOS, PPP2R1A, PPP2R2A, PIK3CG, PPP2R2B, PPP2R5E, PPP2R1B, PRKCZ.

3xTgAD protein (**0**)
 

#### G.12. FGF (Fibroblast Growth Factor) Signaling

Control Protein (**0.17423**) FGFR3, PTPN11, FGF18, CRKL, PIK3CG, FGFR1, FGF23, FGFR2, CRK, STAT3, PRKCA.

3xTgAD Protein (**0.0667352**) PTPN11, PIK3C3, HGF, RAC1, PIK3CB, STAT3, PIK3R2, MAPKAPK2.

#### G.13. Toll-like Receptor Signaling

Control Protein (**0.1644**) TLR4, FOS, TLR5, TLR6, TLR3, ELK1, TLR9.

3xTgAD Protein (**0.065562**) PPARA, TLR5, MAP3K7IP2, LBP, ELK1.

#### G.14. Nitric Oxide Signaling

Control Protein (**0.41157**) GUCY1B2, GUCY1A3, GUCY2D, CALM2, PRKAR2A, PGF, VEGFA, PRKAR2B, PIK3CG, GUCY1A2, CAV1, PRKACA, PRKAR1A.

3xTgAD Protein (**0.36801**) VEGFA, HSP90AB1, GUCY2D, PRKCD, PIK3C3, GUCY2F, PRKAR2A, PRKACA, PIK3CB, PIK3R2, NOS3, PRKAR1A.

#### G.15. cAMP-Mediated Signaling

Control Protein (**0.39295**) GRK4, SRC, CNGA4, PDE7A, PDE10A, PRKAR2A, CALM2, PDE4A, PPP3CC, STAT3, PDE4B, GRM4, PDE1A, PDE4D, CNGA1, PDE1C, CHRM5, OPRD1, PRKAR2B, PDE7B, PRKACA, PKIA, PRKAR1A.

3xTgAD Protein (**0.40848**) AKAP12, HTR4, ADRBK1, PDE10A, RGS7, PRKAR2A, GNAI1, RGS4, AKAP3, PPP3CC, STAT3, PDE4B, CNGA1, GNAI2, AKAP4, GNAO1, PRKACA, CNGB1, PKIA, ADCY8, AKAP9, PRKAR1A.

#### G.16. TGF-*β* Signaling

Control protein (**0**)
 

3xTgAD Protein (**0.0236421**) TGFB1, ACVR1, SMAD4, HNF4A, UBC, INHBC.

#### G.17. Neurotrophin/TRK Signaling

Control Protein (**0.016851**) SHC1, FOS, CDC42, PTPN11, PIK3CG.

3xTgAD Protein (**0.0440592**) NTRK2, CDC42, PTPN11, PIK3C3, PIK3CB, PIK3R2.

#### G.18. IGF-1 Signaling

Control Protein (**0.49715**) PRKAR2A, PTK2, SHC1, FOS, PRKAR2B, PTPN11, PIK3CG, PRKACA, CSNK2A1, CSNK2B, ELK1, PRKAR1A.

3xTgAD Protein (**0.52722**) PXN, YWHAH, PRKAR2A, NEDD4, YWHAQ, PTPN11, IRS1, PIK3C3, PRKACA, IGF1R, IRS2, PIK3CB, PIK3R2, ELK1, PRKAR1A.

#### G.19. Serotonin Receptor Signaling

Control protein (**0**)
 

3xTgAD Protein (**0.059769**) HTR2C, HTR4, HTR3A, MAOA.

#### G.20. Glucocorticoid Receptor Signaling

Control protein (**0**)
 

3xTgAD Protein (**0.066**) STAT5A, JAK1, YWHAH, HSPA14, RAC1, IL6, PPP3CC, STAT3, JAK2, NFKB1, MNAT1, IL1R2, HSP90AB1, TGFB1, PIK3C3, CEBPA, PRKACA, SMAD4, IL1B, PIK3CB, PIK3R2, ELK1.

#### G.21. Aryl Hydrocarbon Receptor Signaling

Control Protein (**0.1218**) SRC, CDK6, ALDH1L1, GSTO1, TGM2, CTSD, FOS, GSTM2, TGFB1, FASN, CDKN1B, ESR2, CDK2, FASLG, ATM, GSTK1.

3xTgAD Protein (**0.31125**) CYP1A1, NFIX, GSTM3, IL6, BAX, NFKB1, ARNT, MYC, AHRR, GSTM2, CCND2, HSP90AB1, TGFB1, ALDH1A3, IL1B, CDKN1B, AHR, MCM7, ATM.

#### G.22. Insulin Receptor Signaling

Control Protein (**0.28288**) TSC1, TRIP10, CRKL, PRKAR2A, CRK, VAMP2, PPP1R14B, PRKCZ, SHC1, PRKCI, PPP1R10, PTPN11, PIK3CG, TSC2, PRKACA.

3xTgAD Protein (**0.5056**) TSC1, FYN, JAK1, PRKAR2A, JAK2, VAMP2, INPP5D, MTOR, PTPN11, IRS1, PIK3C3, PRKACA, EIF2B1, PIK3CB, IRS2, PIK3R2, PRKAR1A.

#### G.23. Notch Signaling

Control Protein (**0.0437248**) DLL1, CNTN1, NCSTN, DLL3.

3xTgAD Protein (**0.30609**) NOTCH4, DLL1, NOTCH2, NCSTN, JAG1, NOTCH1, HEY1.

#### G.24. GABA Receptor Signaling

Control Protein (**0.099214**) GABRG2, UBQLN1, GABBR1, GABARAP, UBC, GABRA3.

3xTgAD Protein (**0.4216**) DNM1, NSF, SLC32A1, UBQLN1, GABBR1, MYO5B, GABRE, UBC, GABRA3.

#### G.25. VEGF Signaling

Control Protein (**0.0252652**) VEGFA, PTK2, SHC1, PIK3CG, PGF, PRKCA, PRKCB.

3xTgAD Protein (**0.53464**) EIF2S2, PXN, PTK2B, ACTB, NOS3, ARNT, ROCK2, VEGFA, ROCK1, PIK3C3, EIF2B1, PIK3CB, PIK3R2, ACTN4, ACTA1.

#### G.26. Integrin Signaling

Control protein (**0**)
 

3xTgAD Protein (**0.54604**) RAP2B, FYN, RALA, CDC42, TSPAN3, ARF6, RHOB, PIK3C3, ARF4, PIK3R2, ACTA1, CAPN5, CAPN6, PXN, ACTB, RALB, RAC1, ITGA6, RHOJ.

### H.

Heatmap Protein Position Key The individual position numbers are correlated to the specific multidimensional proteins represented by colored blocks in Figure 6. The following are the symbols of the proteins (the numbers between brackets refer to the position).

1. adcyap1: adenylate cyclase activating polypeptide 1 (pituitary)
2. adcyap1r1: adenylate cyclase activating polypeptide 1 (pituitary) receptor type I
3. hap1: huntingtin-associated protein 1
4. htr4: 5-hydroxytryptamine (serotonin) receptor 4
5. ntrk2: neurotrophic tyrosine kinase, receptor, type 2
6. Kalrn: kalirin, RhoGEF kinase
7. klc1: kinesin light chain 1
8. adrbk1: adrenergic, beta, receptor kinase 1
9. agrn: agrin
10. apod: apolipoprotein D
11. app: amyloid beta (A4) precursor protein
12. arhgef11: Rho guanine nucleotide exchange factor (GEF) 11
13. arpp19: cAMP-regulated phosphoprotein, 19 kDa
14. bace1: beta-site APP-cleaving enzyme 1
15. ccs: copper chaperone for superoxide dismutase
16. chgb: chromogranin B (secretogranin 1)
17. flot1: flotillin 1
18. flot2: flotillin 2
19. gnb2l1: guanine nucleotide binding protein (G protein), beta polypeptide 2-like 1
20. hyou1: hypoxia up-regulated 1
21. lphn1: latrophilin 1
22. lphn2: latrophilin 2
23. magi3: membrane associated guanylate kinase, WW and PDZ domain containing 3
24. nefh: neurofilament, heavy polypeptide
25. prdx6: peroxiredoxin 6
26. pscd2: cytohesin 2
27. rtn3: reticulon 3
28. rtn4r: reticulon 4 receptor
29. slc44a4: solute carrier family 44, member 4
30. stxbp1: syntaxin binding protein 1
31. abca1: ATP-binding cassette, sub-family A (ABC1), member 1
32. abi1: abl-interactor 1
33. adcy1: adenylate cyclase 1 (brain)
34. adcy5: adenylate cyclase 5
35. akap9: A kinase (PRKA) anchor protein 9
36. aldh1a3: aldehyde dehydrogenase 1 family, member A3
37. alox5: arachidonate 5-lipoxygenase
38. arf6: ADP-ribosylation factor 6
39. arhgef12: Rho guanine nucleotide exchange factor (GEF) 12
40. atcay: ataxia, cerebellar, Cayman type
41. atp2b1: ATPase, Ca++ transporting, plasma membrane 1
42. atxn3: ataxin 3
43. begain: brain-enriched guanylate kinase-associated homolog (rat)
44. cabc1: chaperone, ABC1 activity of bc1 complex homolog (S. pombe)
45. capn1: calpain 1, (mu/l) large subunit
46. caskin1: CASK interacting protein 1
47. casp7: caspase 7, apoptosis-related cysteine peptidase
48. cd36: CD36 molecule (thrombospondin receptor)
49. cfd: complement factor D (adipsin)
50. clu: clusterin
51. cpa2: carboxypeptidase A2 (pancreatic)
52. cript: cysteine-rich PDZ-binding protein
53. dpp6: dipeptidyl-peptidase 6
54. espn: espin
55. fdps: farnesyl diphosphate synthase
56. filip1: filamin A interacting protein 1
57. gna11: guanine nucleotide binding protein (G protein), alpha 11 (Gq class)
58. gnai2: guanine nucleotide binding protein (G protein), alpha inhibiting activity polypeptide 2
59. gnao1: guanine nucleotide binding protein (G protein), alpha activating activity polypeptide O
60. gnaq: guanine nucleotide binding protein (G protein), q polypeptide
61. gnaz: guanine nucleotide binding protein (G protein), alpha z polypeptide
62. gpr56: G protein-coupled receptor 56
63. hip1r: huntingtin interacting protein 1 related
64. hmgcr: 3-hydroxy-3-methylglutaryl-Coenzyme A reductase
65. hsf1: heat shock transcription factor 1
66. htr2c: 5-hydroxytryptamine (serotonin) receptor 2C
67. itm2c: integral membrane protein 2C
68. itpr3: inositol 1,4,5-triphosphate receptor, type 3
69. lsamp: limbic system-associated membrane protein
70. maoa: monoamine oxidase A
71. map1lc3a: microtubule-associated protein 1 light chain 3 alpha
72. mapkapk2: mitogen-activated protein kinase-activated protein kinase 2
73. mark1: MAP/microtubule affinity-regulating kinase  1
74. mdga2: MAM domain containing glycosylphosphatidylinositol anchor 2
75. mpdz: multiple PDZ domain protein
76. mpo: myeloperoxidase
77. mpp7: membrane protein, palmitoylated 7 (MAGUK p55 subfamily member 7)
78. ncam1: neural cell adhesion molecule 1
79. ncam2: neural cell adhesion molecule 2
80. ncdn: neurochondrin
81. ndufs1: NADH dehydrogenase (ubiquinone) Fe-S protein 1, 75 kDa (NADH-coenzyme Q reductase)
82. ndufs7: NADH dehydrogenase (ubiquinone) Fe-S protein 7, 20 kDa (NADH-coenzyme Q reductase)
83. nes: nestin
84. nlgn2: neuroligin 2
85. nlgn3: neuroligin 3
86. npc2: Niemann-Pick disease, type C2
87. npdc1: neural proliferation, differentiation and control, 1
88. npy5r: neuropeptide Y receptor Y5
89. nsf: N-ethylmaleimide-sensitive factor
90. padi2: peptidyl arginine deiminase, type II
91. plcb4: phospholipase C, beta 4
92. pxn: paxillin
93. rab5a: RAB5A, member RAS oncogene family
94. rgs4: regulator of G-protein signaling 4
95. rgs7: regulator of G-protein signaling 7
96. rims1: regulating synaptic membrane exocytosis 1
97. rora: RAR-related orphan receptor A
98. sh3gl1: SH3-domain GRB2-like 1
99. sod2: superoxide dismutase 2, mitochondrial
100. sst: somatostatin
101. vldlr: very low density lipoprotein receptor
102. camkk1: calcium/calmodulin-dependent protein kinase kinase 1, alpha
103. cacna2d2: calcium channel, voltage-dependent, alpha 2/delta subunit 2
104. vcp: valosin-containing protein
105. stx1a: syntaxin 1A (brain)
106. vgf: VGF nerve growth factor inducible
107. calb2: calbindin 2
108. prdx1: peroxiredoxin 1
109. stmn2: stathmin-like 2
110. vsnl1: visinin-like 1
111. cdh2: cadherin 2, type 1, N-cadherin (neuronal)
112. dbn1: drebrin 1
113. oxr1: oxidation resistance 1
114. gpsm1: G-protein signaling modulator 1 (AGS3-like, C. elegans)
115. gmfb: glia maturation factor, beta
116. cd2ap: CD2-associated protein
117. centa1: ArfGAP with dual PH domains 1
118. ncstn: nicastrin
119. pak3: p21 protein (Cdc42/Rac)-activated kinase 3
120. sncb: synuclein, beta
121. calb1: calbindin 1
122. cnr1: cannabinoid receptor 1 (brain)
123. disc1: disrupted in schizophrenia 1
124. dlgap2: discs, large (Drosophila) homolog-associated protein 2
125. dlgap4: discs, large (Drosophila) homolog-associated protein 4
126. dtnb: dystrobrevin, beta
127. dvl1: dishevelled, dsh homolog 1 (Drosophila)
128. exoc7: exocyst complex component 7
129. gabbr1: gamma-aminobutyric acid (GABA) B receptor, 1
130. panx1: pannexin 1
131. pclo: piccolo (presynaptic cytomatrix protein)
132. phactr3: phosphatase and actin regulator 3
133. picalm: phosphatidylinositol binding clathrin assembly protein
134. shank1: SH3 and multiple ankyrin repeat domains 1
135. snap91: synaptosomal-associated protein, 91 kDa homolog (mouse)
136. stmn4: stathmin-like 4
137. sod1: superoxide dismutase 1, soluble
138. synj2: synaptojanin 2
139. tub: tubby homolog (mouse)
140. snca: synuclein, alpha (non A4 component of amyloid precursor)
141. park7: Parkinson disease (autosomal recessive, early onset) 7
142. pcsk1n: proprotein convertase subtilisin/kexin type 1 inhibitor
143. palm: Paralemmin
144. vapb: VAMP (vesicle-associated membrane protein)-associated protein B and C
145. cttn: cortactin
146. cacna1a: calcium channel, voltage-dependent, P/Q type, alpha 1A subunit
147. celsr3: cadherin, EGF LAG seven-pass G-type receptor 3 (flamingo homolog, Drosophila)
148. chl1: cell adhesion molecule with homology to L1CAM (close homolog of L1)
149. chrm5: cholinergic receptor, muscarinic 5
150. cnksr2: connector enhancer of kinase suppressor of Ras 2
151. cnp: 2^'^,3^'^-cyclic nucleotide 3^'^ phosphodiesterase
152. cntfr: ciliary neurotrophic factor receptor
153. cntn1: contactin 1
154. cplx1: complexin 1
155. dag1: dystroglycan 1 (dystrophin-associated glycoprotein 1)
156. dbndd2: dysbindin (dystrobrevin binding protein 1) domain containing 2
157. dbnl: drebrin-like
158. dclk2: doublecortin-like kinase 2
159. dlgap1: discs, large (Drosophila) homolog-associated protein 1
160. dscam: Down syndrome cell adhesion molecule
161. ecel1: endothelin converting enzyme-like 1
162. efnb1: ephrin-B1
163. eml5: echinoderm microtubule associated protein like 5
164. epha3: EPH receptor A3
165. epha5: EPH receptor A5
166. epha7: EPH receptor A7
167. ephb3: EPH receptor B3
168. erbb4: v-erb-a erythroblastic leukemia viral oncogene homolog 4 (avian)
169. evl: Enah/Vasp-like
170. exoc5: exocyst complex component 5
171. fzd9: frizzled homolog 9 (Drosophila)
172. GABARAP: GABA(A) receptor-associated protein
173. gabarapl2: GABA(A) receptor-associated protein-like  2
174. gabrr3: gamma-aminobutyric acid (GABA) A receptor, alpha 3
175. gcgr: glucagon receptor
176. gng7: guanine nucleotide binding protein (G protein), gamma 7
177. got1: glutamic-oxaloacetic transaminase 1, soluble (aspartate aminotransferase 1)
178. got2: glutamic-oxaloacetic transaminase 2, mitochondrial (aspartate aminotransferase 2)
179. gprc6a: G protein-coupled receptor, family C, group 6, member A
180. gria1: glutamate receptor, ionotropic, AMPA 1
181. grid2: glutamate receptor, ionotropic, delta 2
182. grik4: glutamate receptor, ionotropic, kainate 4
183. grin2a: glutamate receptor, ionotropic, N-methyl D-aspartate 2A
184. grip1: glutamate receptor interacting protein 1
185. gripap1: GRIP1 associated protein 1
186. grk6: G protein-coupled receptor kinase 6
187. opa1: optic atrophy 1 (autosomal dominant)
188. pak1: p21 protein (Cdc42/Rac)-activated kinase 1
189. pak2: p21 protein (Cdc42/Rac)-activated kinase 2
190. pawr: PRKC, apoptosis, WT1, regulator
191. phactr1: phosphatase and actin regulator 1
192. pip5k1c: phosphatidylinositol-4-phosphate 5-kinase, type I, gamma
193. plcb1: phospholipase C, beta 1 (phosphoinositide-specific)
194. pld3: phospholipase D family, member 3
195. ppfia3: protein tyrosine phosphatase, receptor type, f polypeptide (PTPRF), interacting protein (liprin), alpha 3
196. ppid: peptidylprolyl isomerase D
197. ppp2cb: protein phosphatase 2 (formerly 2A), catalytic subunit, beta isoform
198. ppp2r2a: protein phosphatase 2 (formerly 2A), regulatory subunit B, alpha isoform
199. prkar2b: protein kinase, cAMP-dependent, regulatory, type II, beta
200. prnp: prion protein
201. ptk2: PTK2 protein tyrosine kinase 2
202. ptn: pleiotrophin
203. ryr3: ryanodine receptor 3
204. sdcbp: syndecan binding protein (syntenin)
205. sh3gl3: SH3-domain GRB2-like 3
206. shank2: SH3 and multiple ankyrin repeat domains 2
207. sirt2: sirtuin (silent mating type information regulation 2 homolog) 2 (S. cerevisiae)
208. smu1: smu-1 suppressor of mec-8 and unc-52 homolog (C. elegans)
209. soat1: sterol O-acyltransferase 1
210. sstr2: somatostatin receptor 2
211. ssx2ip: synovial sarcoma, X breakpoint 2 interacting protein
212. stau1: staufen, RNA binding protein, homolog 1 (Drosophila)
213. stmn3: stathmin-like 3
214. strn: striatin, calmodulin binding protein
215. syn1: synapsin I
216. syngap1: synaptic Ras GTPase activating protein 1 homolog (rat)
217. synj2bp: synaptojanin 2 binding protein
218. synpo: synaptopodin
219. syt11: synaptotagmin XI
220. trap1: TNF receptor-associated protein 1
221. trpc2: transient receptor potential cation channel, subfamily C, member 2 (pseudogene)
222. trpc4: transient receptor potential cation channel, subfamily C, member 4
223. trpc5: transient receptor potential cation channel, subfamily C, member 5
224. wnt7a: wingless-type MMTV integration site family, member 7A
225. tuba1a: tubulin, alpha 1a
226. vipr2: vasoactive intestinal peptide receptor 2
227. trak2: trafficking protein, kinesin binding 2
228. snx17: sorting nexin 17
229. psd: pleckstrin and Sec7 domain containing
230. ppp2r2b: protein phosphatase 2 (formerly 2A), regulatory subunit B, beta isoform
231. ppp1r9b: protein phosphatase 1, regulatory (inhibitor) subunit 9B
232. grm5: glutamate receptor, metabotropic 5
233. grk5: G protein-coupled receptor kinase 5
234. grin2b: glutamate receptor, ionotropic, N-methyl D-aspartate 2B
235. grik1: glutamate receptor, ionotropic, kainate 1
236. grasp: GRP1 (general receptor for phosphoinositides 1)-associated scaffold protein
237. gipc1: GIPC PDZ domain containing family, member  1
238. dpysl4: dihydropyrimidinase-like 4
239. dpysl3: dihydropyrimidinase-like 3
240. dgkb: diacylglycerol kinase, beta 90 kDa
241. ddn: dendrin
242. cit: citron (rho-interacting, serine/threonine kinase 21)
243. grm4: glutamate receptor, metabotropic 4
244. ctnnd2: catenin (cadherin-associated protein), delta 2 (neural plakophilin-related arm-repeat protein).

### I.

Receptor-specific protein interaction networks in lipid raft extracts from control animals. IPA-generated receptor-specific protein lists from control lipid raft samples were clustered into coherent functional interaction networks. Focus molecules (**BOLD** in “molecules in network”) denote the proteins that are present in the predicted reaction network as well as the experimental input protein set. The following are different types of interaction networks.

(1) Control: *cell signaling, nucleic acid metabolism, small molecule biochemistry; *score: 32; focus molecule: 15; molecules in network: ADCY, Akt, Beta-Arrestin, **CD4**, **CHRM5**, **CNTFR**, Creb, **EDNRB**, ERK1/2, **FSHR**, **GABBR1**, **GCGR**, Gpcr, GPR3, GPR12, GPR20, GPR34, GPR65, GPR161, **GRM5**, hCG, Mapk, **OPRD1**, OVGP1, P2RY6, P2RY11, PDGF BB, PI3K, Pkc(s), **PTGER2**, **SFRP4**, **SSTR2**, **THBD**, **UNC5B**, **VIPR2**.

(2) Control: *infectious disease, antigen presentation, antimicrobial response; *score: 31; focus molecule: 15; molecules in network: Ap1, **CHRNB3**, **CHRNB4**, **CHRNG**, **CLDN4**, **CNR1**, **CXADR**, G alphai, Ifn, IFN Beta, IFN TYPE 1, IgG, IKK (complex), IL-1R/TLR, IL12 (complex), Il12 (family), Interferon alpha, IRAK, IRF, NFkB (complex), NRG, **OSMR**, P38 MAPK, **SMO**, **TACR1**, Tlr, **TLR3**, **TLR4**, **TLR5**, **TLR6**, **TLR9**, **TSHR**, Ubiquitin.

(3) Control: *cellular development tumor morphology, cell death; *score: 18; focus molecule: 10; molecules in network: APOL5, ARR3, ARTN, ASB16, Cadherin (E,N,P,VE), **CELSR2**, **CELSR3**, CTNNB1, CTNN*β*-CDHE/N, **DAG1**, EVX1, **FZD9**, GDNF, **GFRA1**, **GFRA3**, GLTSCR1, **GPRC6A**, GRB2, **GRM4**, HRH4, KCNN1, **OPN1LW**, OPN1SW, PALM2-AKAP2, PHACTR2, PRICKLE3, **PTH2R**, SAG, SEPN1, SHROOM2, SRC.

(4) Control: *carbohydrate metabolism; *score: 2; focus molecule: 1; molecules in network: **CLEC4A**, IL13.

(5) Control: *cell-to-cell signaling and interaction, nervous system development and function; *score: 2; focus molecule: 1; molecules in network: **PRPH2**, ROM1.

(6) Control: *protein synthesis, molecular transport, protein trafficking; *score: 2; focus molecule: 1; molecules in network: **GABRR3**, PRKCZ, SQSTM1.

### J.

Receptor-specific protein interaction networks in lipid raft extracts from 3xTgAD animals. IPA-generated receptor-specific protein lists from 3xTgAD lipid raft samples were clustered into coherent functional interaction networks. Focus molecules (BOLD in “molecules in network”) denote the proteins that are present in the predicted reaction network as well as the experimental input protein set. The following are different types of interaction networks.

(1) 3xTgAD: *metabolic disease, endocrine system disorders, cell signaling; *score: 45; focus molecule: 18; molecules in network: **ADCYAP1R1**, Ap1, **CD36**, **CD86**, **CNR1**, Creb, CREB-NFkB, **CXADR**, ERK, ERK1/2, **GABBR1**, **GPR56**, hCG, **HTR4**, **HTR2C**, **IGF1R**, **IGF2R**, IgG, IL12 (complex), **IL1R2**, Insulin, Jnk, LDL, **LIFR**, **LRPAP1**, Mapk, MIP1, NFkB (complex), **NPR2**, OVGP1, P38 MAPK, Pkc(s), **TLR5**, **TNFRSF14**, **TSHR**.

(2) 3xTgAD: *cell signaling, nucleic acid metabolism, small molecule biochemistry; *score: 13; focus molecule: 7; molecules in network: AATK, ABR, AKR1A1, BPI, CACNG5, CRYM, cyclic AMP, CYP26B1, DHRS3, DLG4, GH1, Histone h3, HSD17B11, KCTD11, KIF3C, LAP3, **LPHN1**, **LPHN2**, **OPN1LW**, OPN1SW, P2RY11, **PTCH1**, **PTH2R**, RLN3, RN5S, **ROBO1**, ROS1, **RXFP1**, RXFP2, SERPINB8, TMEM49, TNF, USP3.

(3) 3xTgAD: *cell signaling, cellular function and maintenance, molecular transport; *score: 2; focus molecule: 1; molecules in network: CFTR, FREQ, **IL1RAPL1**, MYD88.

(4) 3xTgAD: *cell-to-cell signaling and interaction, cellular function and maintenance, cellular movement; *score: 2; focus molecule: 1; molecules in network: **GPR1**, PAX3, PRDM5.

(5) 3xTgAD: *behavior, digestive system development and function, cell morphology; *score: 2; focus molecule: 1; molecules in network: NPY, **NPY5R**, PPY, PYY, SSB.

(6) 3xTgAD: *cancer, reproductive system disease, gene expression; *score: 2; focus molecule: 1; molecules in network: FOS, MIR103-1, MIR103-2, MIR107, MIRLET7G, MYC, **OMG**, RHOA, RTN4R.
