# Supplementary material for: Complex and Multidimensional Lipid Raft Alterations in a Murine Model of Alzheimer's Disease
Source: Int J Alzheimers Dis. 2010 Dec 2;2010:604792. doi: 10.4061/2010/604792 (PMC2997345; doi:10.4061/2010/604792)
Supplement: Supplementary file 1 — Supplementary Table 1: the table illustrates the top 50 most upregulated and top 50 most downregulated (italics) proteins (commonly identified in 3xTgAD and control WT) in 3xTgAD cortical raft extracts compared to WT. Ranking was based on relative expression ratios generated by spectral counting for 3xTgAD:WT proteins that displayed a >95% identity confidence. Proteins ranked 1-50 for 3xTgAD>WT (increased in 3xTgAD compared to WT) represent those with expression ratios (3xTgAD:WT) greater than unity while proteins ranked 1-50 for WT>3xTgAD (decreased in 3xTgAD compared to WT: italics) represents those with expression ratios (3xTgAD:WT) less than unity. In each case the most up or downregulated proteins are ranked 1. [file 604792.f1.pdf]

Supplementary Table 1. Differentially regulated proteins represented in both control wild-type (WT) and 3xTgAD raft samples. The table illustrates the top 50 most upregulated and top 50 most downregulated (*italics*) proteins (commonly identified in 3xTgAD and control WT) in 3xTgAD cortical raft extracts compared to WT. Ranking was based on relative expression ratios generated by spectral counting for 3xTgAD:WT proteins that displayed a >95% identity confidence. Proteins ranked 1-50 for 3xTgAD>WT (increased in 3xTgAD compared to WT) represent those with expression ratios (3xTgAD:WT) greater than unity while proteins ranked 1-50 for WT>3xTgAD (decreased in 3xTgAD compared to WT: *italics*) represents those with expression ratios (3xTgAD:WT) less than unity. In each case the most up or downregulated proteins are ranked 1.

Symbol	Protein definition	Expression Rank 3xTgAD>WT
Snca	synuclein, alpha (non A4 component of amyloid precursor)	1
Atm	ataxia telangiectasia mutated	2
Cast	calpastatin	3
Pkia	protein kinase (cAMP-dependent, catalytic) inhibitor alpha	4
Cnr1	cannabinoid receptor 1 (brain)	5
Snap25	synaptosomal-associated protein, 25kDa	6
Dgkg	diacylglycerol kinase, gamma 90kDa	7
Stx1a	syntaxin 1A (brain)	8
Cst6	cystatin E/M	9
Sncb	synuclein, beta	10
Pdcl	phosducin-like	11
Cdc5l	CDC5 cell division cycle 5-like (S. pombe)	12
Cdk5rap2	CDK5 regulatory subunit associated protein 2	13
Plk1	polo-like kinase 1 (Drosophila)	14
PGAP1	post-GPI attachment to proteins 1	15
Cul5	cullin 5	16
Cstb	cystatin B (stefin B)	17
Stip1	stress-induced-phosphoprotein 1	18
Csnk1e	casein kinase 1, epsilon	19
Per3	period homolog 3 (Drosophila)	20
Clic4	chloride intracellular channel 4	21
Sca10	ataxin 10	22
Cfl1	cofilin 1 (non-muscle)	23
Prkwnk1	WNK lysine deficient protein kinase 1	24
Dab2ip	DAB2 interacting protein	25
Stmn1	stathmin 1	26
Epn1	epsin 1	27
Ubc	ubiquitin C	28
Nos1	nitric oxide synthase 1 (neuronal)	29
Vdac2	voltage-dependent anion channel 2	30
Pdlim7	PDZ and LIM domain 7 (enigma)	31
Ncstn	nicastrin	32
Vcp	valosin-containing protein	33
Dlgap4	discs, large (Drosophila) homolog-associated protein 4	33
Sycp1	synaptonemal complex protein 1	35
Coro1a	coronin, actin binding protein, 1A	36

Snap91	synaptosomal-associated protein, 91kDa homolog (mouse)	37
Dlgap2	discs, large (Drosophila) homolog-associated protein 2	38
Stxbp3	syntaxin binding protein 3	39
Gdi2	GDP dissociation inhibitor 2	40
Uchl1	ubiquitin carboxyl-terminal esterase L1 (ubiquitin thiolesterase)	41
Gdi1	GDP dissociation inhibitor 1	42
Ubqln1	ubiquilin 1	43
Bzrap1	benzodiazapine receptor (peripheral) associated protein 1	44
Cacna2d2	calcium channel, voltage-dependent, alpha 2/delta subunit 2	45
Park7	Parkinson disease (autosomal recessive, early onset) 7	46
Camkk1	calcium/calmodulin-dependent protein kinase kinase 1, alpha	47
P4hb	prolyl 4-hydroxylase, beta polypeptide	48
Gpr141	G protein-coupled receptor 141	49
Pthr2	parathyroid hormone 2 receptor	50

**Symbol**	**Protein definition**	**Expression Rank WT>3xTgAD**
Ezr	*ezrin*	*1*
Tgfb1	*transforming growth factor, beta 1*	*2*
Centa1	*ArfGAP with dual PH domains 1*	*3*
Tlr5	*toll-like receptor 5*	*4*
Sdfr1	*neuroplastin*	*5*
PRKCQ	*protein kinase C, theta*	*6*
Oxr1	*oxidation resistance 1*	*7*
Dtnb	*dystrobrevin, beta*	*8*
Dbn1	*drebrin 1*	*9*
Gabbr1	*gamma-aminobutyric acid (GABA) B receptor, 1*	*10*
Cnga1	*cyclic nucleotide gated channel alpha 1*	*11*
Prss12	*protease, serine, 12 (neurotrypsin, motopsin)*	*12*
Pak3	*p21 protein (Cdc42/Rac)-activated kinase 3*	*13*
Smoc1	*SPARC related modular calcium binding 1*	*14*
Vgf	*VGF nerve growth factor inducible*	*15*
Drd1ip	*calcyon neuron-specific vesicular protein*	*16*
Crhbp	*corticotropin releasing hormone binding protein*	*17*
Tmeff1	*transmembrane protein with EGF-like and two follistatin-like domains 1*	*18*
Tsc1	*tuberous sclerosis 1*	*19*
Tm9sf2	*transmembrane 9 superfamily member 2*	*20*
Sod2	*superoxide dismutase 2, mitochondrial*	*21*
Dnch1	*dynein, cytoplasmic 1, heavy chain 1*	*22*
Erp29	*endoplasmic reticulum protein 29*	*23*
Cend1	*cell cycle exit and neuronal differentiation 1*	*24*
Vapa	*VAMP (vesicle-associated membrane protein)-associated protein A, 33kDa*	*25*
Cdh10	*cadherin 10, type 2 (T2-cadherin)*	*26*
Vegfa	*vascular endothelial growth factor A*	*27*
Efemp2	*EGF-containing fibulin-like extracellular matrix protein 2*	*28*
Vamp2	*vesicle-associated membrane protein 2 (synaptobrevin 2)*	*29*
Drg1	*developmentally regulated GTP binding protein 1*	*30*
Snx3	*sorting nexin 3*	*31*
Prdx1	*peroxiredoxin 1*	*32*
Rtn4	*reticulon 4*	*33*
Duox1	*dual oxidase 1*	*34*

Cdkn1b	*cyclin-dependent kinase inhibitor 1B (p27, Kip1)*	*35*
Cap1	*CAP, adenylate cyclase-associated protein 1 (yeast)*	*36*
Ppp1r1a	*protein phosphatase 1, regulatory (inhibitor) subunit 1A*	*37*
Ppp1r9a	*protein phosphatase 1, regulatory (inhibitor) subunit 9A*	*38*
Ctnnb1	*catenin (cadherin-associated protein), beta 1, 88kDa*	*39*
Vapb	*VAMP (vesicle-associated membrane protein)-associated protein B and C*	*40*
Gpsm1	*G-protein signaling modulator 1 (AGS3-like, C. elegans)*	*41*
Sycp2	*synaptonemal complex protein 2*	*42*
Egfr	*epidermal growth factor receptor (erythroblastic leukemia viral (v-erb-b) oncogene homolog, avian)*	*43*
Pclo	*piccolo (presynaptic cytomatrix protein)*	*44*
Cp	*ceruloplasmin (ferroxidase)*	*45*
Synj2	*synaptojanin 2*	*46*
Pdap1	*PDGFA associated protein 1*	*47*
Dpp3	*dipeptidyl-peptidase 3*	*48*
Cdh2	*cadherin 2, type 1, N-cadherin (neuronal)*	*49*
Shank1	*SH3 and multiple ankyrin repeat domains 1*	*50*
